# Supplementary material for: Optically induced charge-transfer in donor-acceptor-substituted p- and m- C2B10H12 carboranes
Source: Nat Commun. 2024 Apr 8;15:3005. doi: 10.1038/s41467-024-47384-4 (PMC11001991; doi:10.1038/s41467-024-47384-4)
Supplement: Supplementary file 1 — Supplementary Information [file 41467_2024_47384_MOESM1_ESM.pdf]

Supplementary Information

Optically Induced Charge-Transfer in Donor-Acceptor-Substituted *p*- and *m*-$C_2B_{10}H_{12}$ Carboranes

Lin Wu,[1] Marco Holzapfel,[2] Alexander Schmiedel,[2] Fuwei Peng,[1] Michael Moos,[2] Paul Mentzel,[2] Junqing Shi,[1] Thomas Neubert,[3] Rüdiger Bertermann,[3] Maik Finze,[3] Mark A. Fox,[4] Christoph Lambert,[2,*] Lei Ji[1,*]

[1]*Frontiers Science Center for Flexible Electronics (FSCFE), Shaanxi Institute of Flexible Electronics (SIFE) & Shaanxi Institute of Biomedical Materials and Engineering (SIBME), Northwestern Polytechnical University (NPU), 127 West Youyi Road, Xi'an 710072, China.*

E-mail: iamlji@nwpu.edu.cn

[2]*Institut für Organische Chemie, Julius-Maximilians-Universität Würzburg, Am Hubland, 97074 Würzburg, Germany.*

E-mail: christoph.lambert@uni-wuerzburg.de

[3]*Institut für Anorganische Chemie, Julius-Maximilians-Universität Würzburg, Am Hubland, 97074 Würzburg, Germany.*

[4]*Department of Chemistry, University of Durham, South Road, Durham, DH1 3LE, U.K.*

Table of Contents

1. Experimental Procedures. 3

1.1 General procedures. 3

1.2 Theoretical background. 5

1.3 Synthetic procedures for **DA-*p*Carb**, **DA-*m*Carb**, **DA-*p*Benz** and **DA-*m*Benz**. 7

2. Results and Discussion. 13

2.1 Electrochemistry. 13

2.2 Solution-state photophysics. 16

2.3 Theoretical studies. 32

2.3.1. Optimization at B3LYP/6-31G*. 32

2.3.2 NAO. 37

2.3.3 TD-DFT. 38

2.3.4 Summary. 42

3. NMR Spectra. 43

4. Reference 61

1. Experimental Procedures.

1.1 General procedures.

Dimesitylboron fluoride (Mes_2BF),[1] 4-iodo-*N*,*N*-bis(4-methylphenyl)benzenamine (**1**) and 4-bromo-*N*,*N*-bis(4-methylphenyl)benzenamine (**2**),[2] and *N*,*N*-bis(4-methylphenyl)-4-(4,4,5,5-tetramethyl-1,3,2-dioxaborolan-2-yl)benzenamine (**3**)[3] were synthesized according to literature, other reagents were obtained from commercial sources and used without further purification. Solvents were distilled from sodium under N_2 for ethylene glycol dimethylether (DME), toluene, tetrahydrofuran (THF) and stored over molecular sieves under argon. Glassware, needles, and magnetic stirring bars were dried in oven for several hours before use. All reactions were monitored by thin layer chromatography (TLC), and the spots are identified under UV light 254 nm and 365 nm. Column chromatography was done on silica gel (200-300 mesh). NMR spectra were recorded in $CDCl_3$ and CD_2Cl_2 using Bruker Advance 500 NMR spectrometer (^{1}H, 500 MHz; ^{13}C, 126 MHz; ^{11}B, 128 MHz), all chemical shifts are reported relative to the residual protonated solvent chloroform (7.26 ppm) and dichloromethane (5.32 ppm). Chemical shifts are listed in parts per million (ppm) and coupling constants are listed in Hertz (Hz). The HRMS were recorded using an Orbitrap-FTMS detector with AP-MALDI as the ionization source, and GC-MS were recorded with EI ionization source. Recycling Preparative SEC was performed using a JAI LC-5060 Recycling Preparative HPLC system with JAIGEL-2HR and JAIGEL-2.5HR columns using dichloromethane (DCM) as eluent and monitored by a UV-Vis 4ch 800LA detector.

Photophysical measurements. Absorption spectra were recorded on UH 5700 UV-Vis-NIR spectrophotometer. All luminescence properties were recorded on Edinburgh FLS1000 and FLS980, and all spectra were fully corrected for the spectral response of the instrument. All measurements were performed in standard quartz cuvettes (1 cm × 1 cm). All solutions for photophysical measurements had a concentration of *ca*. 1×10^{-5} M. The absorbance maximum of any solutions in the fluorescence measurements is below 0.15 to avoid re-absorption. The fluorescence quantum yields were measured using a calibrated integrating sphere (150 mm inner diameter). Fluorescence lifetimes were recorded using a time-correlated single-photon counting (TCSPC) method. Solutions were excited at 293.6 nm with a picosecond pulsed diode laser (pulse width: 1231.1 ps) at repetition

rates of 1−5 MHz and lifetime data were recorded at the emission maxima. Decays were recorded to 10000 counts in the peak channel with a record length of at least 4000 channels. The fluorescence decay was analyzed by tail fitting method. The quality of all decay fits was judged to be satisfactory based on the calculated values of the reduced χ^2 and Durbin-Watson parameters and visual inspection of the weighted and autocorrelated residuals.

Femtosecond transient absorption spectroscopy.

All experiments were performed in quartz cuvettes with an optical path length of 1 mm at room temperature. All samples were dissolved in hexane and THF, from spectroscopic grade, filtered and degassed. The optical density was adjusted to *ca*. 0.3 at the corresponding excitation wavelength. The transient absorption spectrometer HELIOS from Ultrafast Systems was pumped by a Newport-Spectra-Physics Solstice laser system with a fundamental wavelength of 800 nm, a pulse length of 100 fs and a repetition rate of 1 kHz.

The output beam from the Solstice was split into two parts. One part was used to produce a white light continuum which was cut by filters to achieve a 700 nm - 350 nm probe spectrum. The second part was used create the excitation pulses within an optical parametric amplifier (TOPAS-C) from Light Conversion with a pulse length of 140 fs at the 340 nm and 388 nm excitation wavelengths.

The computer-controlled stage (retro reflector in double pass setup) set the time delay between pump and probe pulse up to 8 ns with 20 fs intervals from 0 fs to 4 ps and from 4 ps to 8 ns in logarithmic steps with a maximum step width of 200 ps.

Before data analysis, the raw transient data were corrected for stray light and white light dispersion (chirp). The IRF was ca. 150 fs. The evolution associated difference spectra (EADS) were obtained from the corrected data by a global analysis using GLOTARAN software. For further details on the setup see N. Auerhammer et al.[4]

Fluorescence upconversion

All measurements were performed as described in the femtosecond transient absorption spectroscopy part. The broadband fluorescence upconversion set-up (FLUPS) from LIOPTEC was pumped by the Solstice as well. The pump and the gate pulses were generated by two NOPAs (home built after E. Riedle) from which the pump pulse was doubled in frequency by a BBO crystal to obtain the excitation wavelength. The wavelength range from the spectrometer for the fluorescence reaches from 395 – 850 nm with a resolution of 0.9 nm (303 – 516 nm upconverted, intrinsic resolution 0,42 nm). The excitation pulses had 220 nJ @ 340 nm and the gate pulses had 40 µJ @ 1310 nm. To filter the remaining pump pulse after exciting the sample a shortpass filter 425 nm (OD 4) was used. For further details see Mieczkowski, M et al.[5]

Calculation details.

All calculations (DFT and TD-DFT) were carried out with Gaussian 16 (Revision A. 03) program package.[6, 7] The ground-state geometries were optimized using B3LYP functional on the 6-31G* level of theory. The optimized geometries were confirmed to be local minima by performing frequency calculations and obtaining only positive frequencies. Based on these optimized structures, gas-phase vertical transitions were calculated (singlets, 15 states) by TD-DFT using B3LYP functional in combination with the 6-31G* basis set. The orbital overlap was quantified using Multiwfn[8] by integrating the overlap of the modes of different orbitals.

1.2 Theoretical background.

Lippert-Mataga plots. The Lippert-Mataga equation[9] for the solvatochromic shift is expressed as follows:

$$\tilde{\nu}_{\text{fluor}} = \tilde{\nu}_{\text{abs}} - \frac{2\Delta f}{hca^3}(\mu_{\text{e}} - \mu_{\text{g}})^2 + \text{const.}$$

$$\Delta f = \frac{\varepsilon - 1}{2\varepsilon + 1} - \frac{n^2 - 1}{2n^2 + 1}$$

While the slope is:

$$slope = -\frac{2(\mu_e - \mu_g)^2}{hca^3}$$

In the Lippert-Mataga equation, a denotes the radius of the Onsager cavity around the fluorophore, n, and ε are the solvent dielectric constant and the refraction index, respectively. μ_{e} and μ_{g} are the dipole moments in the excited and ground state, respectively, $\tilde{\nu}_{\text{fluor}}$ and $\tilde{\nu}_{\text{abs}}$ are the fluorescence and absorption wavelength (expressed in cm^{-1} units), c is the velocity of light, and h is Plank's constant. The Onsager cavity a and μ_{g} are estimated from quantum chemical calculations by using density functional theory method at B3LYP/6-31G* level.

Jortner's theory. Jortner's theory was applied to have a closer investigation of CT character, where λ_{v} and λ_0 are the inner reorganization energy and outer reorganization energy, respectively. $\tilde{\nu}_{\text{v}}$ is the average molecular vibrational mode, and ΔG^{00} is the difference in the free energy between the diabatic ground and the excited state.[10] All of these parameters are obtained from the least-squares fits of the emission band of **DA-*p*Carb** and **DA-*m*Carb** in different solvents.

$$\varepsilon = \frac{8N\pi^3}{3000h\, ln\, 10} n\tilde{\nu}\mu_{\text{eg}}^2 \sum_{j=0}^{\infty} \frac{e^{-S}\, s^j}{j!} \sqrt{\frac{1}{4\pi\lambda_0 RT}}\, exp[-\frac{(j\tilde{\nu}_{\text{v}} + \lambda_0 - \tilde{\nu} + \Delta G^{00})^2}{4\pi\lambda_0 RT}]$$

While the Huang-Rhys factor S is:

$$S = \frac{\lambda_{\text{v}}}{\tilde{\nu}_{\text{v}}}$$

Strickler-Berg equation. The squared transition dipole moments of the ICT transitions in the absorption spectra $\boldsymbol{\mu}^{\mathbf{2}}_{\text{abs}}$ and emission spectra $\boldsymbol{\mu}^{\mathbf{2}}_{\mathbf{fl}}$ in different solvents were determined from the radiative decay rate constant by the Strickler-Berg equation.[11, 12]

$$\mu^2_{\text{abs}} = \frac{3\varepsilon_0 hC\, ln\, 10}{20\pi^2 N_{\text{A}}} \cdot \frac{9n}{(n^2+2)^2} \int \frac{\varepsilon}{\tilde{\nu}} d\tilde{\nu}$$

and

$$\mu^2_{\text{fl}} = \frac{\phi}{\bar{\tau}} \cdot \frac{3\varepsilon_0 h}{16 \cdot 10^6 \pi^3} \cdot \frac{9}{n(n^2+2)^2} \cdot \frac{\int \frac{F(\tilde{\nu})}{\nu^3} d\tilde{\nu}}{\int F(\tilde{\nu})\, d\tilde{\nu}}$$

where ε_0 is the vacuum permittivity, h is the Planck constant, c is the speed of light, N_A is the Avogadro constant, n is the index of refraction of the solvent, ε is the extinction coefficient in $M^{-1}cm^{-1}$, $\tilde{\nu}$ is the wavenumber in cm^{-1}, ϕ is the quantum yield, and $\bar{\tau}$ is fluorescence lifetime.

Marcus-Hush theory. The electronic coupling matrix element V between excited and ground state was obtained by Marcus-Hush theory. The electron transfer coupling is evaluated from the averaged fluorescence energy $\tilde{\nu}_{\text{av}}$, the transition moment of fluorescence μ_{fl} (from Strickler-Berg equation) and the dipole moment difference $\Delta\mu$ (from the Lippert-Matag plots)

$$V = \frac{\mu_{\text{fl}} \tilde{\nu}_{\text{av}}}{\Delta\mu}$$

While the average fluorescence energy $\tilde{\nu}_{\text{av}}$ is calculated by:

$$\tilde{\nu}_{\text{av}} \approx 3\sqrt{\left\langle \tilde{\nu}_{\text{fl}}^{-3} \right\rangle_{\text{av}}^{-1}}$$

$$\left\langle \tilde{\nu}_{\text{fl}}^{-3} \right\rangle_{\text{av}}^{-1} = \int I_{\text{f}}\, d\tilde{\nu} \int \tilde{\nu}^{-3} I_{\text{f}}\, d\tilde{\nu}$$

in which I_{f} expressed in photos per unit time per unit spectral energy.

1.3 Synthetic procedures for DA-*p*Carb, DA-*m*Carb, DA-*p*Benz and DA-*m*Benz.

4,4'-dimethyltriphenylamino-carborane (**D-*p*Carb** and **D-*m*Carb**) was synthesized via coupling of lithiated carborane and 4-iodotriarylamine, giving colorless flakes with a yield of 60%. Further lithiation of **D-Carb** could be done at elevated temperature (80 °C) within 8 h, and **DA-Carb** was formed after addition of Mes_2BF. The reference compounds **DA-*p*Benz** and **DA-*m*Benz** were synthesized via similar lithiation-borylation procedure,[13] which were then synthesized by Buchwald-Hartwig coupling. While **DA-Benz** is bench stable, **DA-Carb** tends to decompose on silica, but could be isolated successfully by recycling GPC with moderate yield. Due to the sensitivity of **DA-Carb** towards water and protic solvents, all photophysical measurements were performed under argon atmosphere.

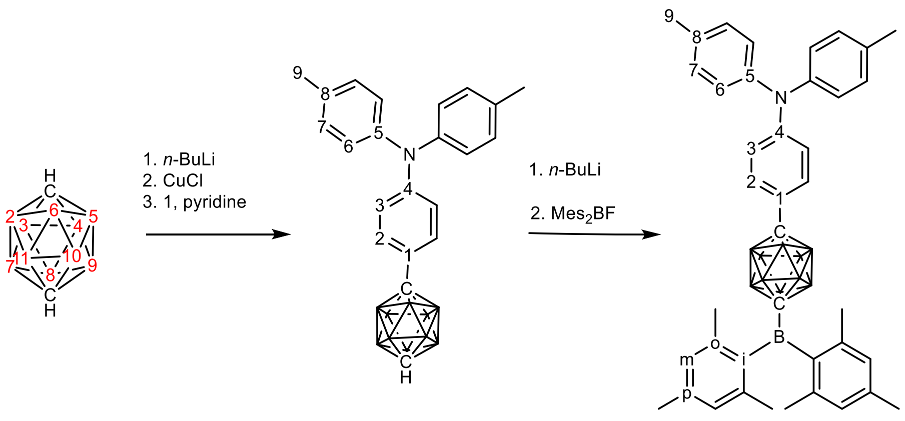

D-*p*Carb **DA-*p*Carb**

Supplementary Fig. 1: Synthetic route of **D-*p*Carb** and **DA-*p*Carb**.

Synthesis of D-*p*Carb.

D-*p*Carb was prepared in a modified method condition with literature.[14] *p*-Carborane (144 mg, 1 mmol) was placed in an argon-filled Young's tube. Dry, degassed DME (10 ml) were added under nitrogen atmosphere and a 2.5 M *n*-BuLi solution in hexane (0.40 mL, 1.00 mmol) was added dropwise at 0 °C. The mixture was then stirred for an hour, and dry CuCl (110 mg, 1.11 mmol) was added in one portion and the resulting solution was stirred at room temperature for an hour. Pyridine (0.6 mL) and compound **1** (0.40 g,1 mmol) were added in one portion and the reaction mixture was refluxed under a nitrogen atmosphere for 24 h. After being cooled to room temperature, the reaction was quenched by water (1 mL), and the solvent was removed under reduced pressure and the crude

product was purified on silica gel chromatography using petroleum ether as eluent to afford **D-*p*Carb** as colorless crystals (250 mg, 60.1 %).^{11}B NMR (160 MHz, chloroform-*d*): δ = -12.26 (d, J = 165.5 Hz, B2-6), -15.05 (d, J = 165.5 Hz, B7-11) ppm; ^{11}B{^{1}H} NMR (160 MHz, chloroform-*d*): δ = -12.30 (s, B2-6), -15.09 (s, B7-11) ppm; ^{1}H NMR (500 MHz, chloroform-*d*) δ = 7.05 (d, J = 8.2 Hz, 4H, C7H), 6.97-6.94 (m, 6H, C2H, C6H), 6.73 (d, J = 8.7 Hz, 2H, C3H), 3.10–2.42 (m, 6H) (cage CH + cage BH), 2.30 (s, 6H) (C9H$_3$), 2.22–1.60 (m, 5H) (cage BH) ppm; ^{1}H{^{11}B} NMR (500 MHz, chloroform-*d*) δ = 7.05 (d, J = 8.0 Hz, 4H, C7H), 6.97-6.94 (m, 6H, C2H, C6H), 6.73 (d, J = 8.7 Hz, 2H, C3H), 2.74 (s, 1H, cage CH), 2.50 (s, 5H, B2-5H), 2.30 (s, 6H, C9H$_3$), 2.26 (s, 5H, B7-11H) ppm. BH peaks were assigned based on reported monoaryl-*p*Carb data.[15] GC-MS (EI$^+$) *m/z*: [*M*]$^+$ calcd for $C_{22}H_{29}B_{10}N$, 415; found, 415.

Synthesis of DA-*p*Carb.

D-*p*Carb (100 mg, 0.24 mmol) was dissolved in toluene (5 mL) and a 2.5 M *n*-BuLi solution in hexane (0.12 mL, 0.29 mmol) was added dropwise at –78 °C under nitrogen. The reaction mixture was warmed to room temperature slowly and stirred at 80 °C overnight. The reaction was cooled again to –78 °C and Mes$_2$BF (77 mg, 0.29 mmol) in toluene (2 mL) was added dropwise. The reaction mixture was then stirred at room temperature for 4 d. The suspension was filtered through a short chromatographic pack and then purified on HPLC to afford **DA-*p*Carb** as yellow-green solids (23 mg, 14%). ^{11}B NMR (160 MHz, chloroform-*d*): δ = -11.31 (d, 5B, B2-6), -12.23 (d, 5B, B7-11) ppm; ^{11}B{^{1}H} NMR (160 MHz, chloroform-d): δ = 85.21 (s) (br s, 1B, *B*Mes$_2$), -11.55 (s, 5B, B2-6), -12.04 (s, 5B, B7-11) (B_{cage}H) ppm; ^{1}H NMR (500 MHz, chloroform-*d*) δ = 7.03 (d, J = 8.2 Hz, 4H, C7H), 6.94 - 6.91 (m, 6H, C2H, C6H), 6.73 (s, 4H, mC*H* of BMes$_2$), 6.71 (d, J = 9.0 Hz, 2H, C3H), 3.16–2.53 (m, 7H) (cage B*H*), 2.36 (s, 12H, oC*H*$_3$ of BMes$_2$), 2.29 (s, 6H, C9*H*$_3$), 2.22 (s, 6H, pC*H*$_3$ of BMes$_2$), 2.14–1.89 (m, 3H) (cage B*H*) ppm; ^{1}H{^{11}B} NMR (500 MHz, chloroform-*d*): δ = 7.03 (d, J = 8.0 Hz, 4H, C7H), 6.93 (d, J = 9.0 Hz, 2H, C2H), 6.92 (d, J = 8.0 Hz, 4H, C6H), 6.73 (s, 4H, mC*H* of BMes$_2$), 6.71 (d, J = 9.0 Hz, 2H, C3H), 2.56 (s, 4H, BH), 2.45 (s, 4H, BH), 2.36 (s, 13H, B*H*, oC*H*$_3$ of BMes$_2$), 2.29 (s, 7H, B*H*, C9*H*$_3$), 2.22 (s, 6H, pC*H*$_3$ of BMes$_2$) ppm; ^{13}C{^{1}H} NMR (126 MHz, chloroform-*d*) δ = 148.4 (C4), 144.8 (C5), 139.8 (iC of BMes$_2$), 138.9 (oC of BMes$_2$), 138.3 (pC of BMes$_2$), 133.3 (C8), 130.0 (C7), 129.7 (C1), 139.3(mC of BMes$_2$), 127.5 (C2), 125.1 (C6), 120.4 (C3), 92.5 (cage C), 79.3 (cage *C* of BMes$_2$), 26.9 (o*C*H$_3$ of BMes$_2$), 21.0 (C9), 20.8

(pCH_3 of $BMes_2$) ppm. BH peaks were assigned with the aid of reported diaryl-*p*Carb data.[15] NMR peaks corresponding to the $BMes_2$ group were assigned with the aid of reported NMR data for Mes_2B carboranes.[16] HRMS (AP-MALDI$^+$) *m/z*: $[M]^+$ calcd for $C_{40}H_{50}B_{11}N$, 663.5040; found, 663.5034 (|Δ| = 0.09 ppm).

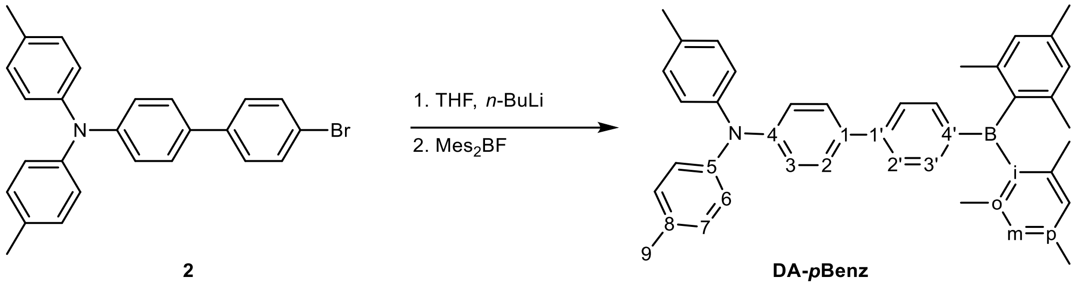

Supplementary Fig. 2: Synthetic route of **DA-*p*Benz**.

Synthesis of DA-*p*Benz.

Compound **2** (107 mg, 0.25 mmol) was placed in a Young's tube and dried under vacuum and then backfilled with argon before THF (5 mL) was added, and a solution of *n*-BuLi in hexane (1.6 M, 0.18 mL, 0.30 mmol) was added dropwise at –78 °C under argon atmosphere. The suspension was stirred for a further 2 h at –78 °C. Mes_2BF (100 mg, 0.37 mmol) in toluene was then added dropwise and the reaction was warmed to room temperature and stirred overnight. After quenching with water (1 mL), the suspension was filtered through a short silica pack, then the solvent was removed under reduced pressure and the crude product was purified on silica column using petroleum ether/dichloromethane = 50:1 as eluent to afford **DA-*p*Benz** as yellow crystals (99 mg, 66%). ^{1}H NMR (500 MHz, chloroform-*d*) δ = 7.56 (s, 4H, C2′H + C3′H), 7.51 (d, *J* = 8.5, 2H, C2H), 7.09 (d, *J* = 8.5 Hz, 4H, C7H), 7.08 (d, *J* = 8.7 Hz, 2H, C3H), 7.04 (d, *J* = 8.5 Hz, 4H, C6H), 6.84 (s, 4H, mC*H* of $BMes_2$), 2.34 (s, 6H, pCH_3 of $BMes_2$), 2.33 (s, 6H, C9H_3), 2.05 (s, 12H, oCH_3 of $BMes_2$) ppm. ^{13}C{^{1}H} NMR (126 MHz, chloroform-*d*) δ = 148.3 (C4), 145.2 (C5), 144.1 (C1′), 143.9 (C4′), 142.0 (iC of $BMes_2$), 141.0 (oC of $BMes_2$), 138.6 (pC of $BMes_2$), 137.4 (C3′), 133.4 (C8), 133.0 (C1), 130.1 (C7), 128.3 (mC of $BMes_2$), 127.9 (C2), 126.0 (C2′), 125.0 (C6), 122.5 (C3), 23.6 (oCH_3 of $BMes_2$), 21.4 (pCH_3 of $BMes_2$), 21.0 (C9) ppm. HRMS (AP-MALDI$^+$) *m/z*: $[M]^+$ calcd for $C_{44}H_{44}BN$, 596.3598; found, 596.3597 (|Δ| = 0.02 ppm).

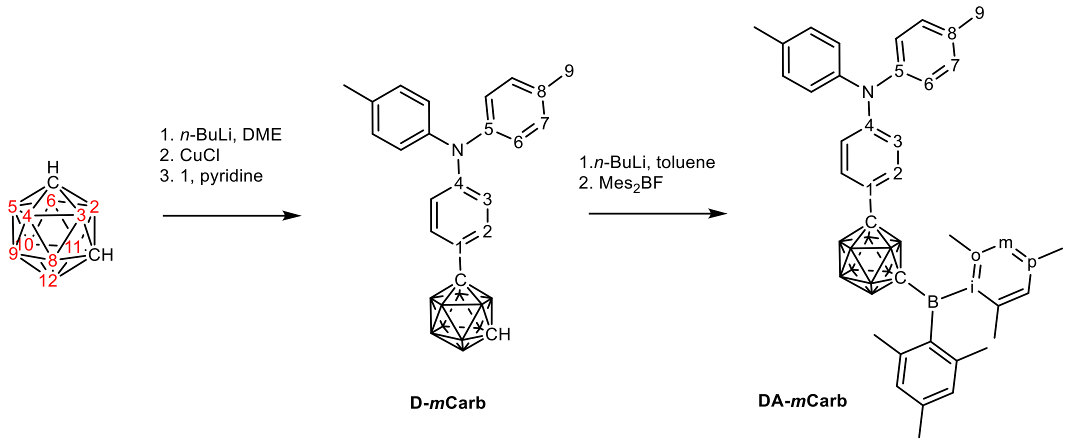

Supplementary Fig. 3: Synthetic route of **D-*m*Carb** and **DA-*m*Carb**.

Synthesis of D-*m*Carb.

D-*m*Carb was synthesized with the same method for **D-*p*Carb** using *m*-carborane instead of *p*-carborane as the starting material. **D-*m*Carb** was afforded as colorless crystals (150 mg, 35%). ^{11}B NMR (160 MHz, chloroform-*d*): δ = -3.92 (d, *J* = 168.5 Hz, B5), -8.60 (s, B12), -10.63 (d, 4B, *J* = 151.6 Hz, B4,6,9,10), -13.57 (d, 2B, *J* = 163.7 Hz, B8,11), -15.16 (d, 2B, *J* = 185.8 Hz, B2,3) ppm; ^{11}B{^{1}H} NMR (160 MHz, chloroform-*d*): δ = -3.91 (s, B5), -9.05 (s, B12), -10.59 (s, B4,6,9,10), -13.54 (s, B8,11), -15.15 (s, B2,3) (B_{cage}H) ppm. ^{1}H NMR (500 MHz, chloroform-*d*) δ = 7.17 (d, *J* = 8.9 Hz, 2H, C2H), 7.07 (d, *J* = 8.2 Hz, 4H, 4H, C7H), 6.98 (d, *J* = 8.3 Hz, 4H, C6H), 6.80 (d, *J* = 8.8 Hz, 2H, C3H), 3.65–2.36 (m, 8H) (cage C*H* + cage B*H*), 2.31 (s, 6H, C9H_3), 2.15–1.66 (m, 3H) (cage B*H*) ppm; ^{1}H{^{11}B} NMR (500 MHz, chloroform-*d*) δ = 7.17 (d, *J* = 8.4 Hz, 2H, C2H), 7.07 (d, *J* = 7.8 Hz, 4H, C7H), 6.98 (d, *J* = 7.7 Hz, 4H, C6H), 6.80 (d, *J* = 8.3 Hz, 2H, C3H), 3.02 (s, 1H, cage C*H*), 2.94 (s, 2H, B2,3H), 2.65 (s, 1H, B5H), 2.49 (s, 3H, B9,10,12H), 2.31 (s, 6H, C9H_3), 2.25 (s, 2H, B8,11H), 2.18 (s, 2H, B4, 6H) ppm. BH peaks were assigned from the closely related monoaryl-*m*Carb data.[17] GC-MS (EI$^+$) *m/z*: [*M*]$^+$ calcd for $C_{22}H_{29}B_{10}N$, 415; found, 415.

Synthesis of DA-*m*Carb.

DA-*m*Carb was made using the same method as for **DA-*p*Carb** with **D-*m*Carb** as starting material. **DA-*m*Carb** was achieved as pale-yellow solids (20 mg, 13%). ^{11}B NMR (160 MHz, chloroform-*d*): δ = -0.14 (d, 1B, B5), -9.83 (m, 9B, B2,3,4,6,8,9,10,11,12) ppm, ^{11}B{^{1}H} NMR (160 MHz, chloroform-*d*): δ = 0.13 (d, 1B, B5), -9.34 (m, 9B, B2,3,4,6,8,9,10,11,12) ppm, the BMes$_2$-*B* was not observed due to rapid quadrupolar relaxation of the boron atom. ^{1}H NMR (500 MHz, chloroform-*d*):

δ = 7.16 (d, *J* = 8.9 Hz, 2H, C2H), 7.10 (d, *J* = 8.3 Hz, 4H, C7H), 7.00 (d, *J* = 8.4 Hz, 4H, C6H), 6.82 (s, 2H, C3H), 6.80 (s, 4H, mC*H* of $BMes_2$), 3.61–2.10 (m, 34H) (cage B*H*, CH_3), 2.47 (s, 12H, oCH_3 of $BMes_2$), 2.34 (s, 6H, C9H), 2.27 (s, 6H, pCH_3 of $BMes_2$) ppm; $^{1}H\{^{11}B\}$ NMR (500 MHz, chloroform-*d*) *δ* = 7.16 (d, *J* = 8.7 Hz, 2H, C2H), 7.09 (d, *J* = 8.0 Hz, 4H, C7H), 7.00 (d, *J* = 8.1 Hz, 4H, C6H), 6.81 (d, *J* = 8.7 Hz, 2H, C3H), 6.80 (s, 4H, mC*H* of $BMes_2$), 3.10 (s, 2H, B4,6H), 2.86 (s, 2H, B8,11H), 2.60 (s, 4H, 4H, B2,3,9,10H), 2.47 (s, 12H, oCH_3 of $BMes_2$), 2.42 (s, 1H, B5H), 2.34 (s, 6H, C9H_3), 2.31 (s, 1H, B12H), 2.27 (s, 6H, pCH_3 of $BMes_2$). $^{13}C\{^{1}H\}$ NMR (126 MHz, dichloromethane-d_2): δ = 149.1 (C4), 145.0 (C5), 140.0 (iC of $BMes_2$), 139.3 (oC of $BMes_2$), 139.1 (pC of $BMes_2$), 134.0 (C8), 130.4 (C7), 129.7 (mC of $BMes_2$), 128.7 (C2), 127.9 (C1), 125.7 (C6), 120.5 (C3), 80.6 (cage C), 76.4 (cage C of $BMes_2$), 27.0 (oCH_3 of $BMes_2$), 20.9 (C9), 20.8 (pCH_3 of $BMes_2$) ppm. BH peaks were assigned with the aid of related diaryl-*m*Carb data.[18] NMR peaks of the $BMes_2$ group were assigned with the aid of reported NMR data for Mes_2B carboranes.[16] HRMS (AP-MALDI$^+$) *m/z*: [*M*]$^+$ calcd for $C_{40}H_{50}B_{11}N$, 663.5032; found, 663.5034 (|Δ| = 0.03 ppm).

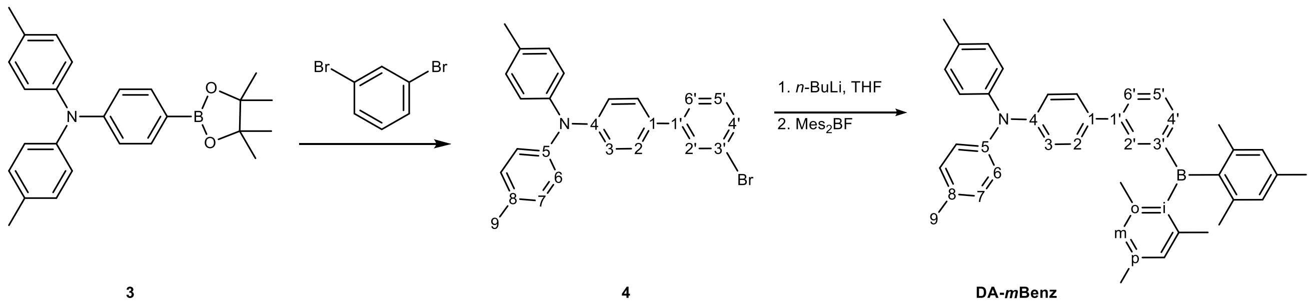

Supplementary Fig. 4: Synthetic route of **DA-*m*Benz**.

Synthesis of 4.

Compound **3** (400 mg, 1 mmol), 1,3-dibromobenzene (283 mg, 1.2 mmol), $Pd(PPh_3)_4$ (120 mg, 0.10 mmol), K_2CO_3 (912 mg, 6.60 mmol) were dissolved in a mixture of degassed toluene (30 mL), THF (5 mL), and H_2O (2 mL). Then the mixture was refluxed under a nitrogen atmosphere overnight. After being cooled to room temperature, the mixture was extracted with dichloromethane and H_2O. The organic layer was dried over anhydrous $MgSO_4$, and then the solvent was removed by rotary evaporation. The crude product was purified on a silica column using petroleum ether as eluent to afford **4** as a colorless oily liquid (180 mg, 42%). ^{1}H NMR (500 MHz, chloroform-*d*) *δ* = 7.70 (s, 1H, C2′H), 7.48 (d, *J* = 7.4 Hz, 1H, C6′H or C4′H), 7.41 (d, *J* = 7.4 Hz, 1H, C4′H or C6′H), 7.39 (d, *J* =

8.8 Hz, 2H, C2H), 7.27 (t, J = 7.4 Hz, 1H, C5'H), 7.09 (d, J = 8.4 Hz, 4H, C7H), 7.07 (d, J = 8.8 Hz, 2H, C3H), 7.05 (d, J = 8.4 Hz, 4H, C6H), 2.34 (s, 6H, C9H_3) ppm. GC-MS (EI$^+$) *m/z*: $[M]^+$ calcd for $C_{26}H_{22}BrN$, 427; found, 427.

Synthesis of DA-*m*Benz.

DA-*m*Benz was synthesized with the same method of synthesizing **DA-*p*Benz** using **4** as starting material. **DA-*m*Benz** was obtained as yellow solids (168 mg, 67%). ^{1}H NMR (500 MHz, chloroform-*d*) δ = 7.75 (s, 1H, C2′H), 7.69 (d, J = 7.6 Hz, 1H, C6′H or C4′H), 7.46 (d, J = 7.3 Hz, 1H, C4′H or C6′H), 7.41 (t, J = 7.4 Hz, 1H, C5′H), 7.40 (d, J = 8.8 Hz, 2H, C2H), 7.09 (d, J = 8.4 Hz, 4H, C7H), 7.07 (d, J = 8.8 Hz, 2H, C3H), 7.04 (d, J = 8.4 Hz, 4H, C6H), 6.85 (s, 4H, mCH of $BMes_2$), 2.34 (s, 12H, C9H_3 + pCH_3 of $BMes_2$), 2.06 (s, 12H, oCH_3 of $BMes_2$) ppm. ^{13}C{^{1}H} NMR (126 MHz, chloroform-*d*) δ = 147.7 (C4), 146.4 (C3′), 145.4 (C5), 141.9 (iC of $BMes_2$), 141.0 (oC of $BMes_2$), 140.3 (C1′), 138.8 (pC of $BMes_2$), 134.9 (C2′), 134.4 (C4′), 134.3 (C1), 132.7 (C8), 130.3 (C6′), 130.0 (C7), 128.5 (C5′), 128.4 (mC of $BMes_2$), 127.8 (C2), 124.8 (C6), 122.8 (C3), 23.6 (oCH$_3$ of $BMes_2$), 21.4 (pCH$_3$ of $BMes_2$), 21.0 (C9) ppm. HRMS (AP-MALDI$^+$) *m/z*: $[M]^+$ calcd for $C_{44}H_{44}BN$, 597.3557; found, 597.3561 ($|\Delta|$ = 0.03 ppm).

2. Results and Discussion.

2.1 Electrochemistry.

Electrochemical experiments have been performed in the glovebox under N_2-atmosphere. THF was distilled, dried over Na/K alloy and degassed before use. DCM was distilled over CaH, degassed and stored over molecular sieves before use. Electrolyte concentrations were 0.1 M for THF and DCM. The experiments have been done in a three electrode electrochemical cell (working: 1 mm Pt-disc, counter: Pt-wire, pseudo reference: Pt-wire). All voltammograms were referenced to the Fc/Fc^+ redox couple.

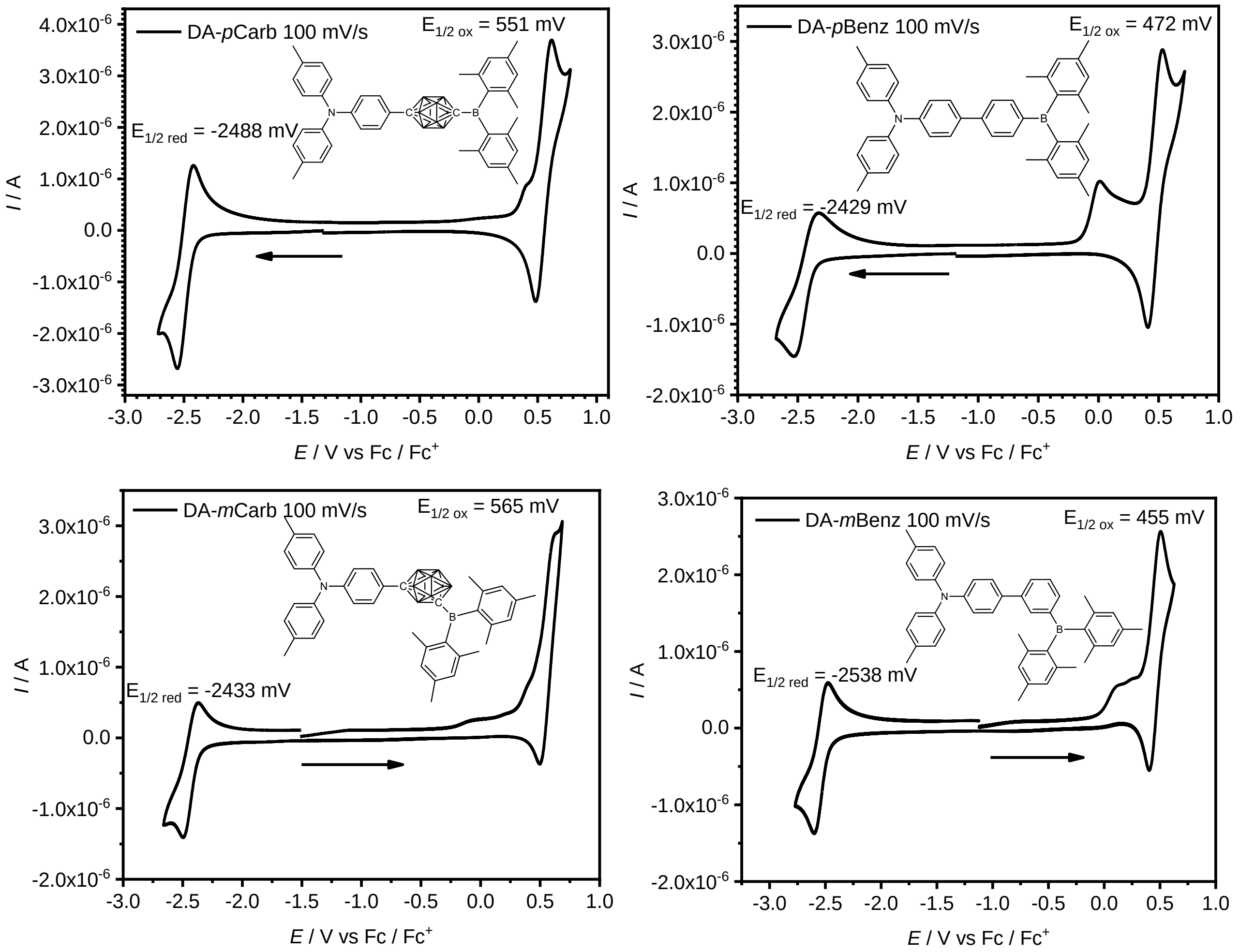

Supplementary Fig. 5. Cyclic voltammograms of **DA-*p*Carb**, **DA-*p*Benz**, **DA-*m*Carb**, and **DA-*m*Benz** in THF. There are signs of decomposition upon oxidation visible in all measurements in THF. This could not be avoided by experimental means (scan direction, polishing of working electrode) and is most likely due to known decomposition in polar solvents, for comparison measurements in DCM were done and shown below.

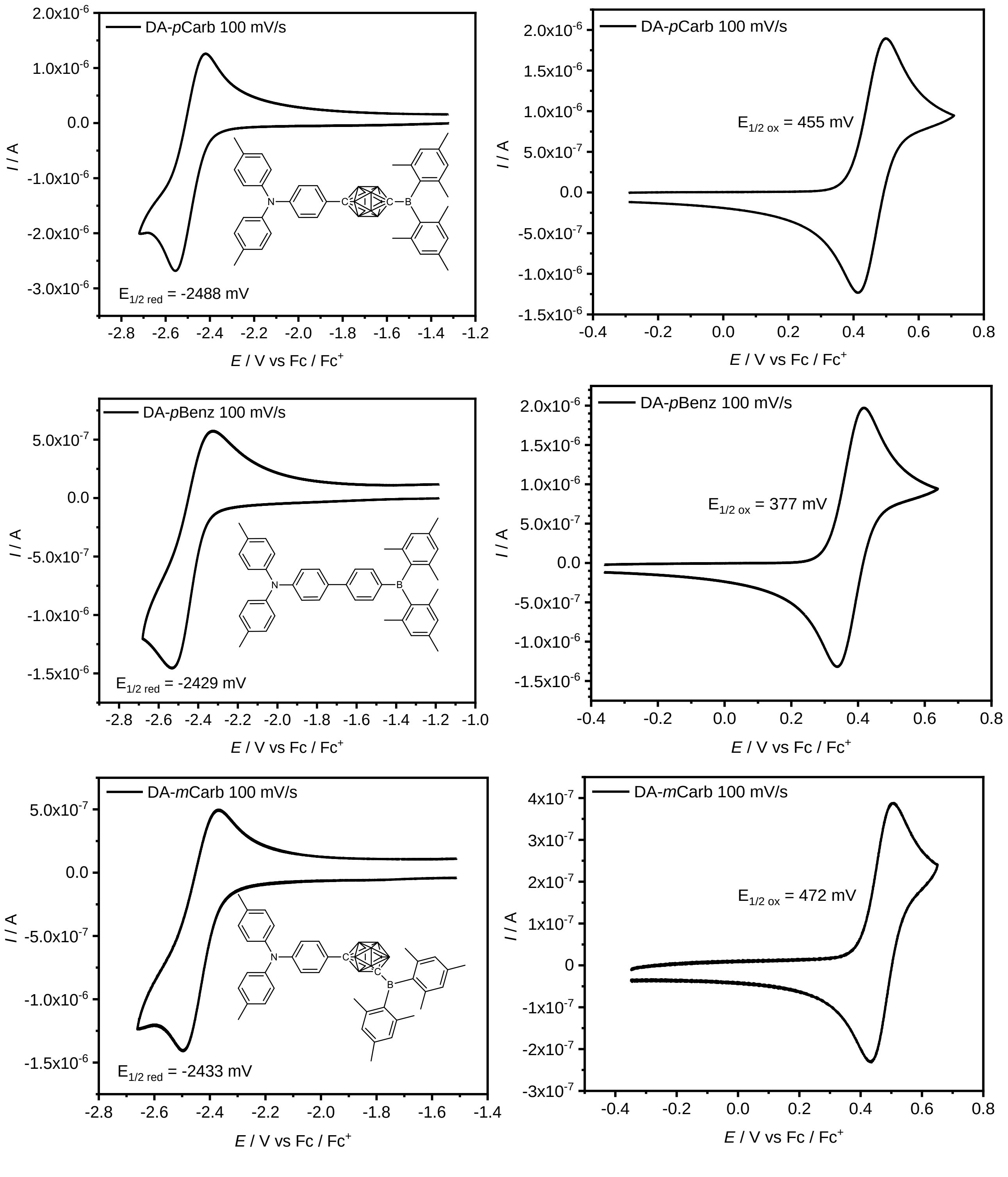
DA-pCarb 100 mV/s
E1/2 red = -2488 mV
E1/2 ox = 455 mV
DA-pBenz 100 mV/s
E1/2 red = -2429 mV
E1/2 ox = 377 mV
DA-mCarb 100 mV/s
E1/2 red = -2433 mV
E1/2 ox = 472 mV
I / A
E / V vs Fc / Fc+
N
C
B

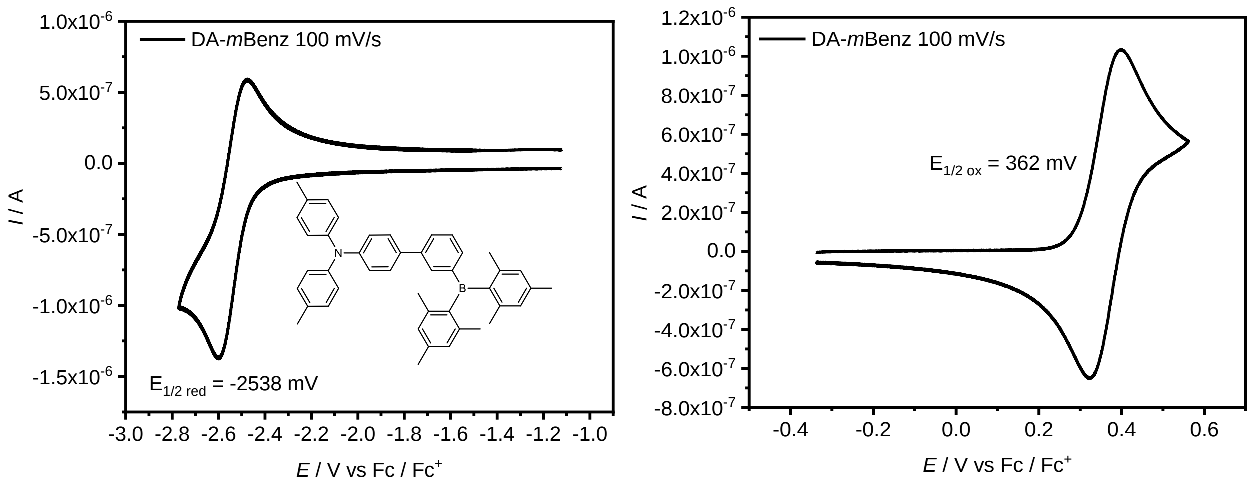

Supplementary Fig. 6. Cyclic voltammograms of **DA-*p*Carb**, **DA-*p*Benz**, **DA-*m*Carb** and **DA-*m*Benz** in THF (reduction) and DCM (oxidation). Here, no signs of decomposition were visible but due to the limited range of the electrochemical window, it was not possible to record the reduction waves in DCM.

2.2 Solution-state photophysics.

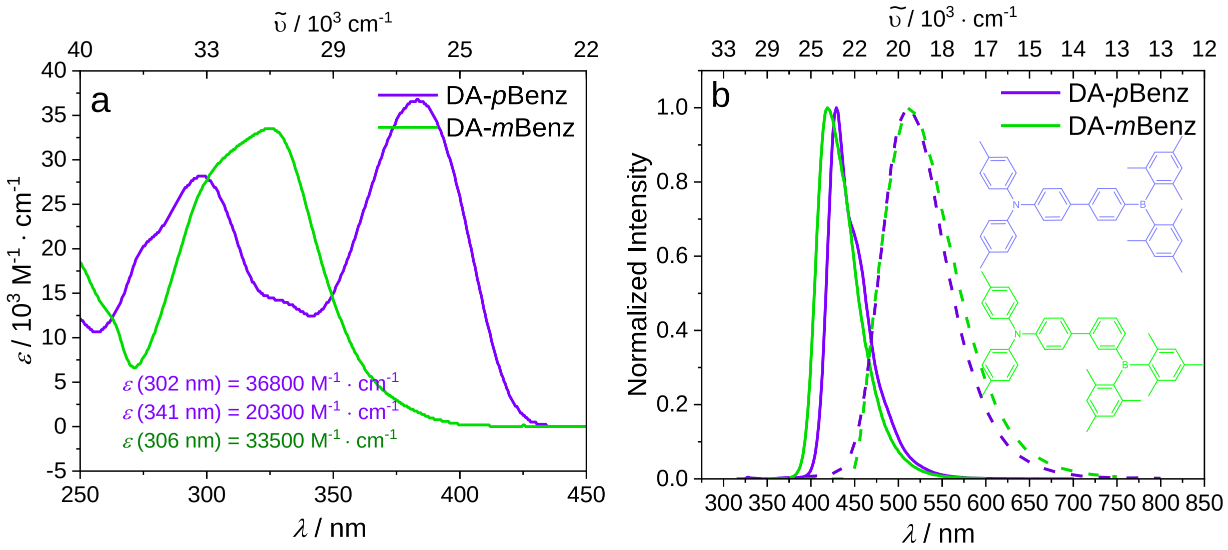

Supplementary Fig. 7. Absorption (a) and emission (b) of **DA-*p*Benz** and **DA-*m*Benz**. Absorption and normalized emission spectra of **DA-*p*Benz** (excited at 337 nm, purple) and **DA-*m*Benz** (excited at 310 nm, blue) in hexane (solid line) and THF (dashed line) at room temperature.

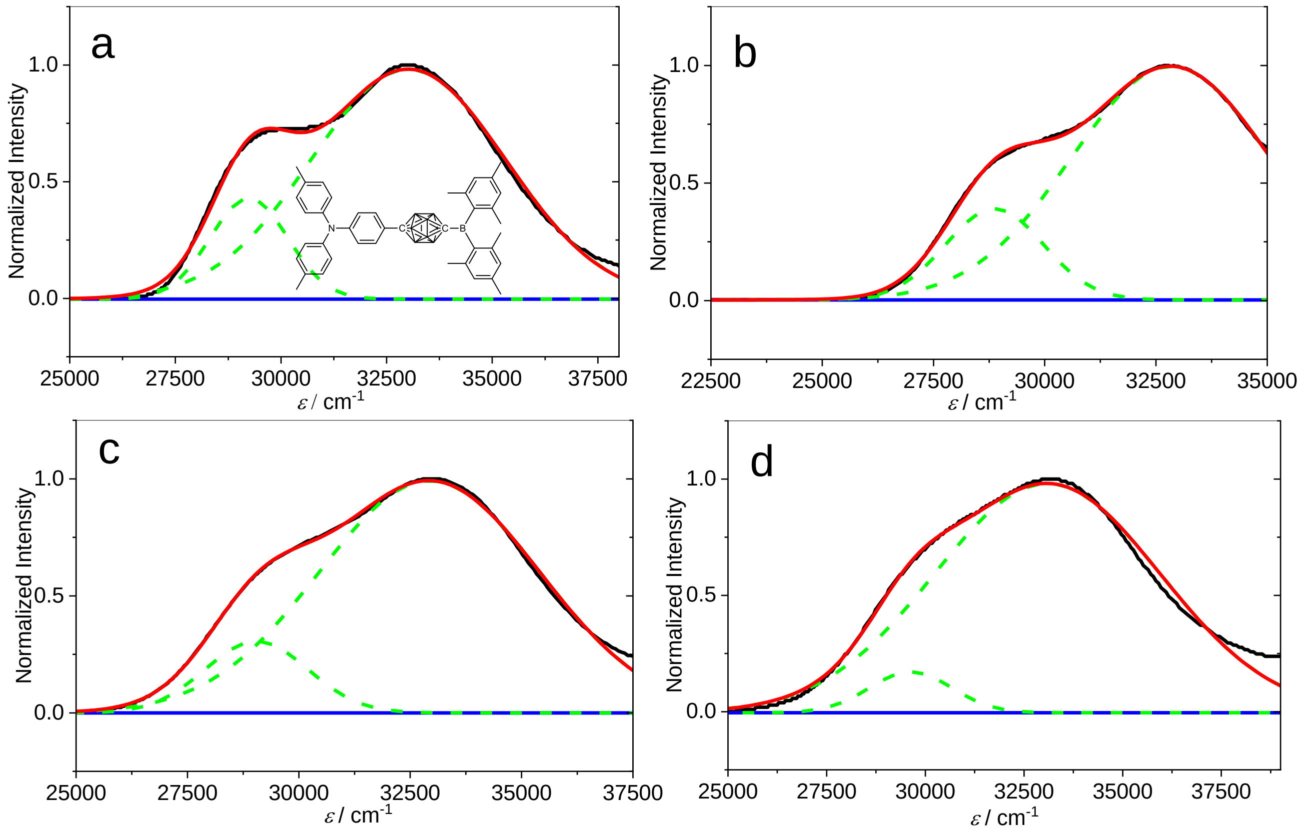

Supplementary Fig. 8. Gaussian fitted absorption spectra. The UV-vis spectra of **DA-*p*Carb** in different solvents (a. hexane, b. toluene, c. THF, d. MeCN) deconvolution by Gaussian functions

(green dots), the red dash-dot lines are the sum spectra which matched the measured spectra (black line) (coefficient of determination R^2=0.997, 0.999, 0.999, 0.994, respectively). Two Gaussian functions were used for the deconvolution of the asymmetric band.

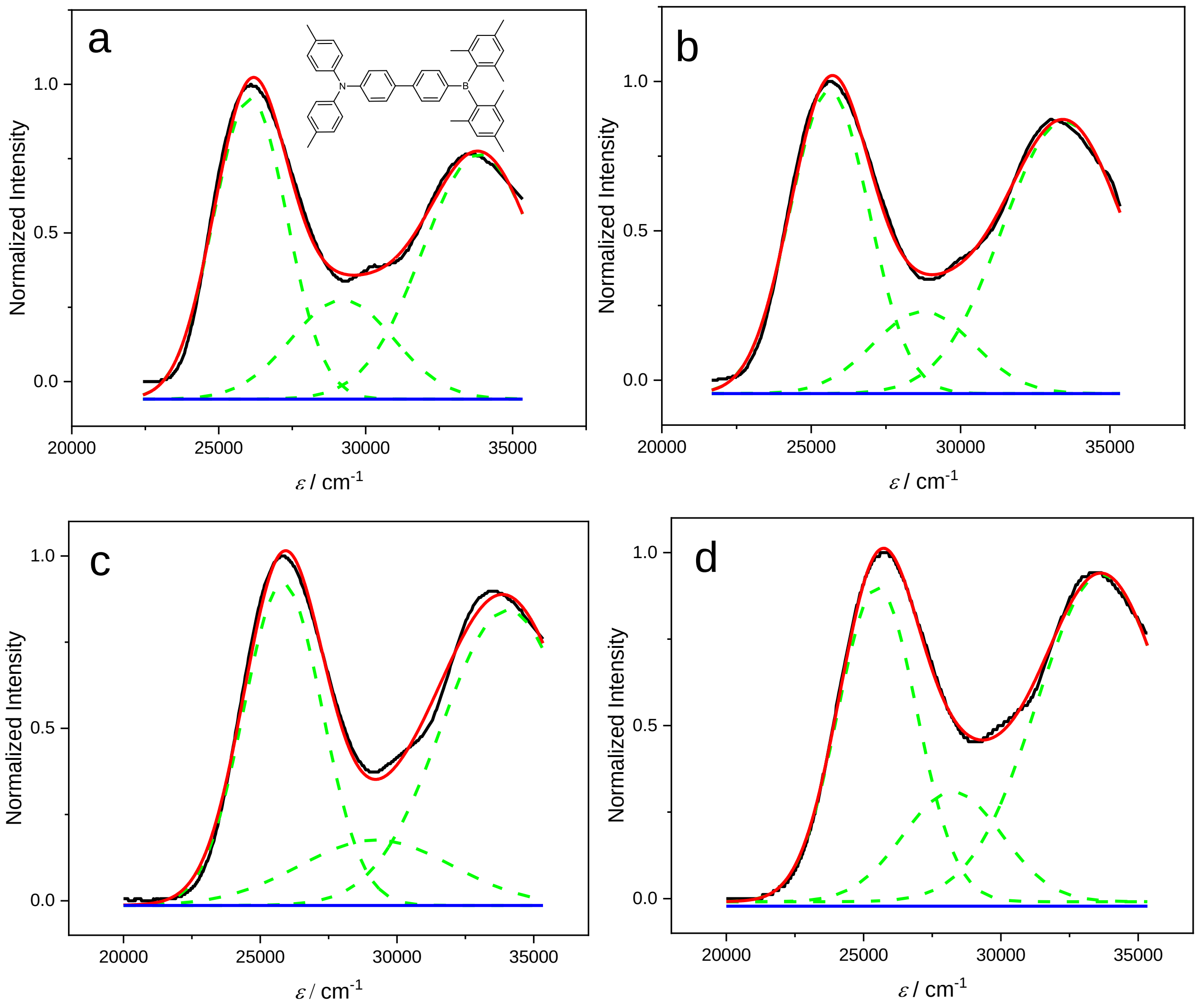

Supplementary Fig. 9. Gaussian fitted absorption spectra. The UV-vis spectra of **DA-*p*Benz** in different solvents (a. hexane, b. toluene, c. THF, d. MeCN). Deconvolution by Gaussian functions (green lines), and the red dash-dot lines are the sum spectra which matched the measured spectra (black line) (coefficient of determination R^2=0.994, 0.996, 0.996, 0.999, respectively). Three Gaussian functions were used for the deconvolution of the asymmetric band.

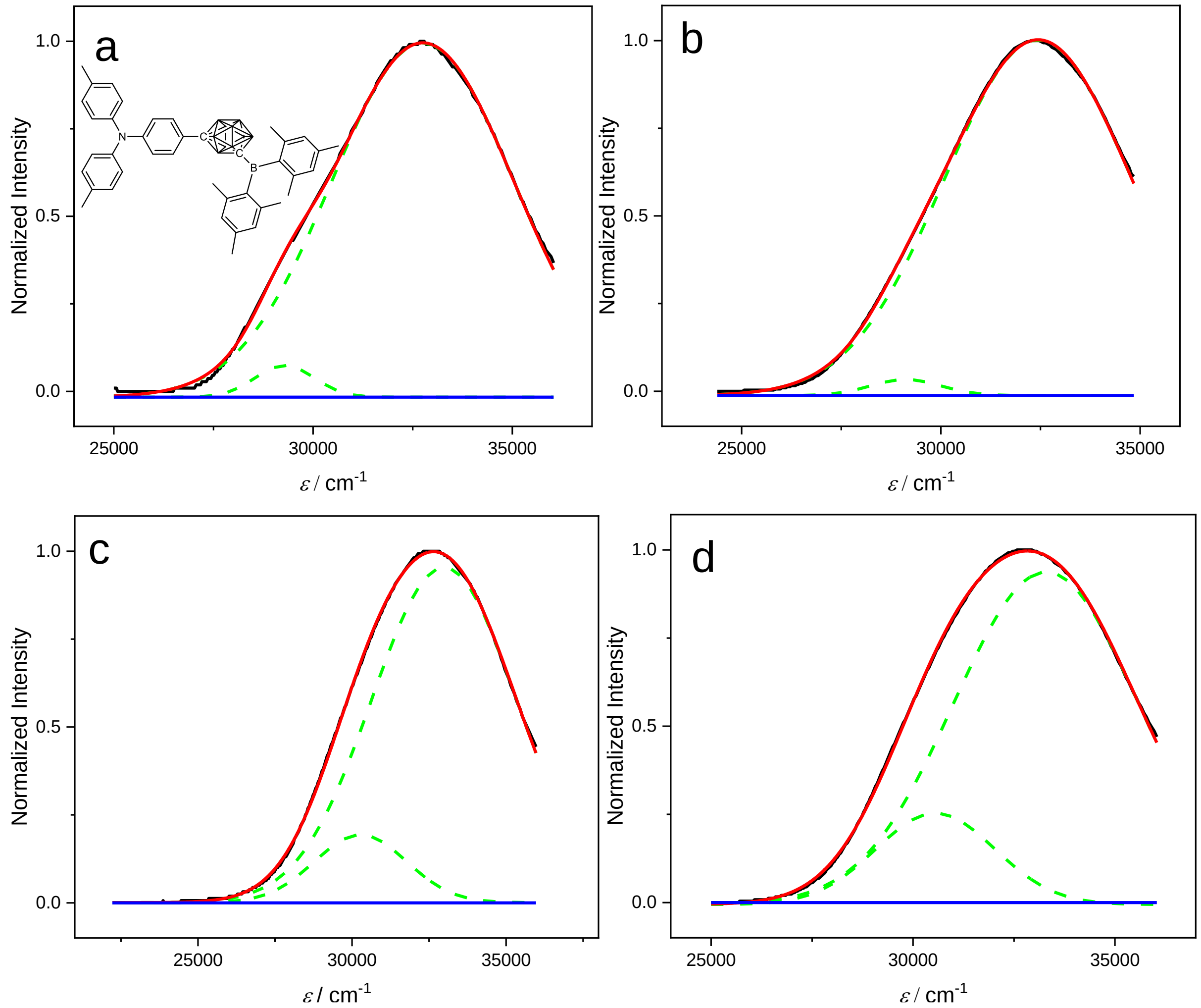

Supplementary Fig. 10. Gaussian fitted absorption spectra. The UV-vis spectra of **DA-*m*Carb** in (a. hexane, b. toluene, c. THF, d. MeCN), deconvolution by Gaussian functions (green dots), and the red dash-dot lines are the sum spectra which matched the measured spectra (black line) (coefficient of determination R^2=0.999, 0.999, 0.999, 0.999). Two Gaussian functions were used for the deconvolution of the asymmetric band.

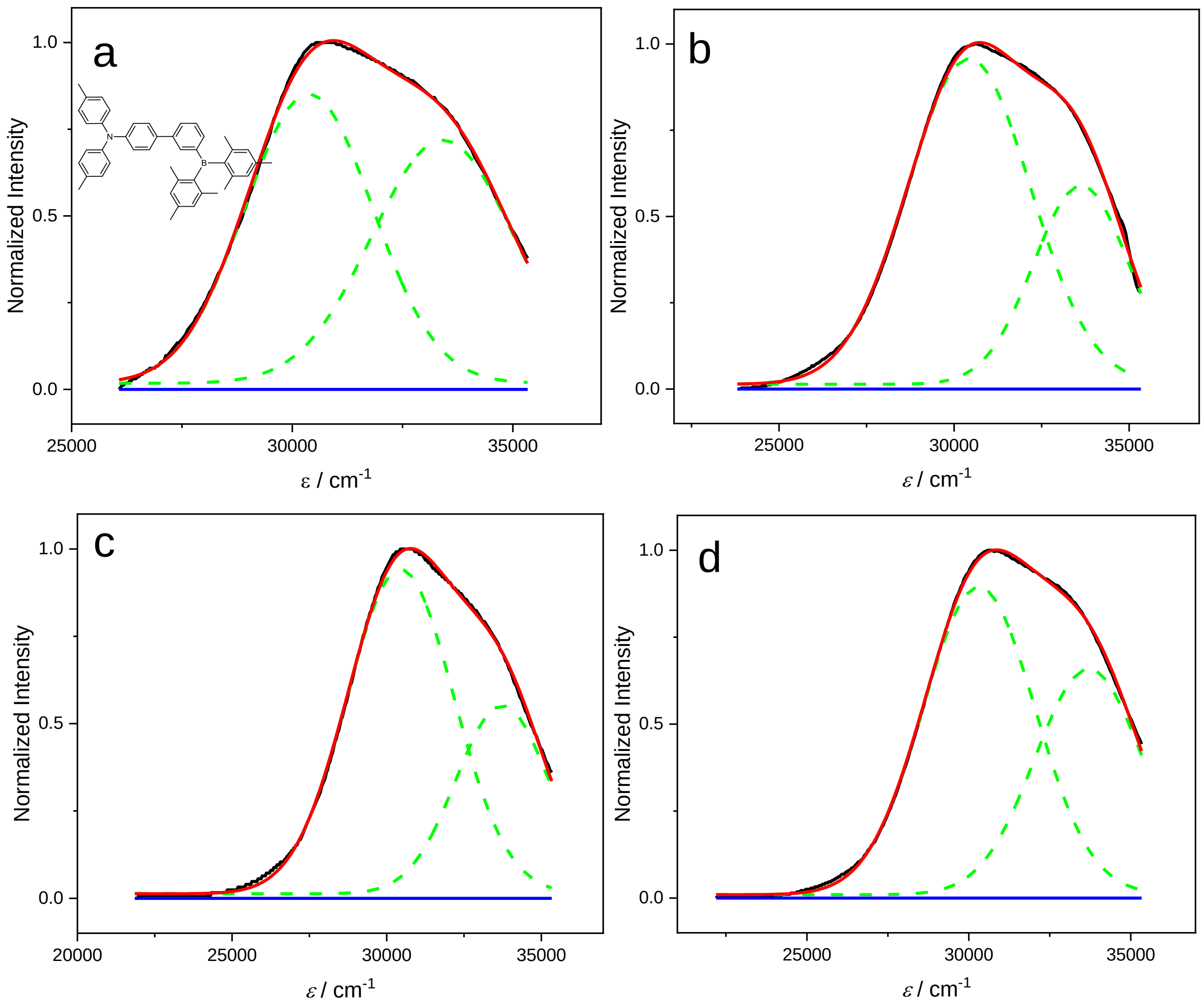

Supplementary Fig. 11. Gaussian fitted absorption spectra. The UV-vis spectra of **DA-*m*Benz** in different solvents (a. hexane, b. toluene, c. THF, d. MeCN). Deconvolution by Gaussian functions (green lines), and the red dash-dot lines are the sum spectra which matched the measured spectra (black line) (coefficient of determination R^2=0.999, 0.999, 0.999, 0.999, respectively). Two Gaussian functions were used for the deconvolution of the asymmetric band.

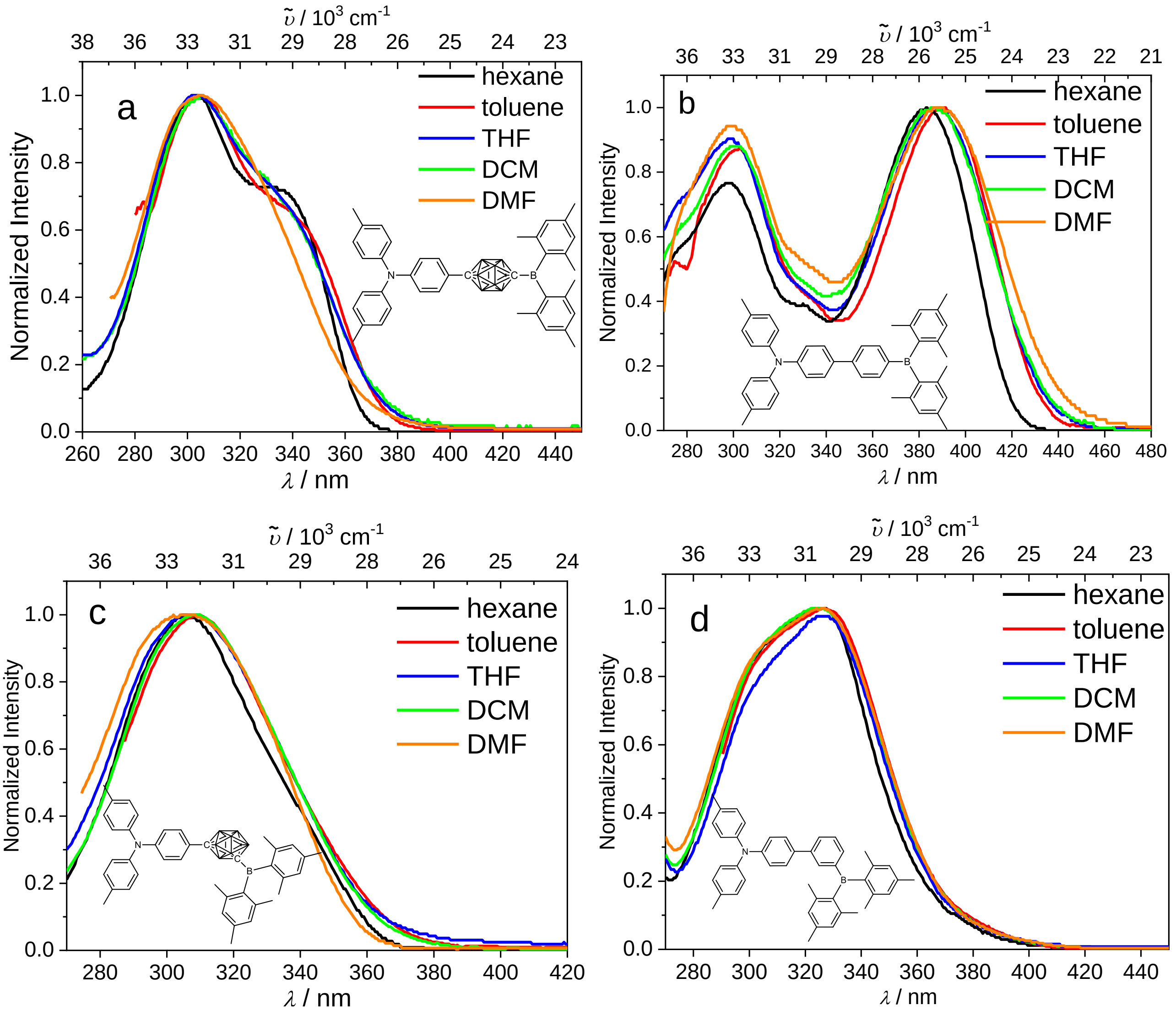

Supplementary Fig. 12. Absorption spectra. Normalized absorption spectra of **DA-*p*Carb** (a), **DA-*p*Benz** (b), **DA-*m*Carb** (c) and **DA-*m*Benz** (d) in different solvents. Black, red, bule, green and orange lines represent hexane, toluene, THF, DCM and DMF, respectively.

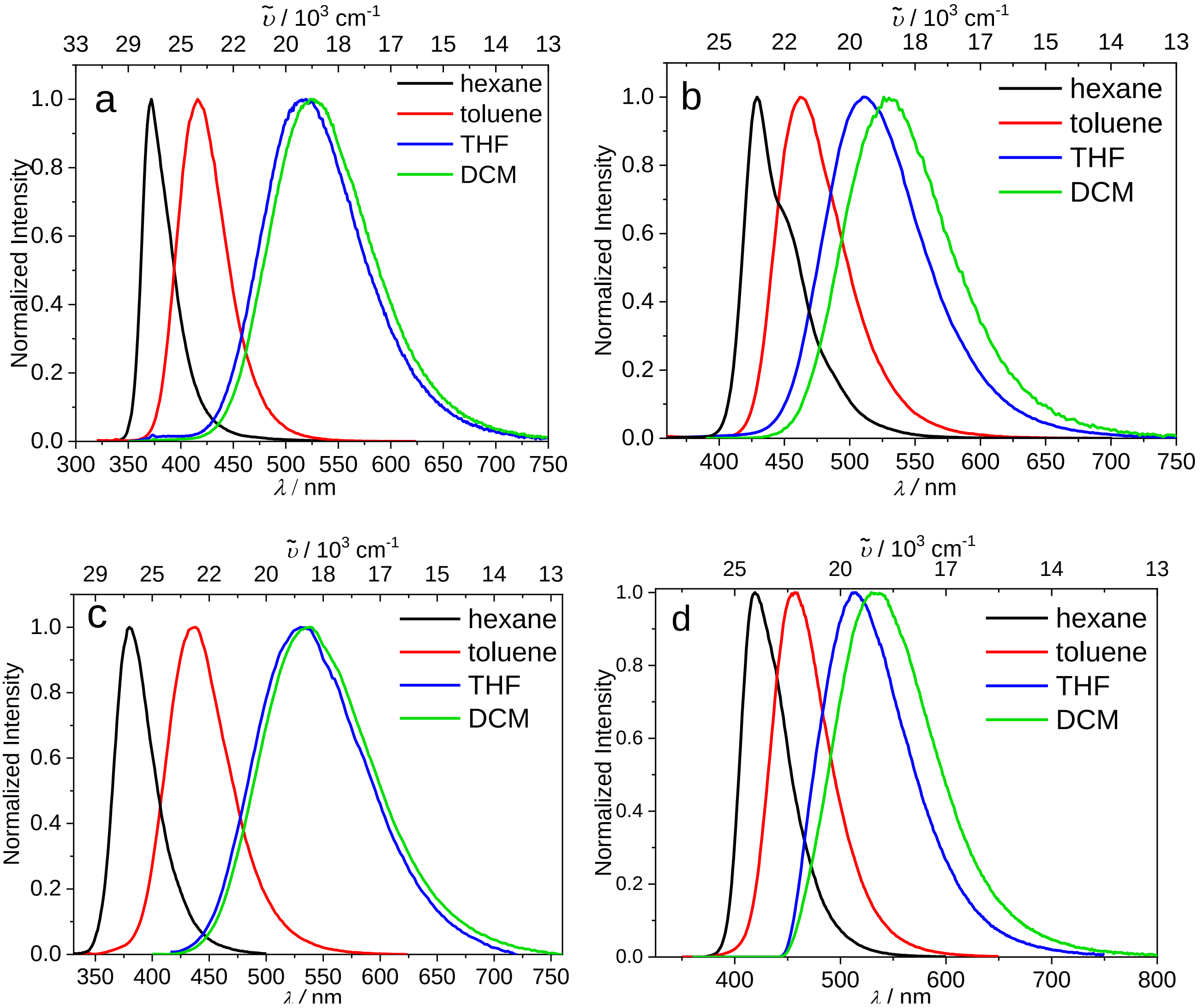

Supplementary Fig. 13. Emission spectra. Normalized emission spectra of **DA-*p*Carb** (a), **DA-*p*Benz** (b), **DA-*m*Carb** (c) and **DA-*m*Benz** (d) in different solvents. Black, red, bule, and green lines represent hexane, toluene, THF, and DCM, respectively.

Supplementary Tab. 1. Determined 00-energy for **DA-*p*Carb**, **DA-*p*Benz**, **DA-*m*Carb** and **DA-*m*Benz** in hexane and THF.

	**DA-*p*Carb**	**DA-*p*Benz**	**DA-*m*Carb**	**DA-*m*Benz**
Hexane	28100 cm^{-1}	24500 cm^{-1}	28100 cm^{-1}	25400 cm^{-1}
THF	23000 cm^{-1}	22200 cm^{-1}	22300 cm^{-1}	22100 cm^{-1}

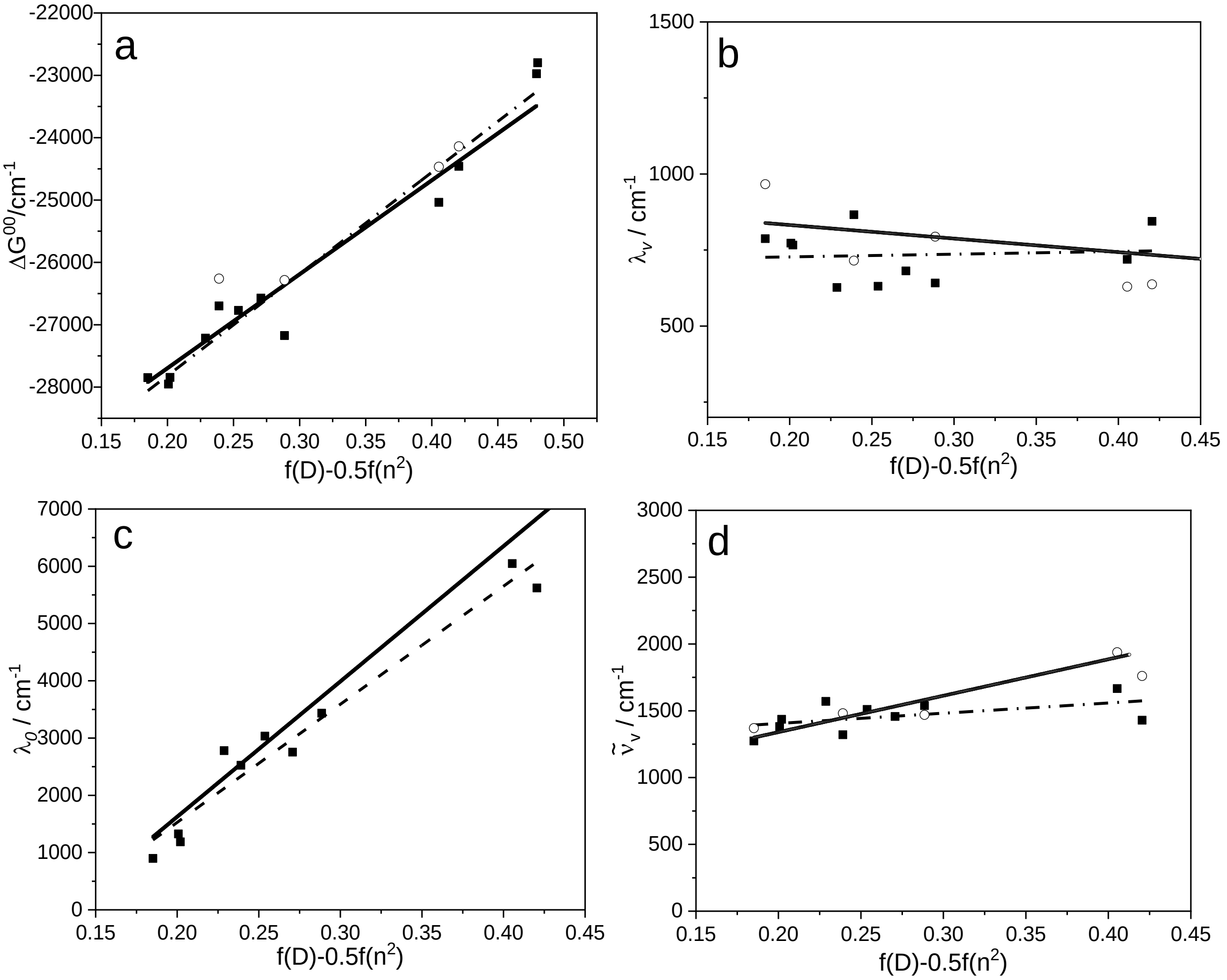

Supplementary Fig. 14. Plots of (a) ΔG^{00}, (b) λ_v, (c) λ_0, and (d) $\tilde{\upsilon}_v$ versus the solvent polarity function for **DA-*p*Carb** (dash line), and **DA-*m*Carb** (solid line). ΔG^{00} is the difference in the free energy between the diabatic ground and the excited state. λ_v and λ_0 are the inner reorganization energy and outer reorganization energy. $\tilde{\upsilon}_v$ is the average molecular vibrational mode (see Theoretical background).

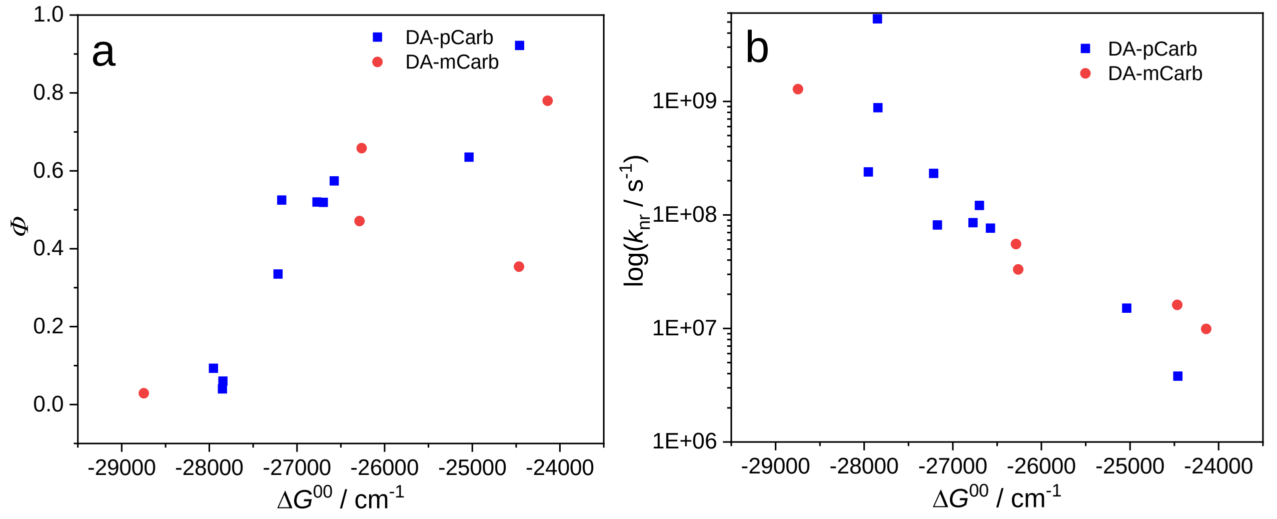

Supplementary Fig. 15. Plots of quantum yield versus ΔG^{00} (a), and the trend of logk_{nr} versus ΔG^{00} (b). Bule and red square represent **DA-*p*Carb** and **DA-*m*Carb**, respectively.

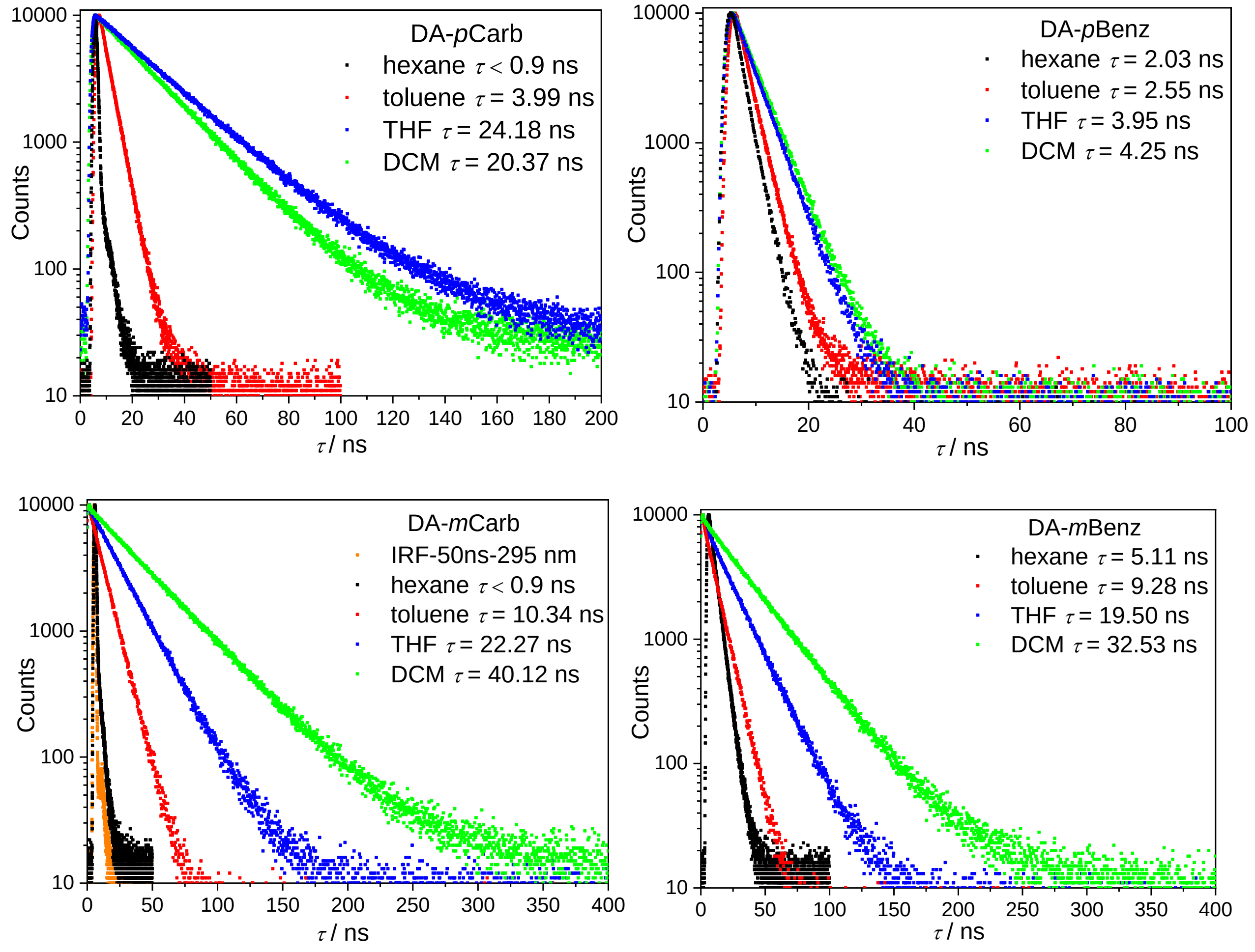

Supplementary Fig. 16. Fluorescence decay profiles of **DA-*p*Carb**, **DA-*p*Benz**, **DA-*m*Carb** and **DA-*m*Benz** measured by TCSPC in different solvents. Black, red, green, bule dots represent hexane, toluene, THF and DCM, respectively.

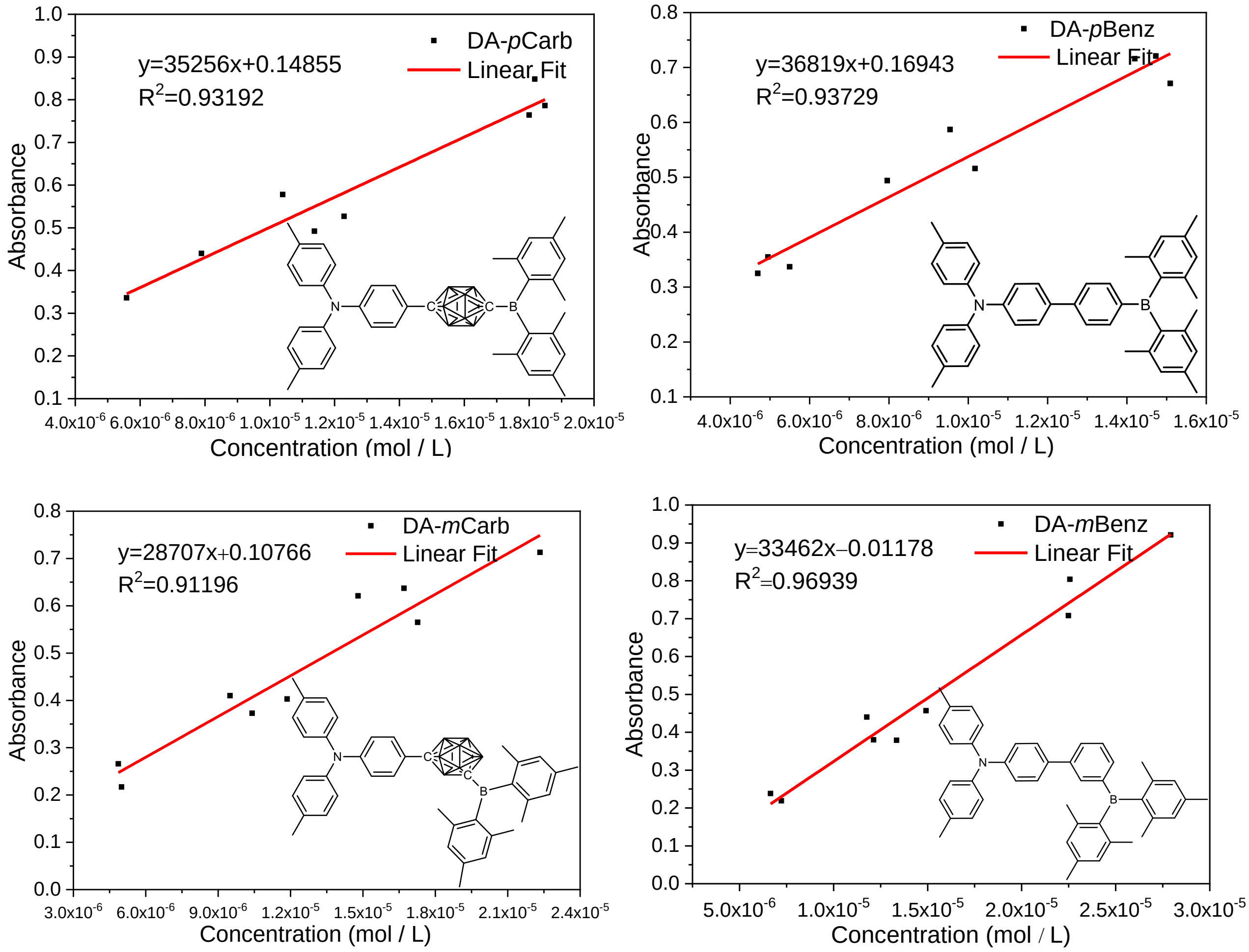

Supplementary Fig. 17. Calculation of molar extinction coefficient. Absorbance of **DA-*p*Carb** at 306 nm, **DA-*p*Benz** at 383 nm, **DA-*m*Carb** at 303 nm, and **DA-*m*Benz** at 325 nm in hexane at different concentrations. The slope of the fitted line (in red) is the molar extinction coefficient of the corresponding compound.

Supplementary Tab. 2. The main parameters of the Lippert-Mataga equation of **DA-*p*Carb**.

**DA-*p*Carb**	ε	n	Δf	Stokes shift / cm^{-1}
hexane	1.882	1.375	0.133	2360
cyclohexane	2.017	1.427	0.131	2400
dodecane	2.006	1.421	0.132	2500
mesitylene	2.265	1.499	0.131	4300
toluene	2.374	1.494	0.141	4800
ortho-xylene	2.545	1.505	0.149	5000
tetralin	2.771	1.541	0.151	4830
dibutylether	3.047	1.399	0.207	4900
THF	7.426	1.407	0.266	9800
DCM	8.930	1.424	0.267	9900
DMF	37.219	1.431	0.288	15100
MeCN	35.688	1.344	0.318	15100

Supplementary Tab. 3. The main parameters of the Lippert-Mataga equation of **DA-*p*Benz**.

**DA-*p*Benz**	ε	n	Δf	Stokes shift / cm^{-1}
hexane	1.882	1.375	0.133	3000
toluene	2.374	1.494	0.141	6300
dibutylether	3.047	1.399	0.207	7000
THF	7.426	1.407	0.266	9900
DCM	8.930	1.424	0.267	10500

Supplementary Tab. 4. The main parameters of the Lippert-Mataga equation of **DA-*m*Carb**.

**DA-*m*Carb**	ε	n	Δf	Stokes shift / cm^{-1}
hexane	1.882	1.375	0.133	2900
toluene	2.374	1.494	0.141	6500
THF	7.426	1.407	0.266	10300
DCM	8.930	1.424	0.267	10500
MeCN	35.688	1.344	0.318	13700

Supplementary Tab. 5. The main parameters of the Lippert-Mataga equation of **DA-*m*Benz**.

**DA-*m*Benz**	ε	n	Δf	Stokes shift / cm^{-1}
hexane	1.882	1.375	0.133	6800
toluene	2.374	1.494	0.141	8700
THF	7.426	1.407	0.266	11000
DCM	8.930	1.424	0.267	11900
MeCN	35.688	1.344	0.318	13800

Supplementary Tab. 6. Photophysical properties of **DA-*p*Carb** and **DA-*m*Carb** in various solvents.

		ϕ	τ / ns	k_r / $10^7 s^{-1}$	k_{nr} / $10^7 s^{-1}$	μ^2_{abs} / D^2	μ^2_{fl} / D^2	V(MH) / cm^{-1}
**DA-*p*Carb**	hexane	0.04	0.18	22.22	533.33	14.7	17.5	2440
	cyclohexane	0.06	1.07	5.61	87.85	15	4.03	1160
	dodecane	0.09	3.79	2.45	23.93	15.2	1.8	773
	mesitylene	0.34	2.87	11.67	23.17	15.8	9.09	1620
	toluene	0.52	3.99	13.01	12.06	14.2	10.9	1730
	ortho-xylene	0.52	5.63	9.24	8.53	16.2	7.74	1440
	tetralin	0.57	5.57	10.31	7.65	15.8	8.01	1470
	dibutylether	0.53	5.83	9.01	8.15	17.3	9.46	1600
	THF	0.64	24.18	2.63	1.51	19.6	5.35	967
	DCM	0.92	20.37	4.53	0.38	15.2	9.32	1250
**DA-*m*Carb**	hexane	0.03	0.76	3.82	127.76	0.76	3.2	1010
	toluene	0.66	10.34	6.36	3.31	0.97	6.15	1240
	dibutylether	0.47	9.567	4.92	5.53	1.54	5.9	1210
	THF	0.35	40.12	0.88	1.61	1.15	1.96	571
	DCM	0.78	22.27	3.50	0.99	0.91	7.66	1110

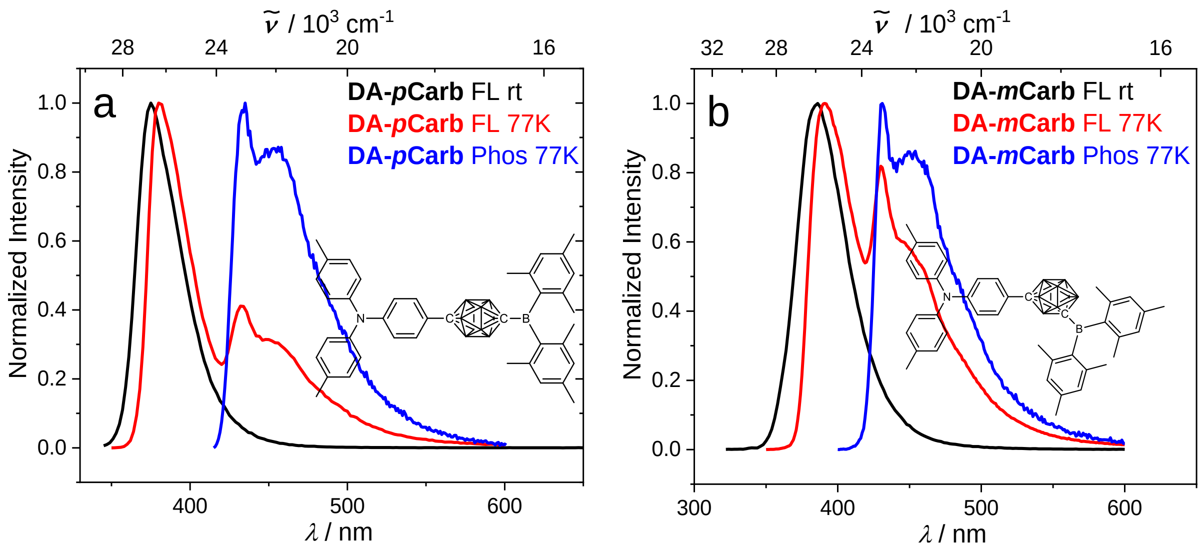

Supplementary Fig. 18. Fluorescence and phosphorescence (20 ms delay) spectra of (a) **DA-*p*Carb** and (b) **DA-*m*Carb** in methylcyclohexane at 77 K. Black lines represent fluorescence of **DA-*p*Carb** and **DA-*m*Carb** in room temperature, red lines represent fluorescence of **DA-*p*Carb** and **DA-*m*Carb** in 77 K, and blue lines represent phosphorescence of **DA-*p*Carb** and **DA-*m*Carb** in 77 K, respectively.

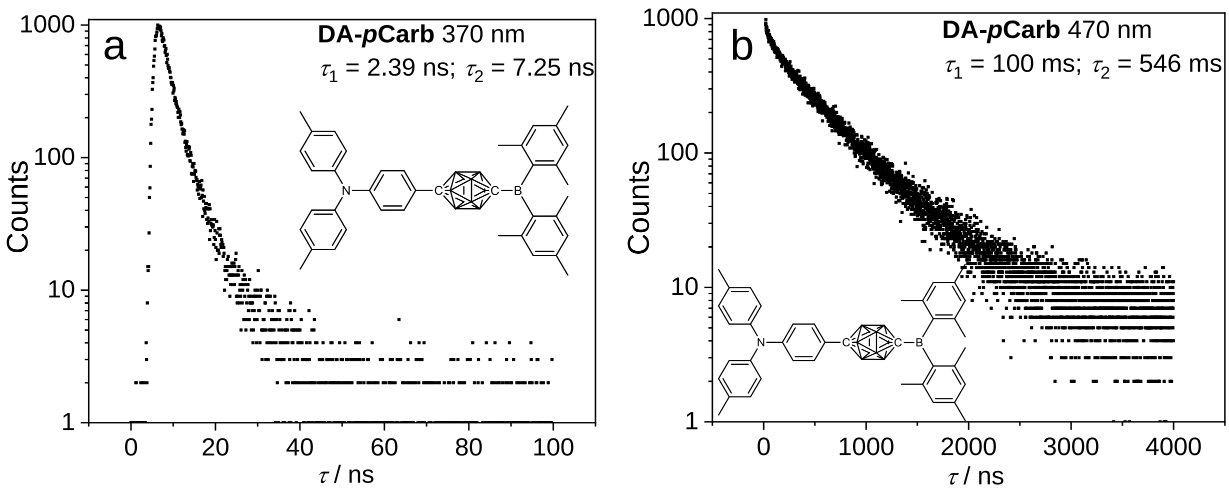

Supplementary Fig. 19. Lifetimes of **DA-*p*Carb** at 370 nm (a) and 470 nm (b) (20ms gated) in methylcyclohexane at 77 K.

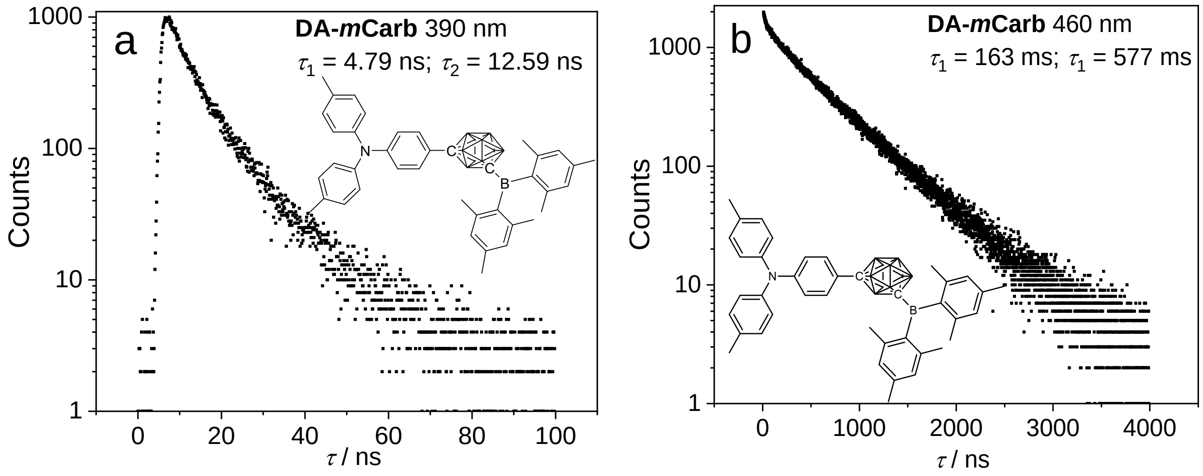

Supplementary Fig. 20. Lifetimes of **DA-*m*Carb** at 390 nm (a) and 460 nm (b) (20ms gated) in methylcyclohexane at 77 K.

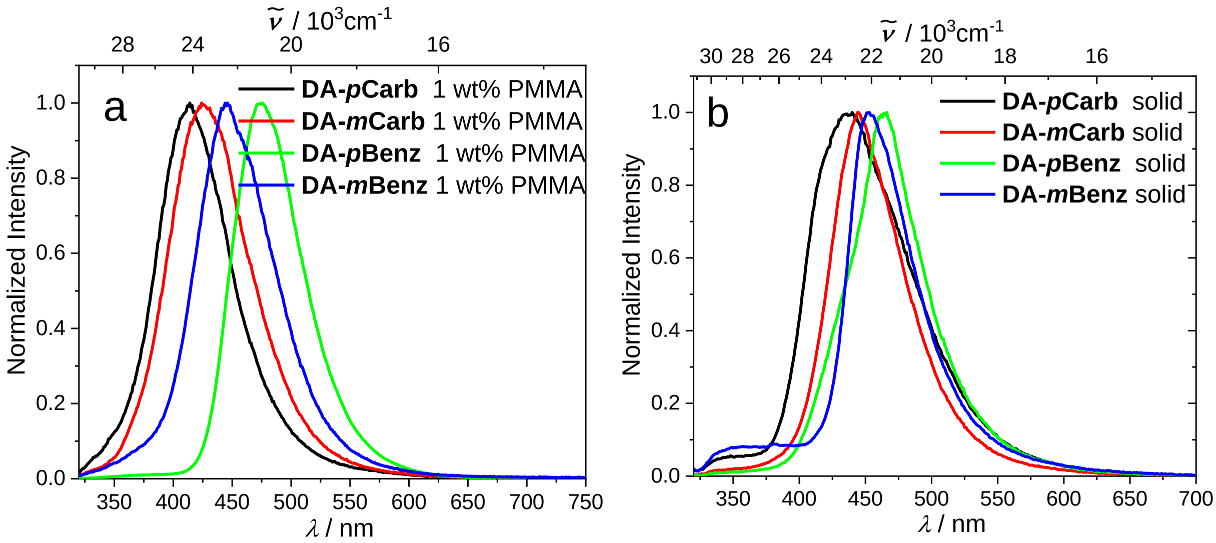

Supplementary Fig. 21. The emission spectra of **DA-*p*Carb** (black), **DA-*m*Carb** (red), **DA-*p*Benz** (green), and **DA-*m*Benz** (blue) in PMMA (1wt%) (a) and solid state (b).

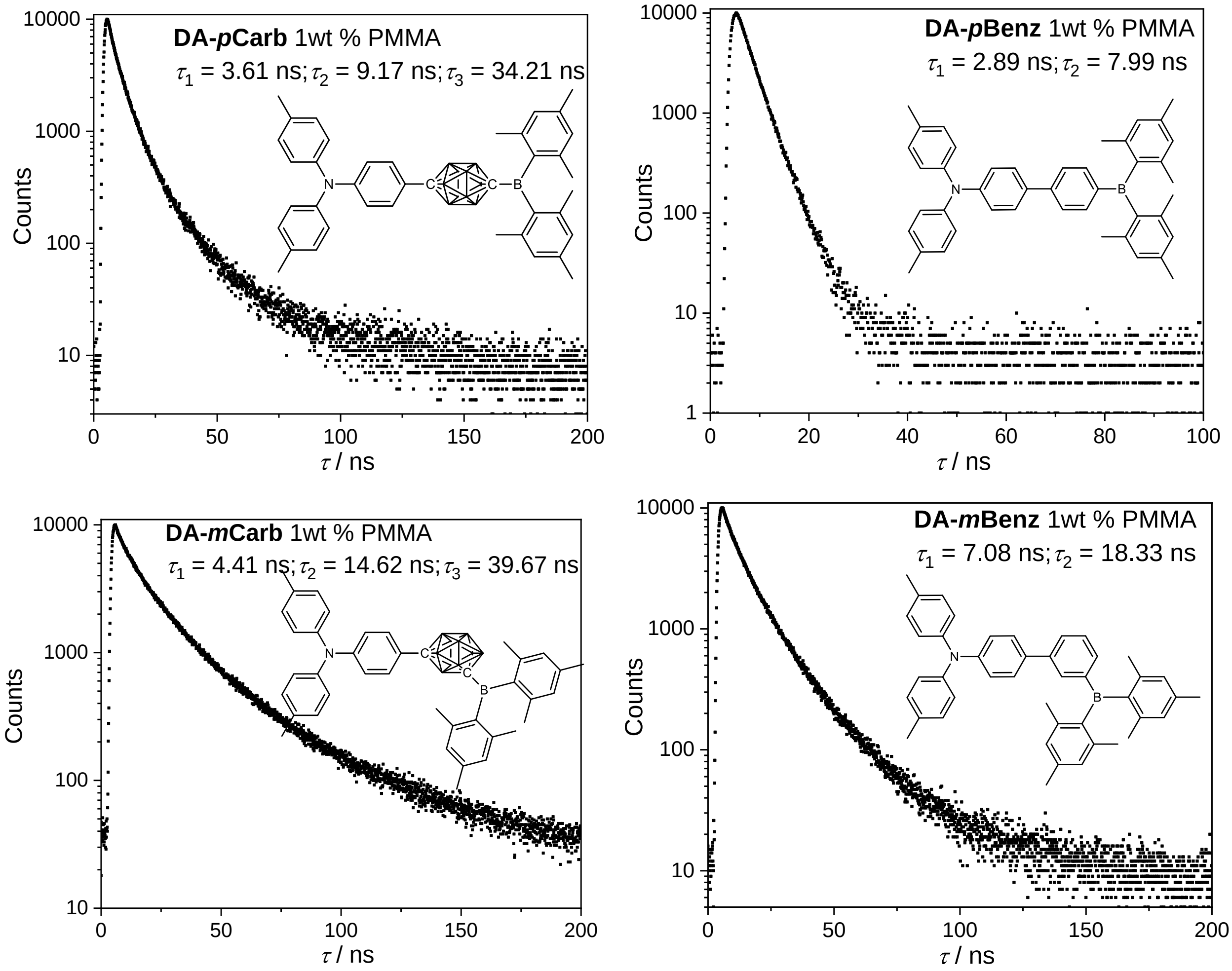

Supplementary Fig. 22. Lifetime decays of **DA-*p*Carb**, **DA-*p*Benz**, **DA-*m*Carb** and **DA-*m*Benz** in PMMA (1wt%).

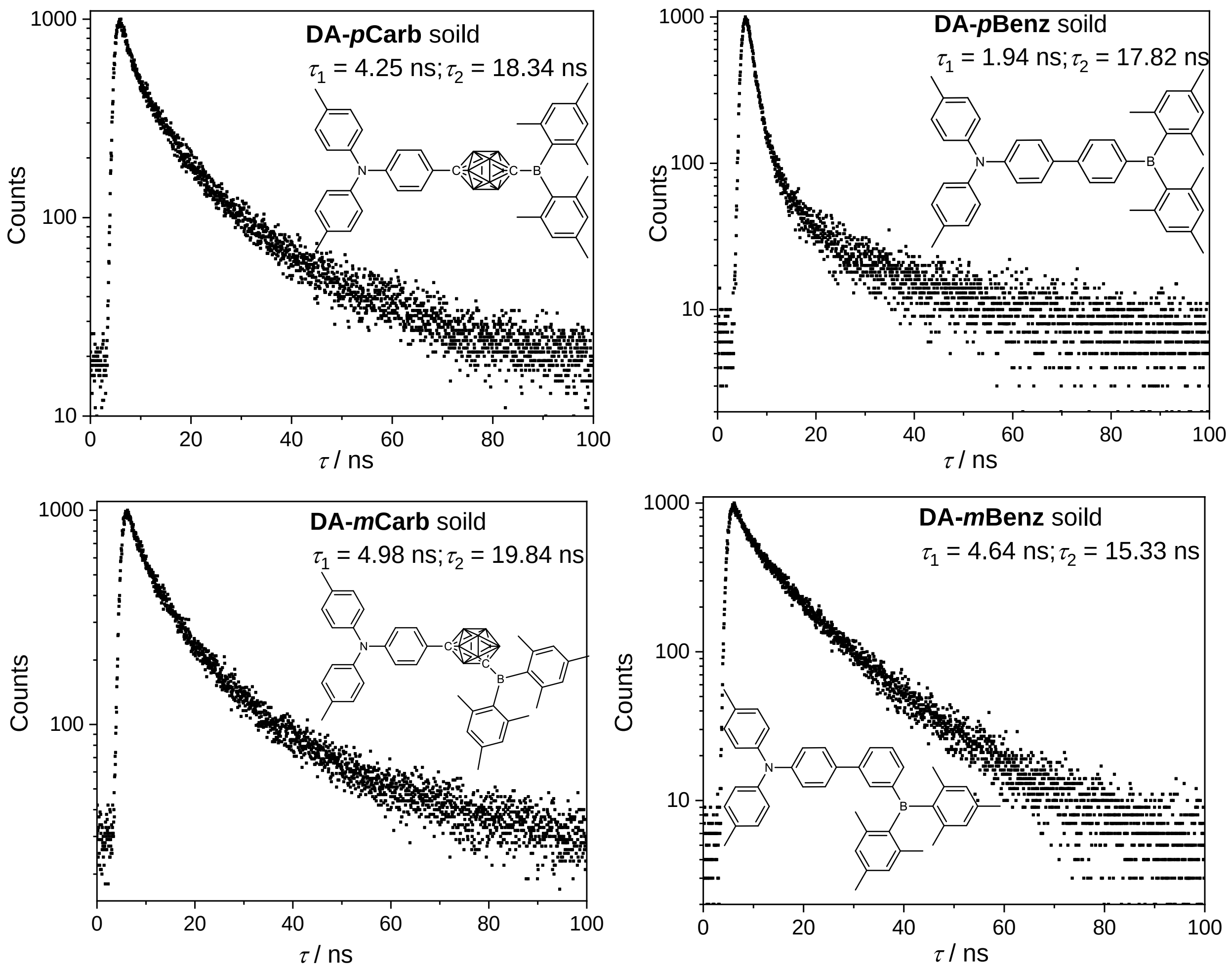

Supplementary Fig. 23. Lifetime decays of **DA-*p*Carb**, **DA-*p*Benz**, **DA-*m*Carb** and **DA-*m*Benz** in solid state.

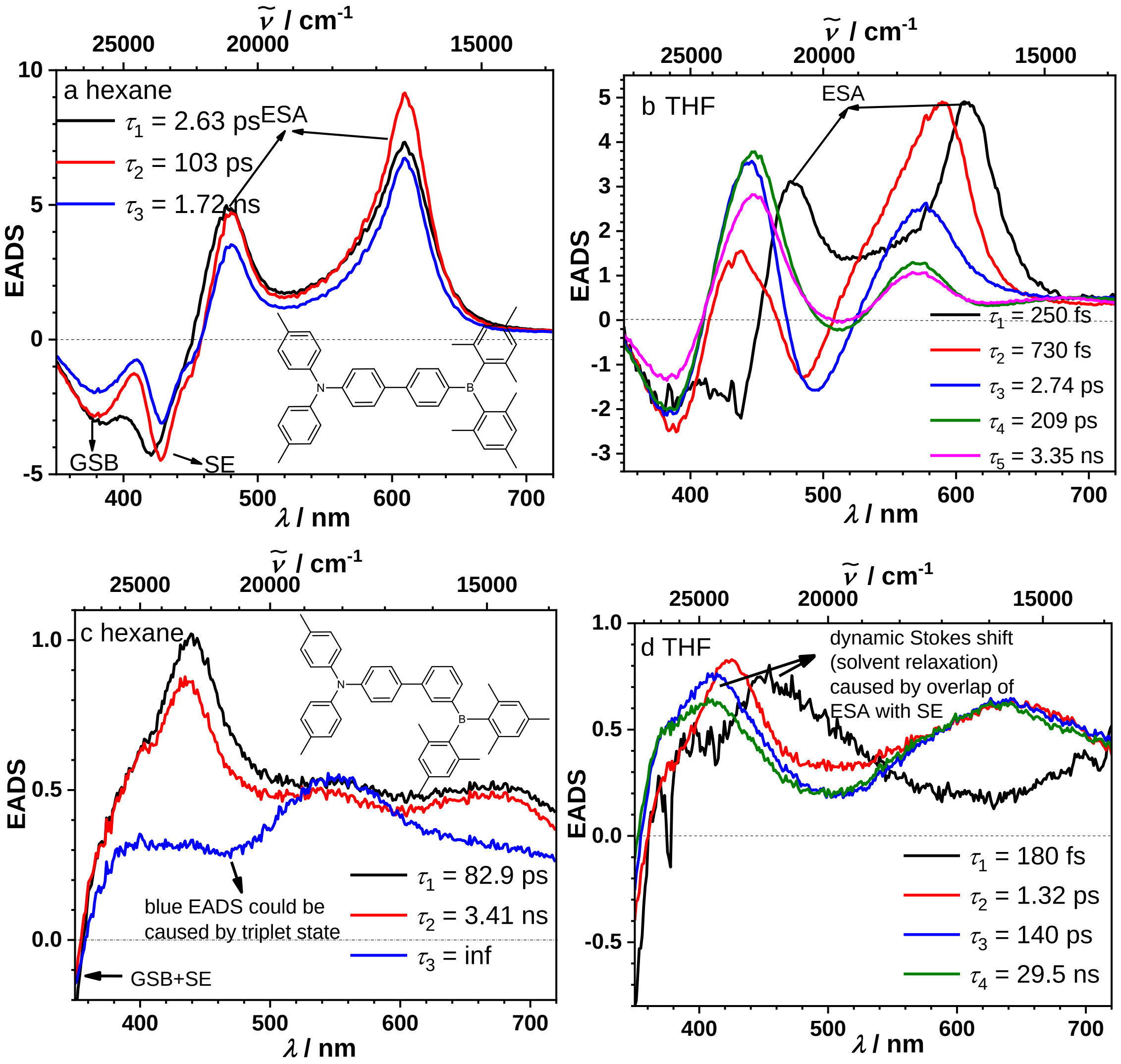

Supplementary Fig. 24. Transient absorption spectra. Evolution associated difference spectra (EADS) and lifetimes from a global deconvolution of the transient absorption spectra of **DA-*p*Benz** (a hexane, b THF) and **DA-*m*Benz** (c hexane, d THF), excited at 388 nm and 340 nm, respectively.

2.3 Theoretical studies.

2.3.1. Optimization at B3LYP/6-31G*.

	HOMO	LUMO
Top view	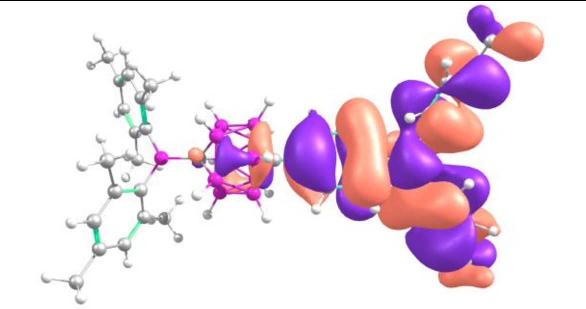	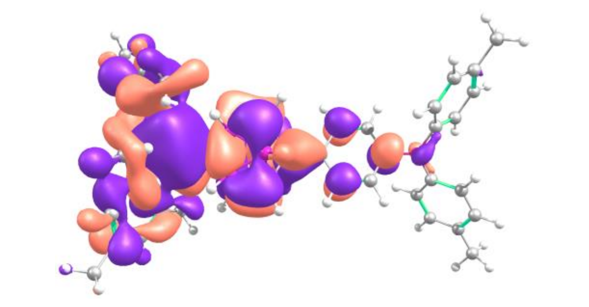
Side view	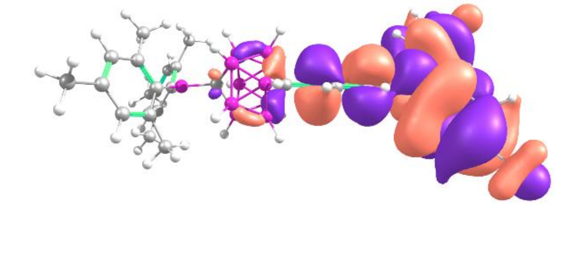	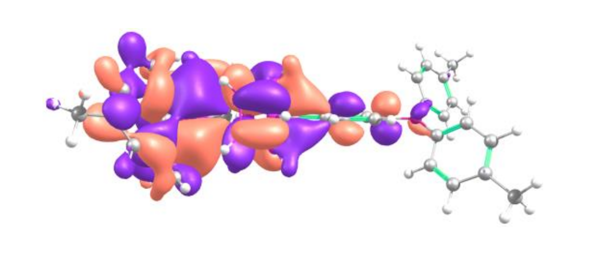
	HOMO-1	LUMO+1
Top view	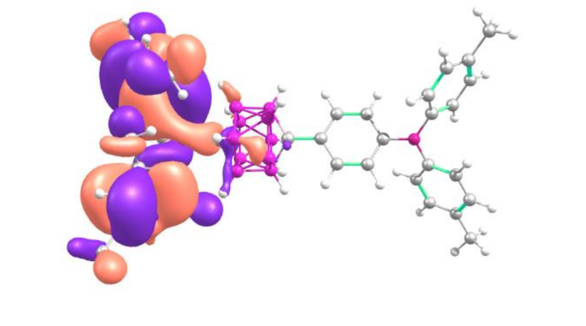	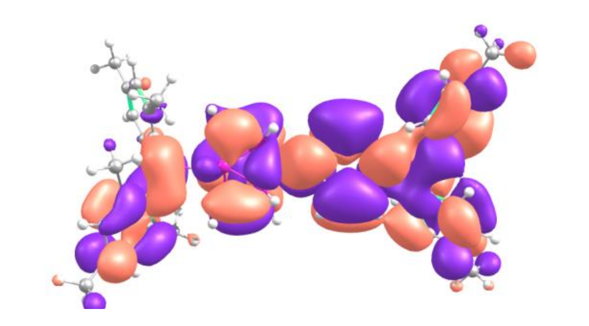
Side view	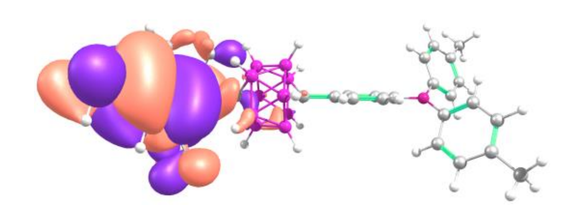	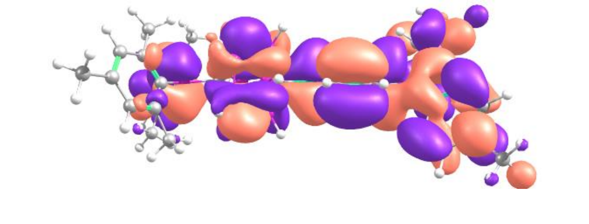

Supplementary Fig. 25. Frontier molecular orbitals of **DA-*p*Carb** in side and top view. Purple and orange indicate the different signs of the wave function.

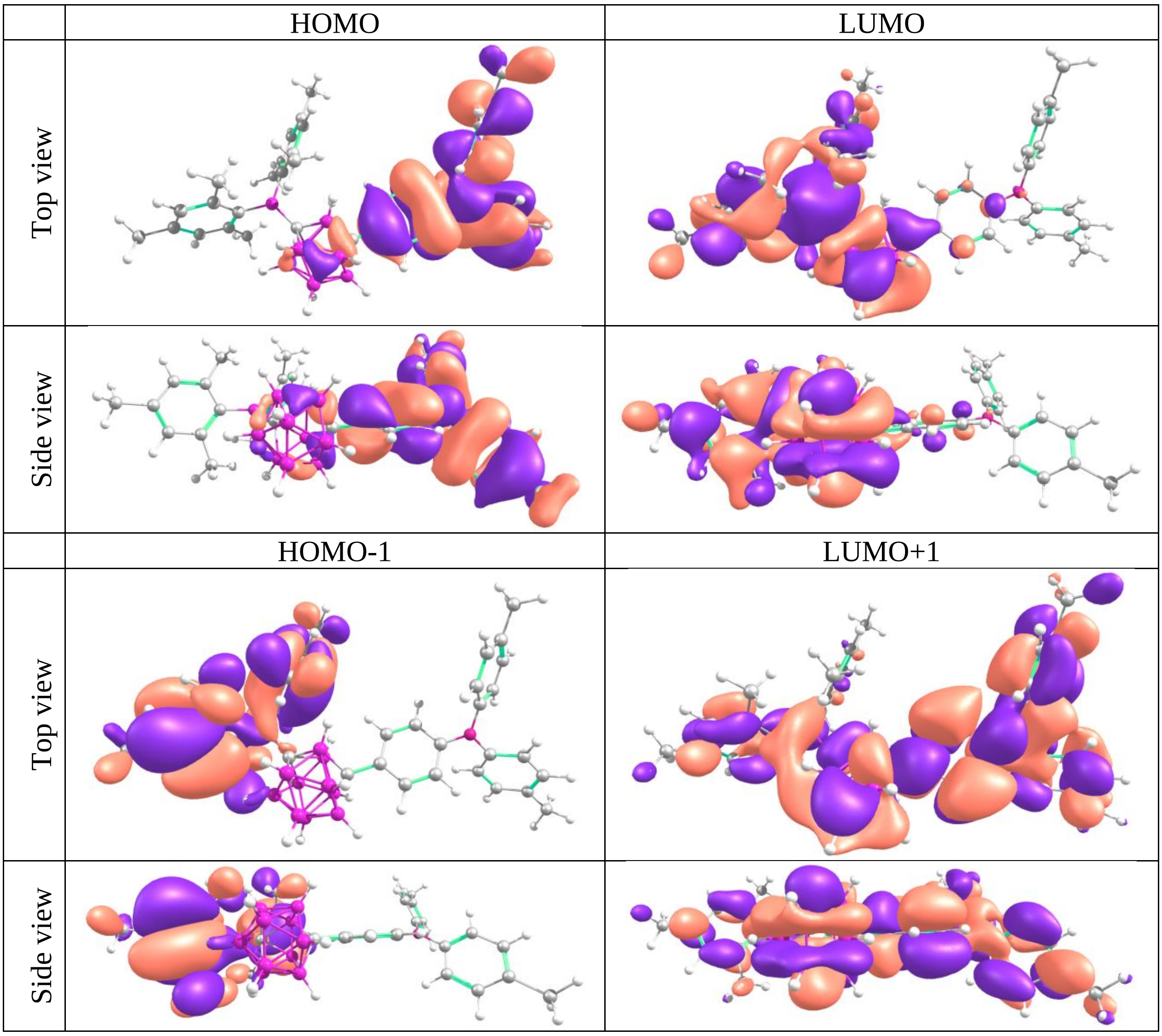

Supplementary Fig. 26. Frontier molecular orbitals of **DA-*m*Carb** in side and top view. Purple and orange indicate the different signs of the wave function.

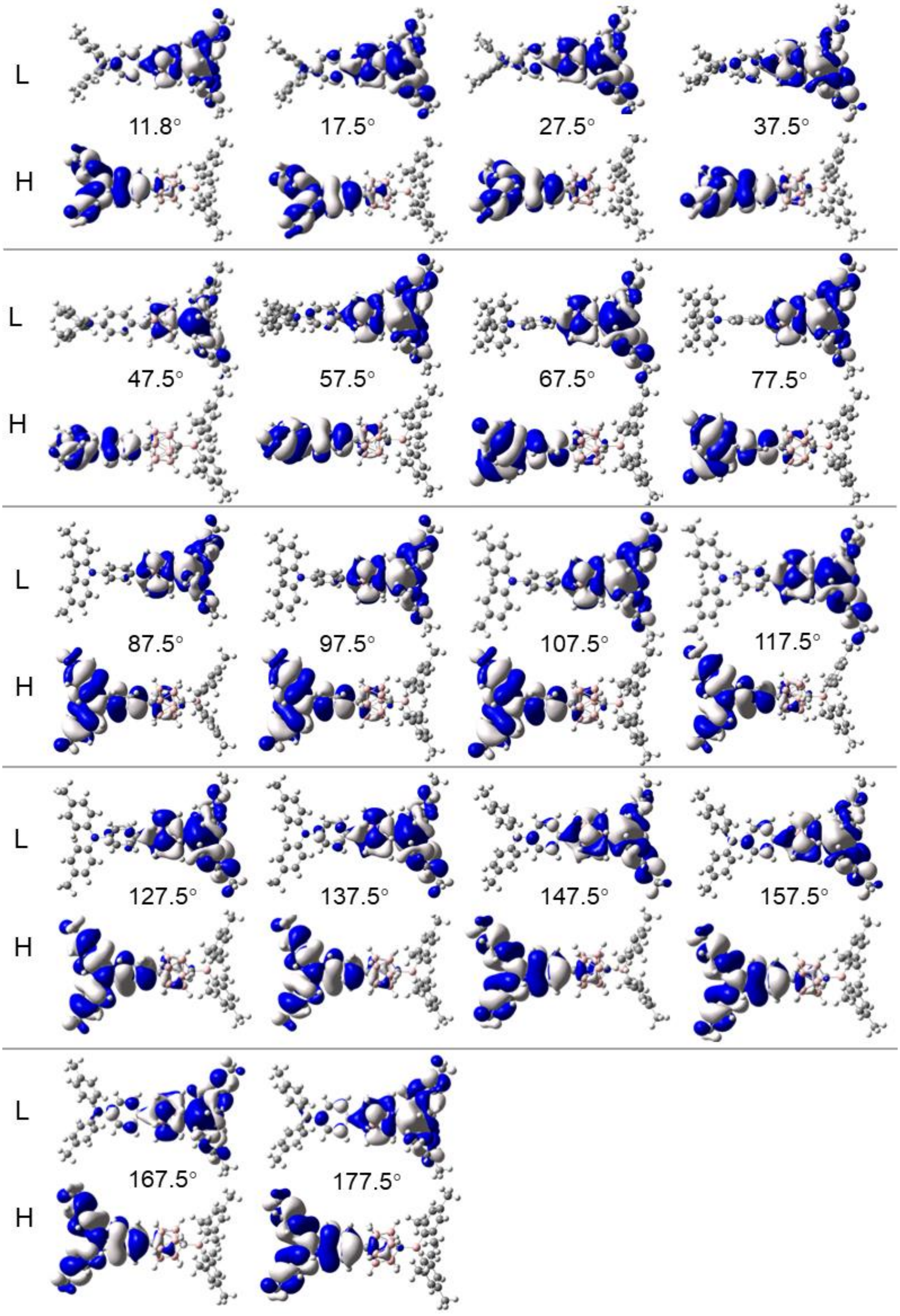

Supplementary Fig. 27. Molecular orbitals of **DA-*p*Carb** in different orientations. Blue and white indicate the different signs of the wave function.

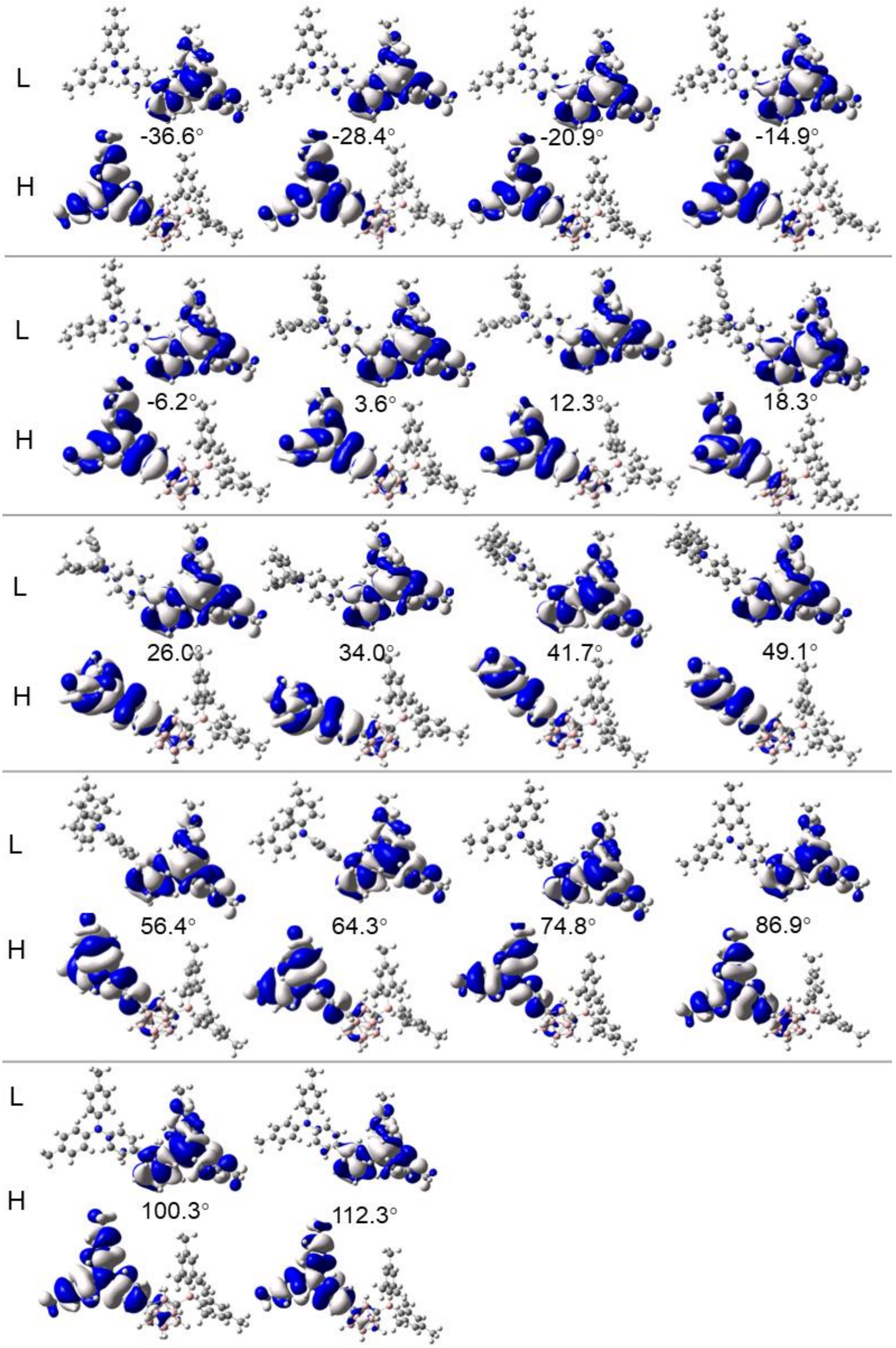

Supplementary Fig. 28. Molecular orbitals of **DA-*m*Carb** in different orientations. Blue and white indicate the different signs of the wave function.

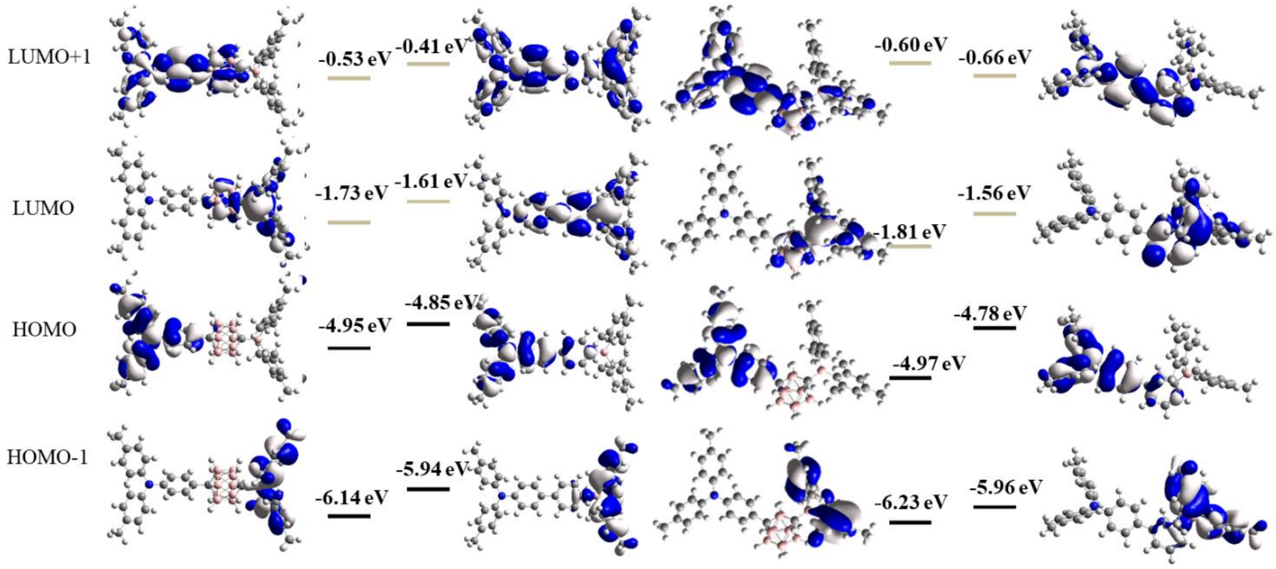

Supplementary Fig. 29. Comparison of **DA-*p*Carb**, **DA-*p*Benz**, **DA-*m*Carb**, and **DA-*m*Benz** frontier molecular orbitals (B3LYP/6-31G*). Blue and white indicate the different signs of the wave function.

2.3.2 NAO.

Supplementary Tab. 7. Orbital composition analysis of fragment contribution of **DA-*p*Carb** and **DA-*m*Carb** by natural atomic orbital (NAO) method at B3LYP/6-31G* level, the values are the fragments contributions to the molecular orbitals.

	**Orbitals**	**BMes2-**	**B atom of BMes2 group**	**carborane**	**triarylamine**
**DA-*p*Carb**	HOMO-1	93.246 %	1.189 %	1.617 %	0.094 %
	HOMO	0.120%	0.072 %	1.254 %	96.783 %
	LUMO	79.726 %	60.460 %	14.725 %	3.059 %
	LUMO+1	9.273 %	3.380 %	14.535 %	74.443 %
	**Orbitals**	**BMes2-**	**B atom**	**carborane**	**triarylamine**
**DA-*m*Carb**	HOMO-1	94.185 %	0.500 %	0.403 %	0.027 %
	HOMO	0.008 %	0.003 %	1.370 %	96.788 %
	LUMO	82.020 %	61.729 %	14.322 %	1.139 %
	LUMO+1	6.655 %	1.820 %	16.984 %	74.907 %

Supplementary Tab. 8. Orbital composition analysis of fragment contribution of **DA-*p*Benz** and **DA-*m*Benz** by natural atomic orbital (NAO) method at B3LYP/6-31G* level, the values are the fragments contributions to the molecular orbitals.

	**Orbitals**	**BMes2-**	**B atom**	**benzene**	**triarylamine**
**DA-mBenz**	HOMO-1	93.244 %	0.370 %	5.253 %	1.505 %
	HOMO	0.088 %	0.027 %	5.433 %	94.481 %
	LUMO	65.288 %	42.604 %	33.692 %	1.015%
	LUMO+1	4.603 %	1.273 %	37.883 %	57.509 %
	**Orbitals**	**BMes2-**	**B atom**	**benzene**	**triarylamine**
**DA-*p*Benz**	HOMO-1	95.821 %	0.102 %	4.069 %	0.116 %
	HOMO	0.019 %	0.001 %	0.227 %	99.762 %
	LUMO	96.034 %	83.067 %	3.656 %	0.312 %
	LUMO+1	0.271 %	0.044 %	10.964 %	88.757 %

2.3.3 TD-DFT.

Both absorption spectra calculated of **DA-*p*Carb** and **DA-*m*Carb** at the gas-phase optimized structure at B3LYP/6-31G* display a charge transfer (CT) transition at 426 and 438 nm. However, in agreement with chemical intuition, it is a HOMO-LUMO transition. Take **DA-*p*Carb** as an example, the emission maximum is at 505.7 nm, which supports the CT character. At CAM-B3LYP/6-31G* the lowest energy transition is at 300.7 nm (in better agreement with experiment) but the character is totally mixed with no HOMO-LUMO contribution and no CT character. Even in THF, this situation does not change.

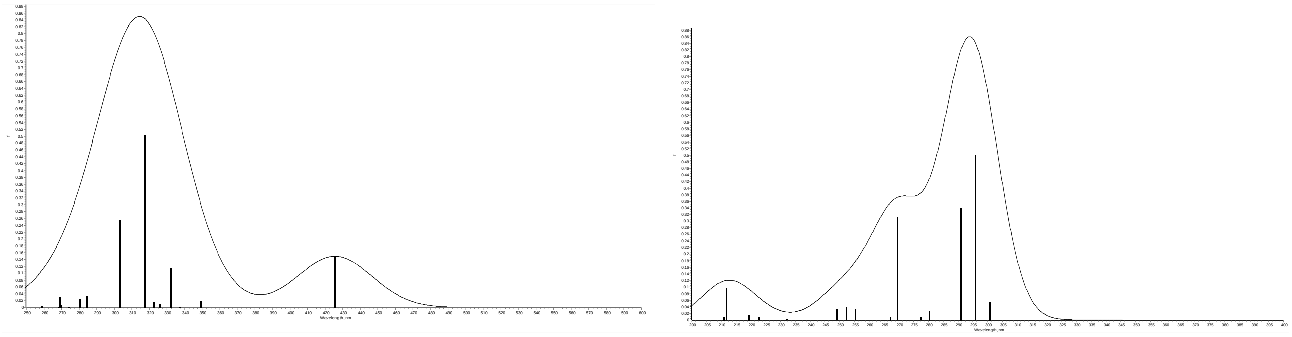

Supplementary Fig. 30. Calculated absorption spectrum of **DA-*p*Carb** in the gas phase at S_0 geometry C39-N38-B23-C4 = 39.8°. (Left): calculated at B3LYP/6-31G* level; (right): calculated at CAM-B3LYP/6-31G* level.

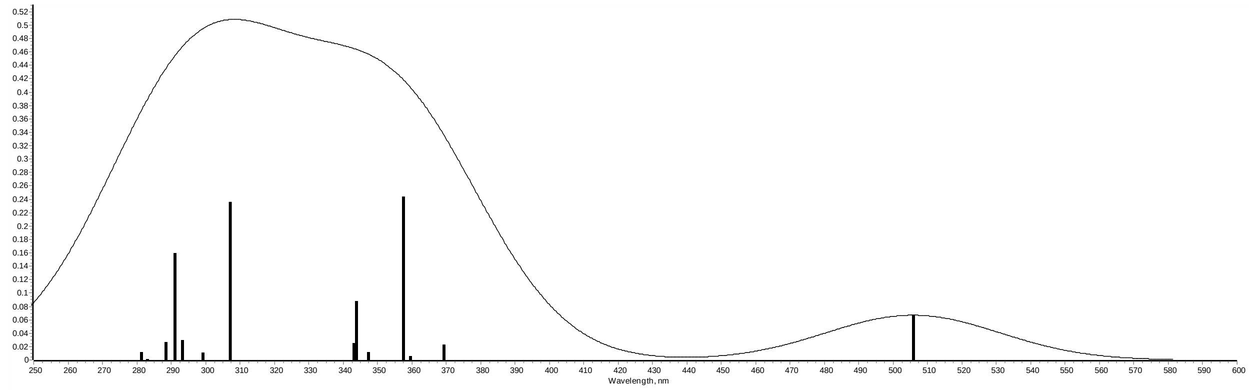

Supplementary Fig. 31. Calculated absorption spectrum of **DA-*p*Carb** in the gas phase at S_1 geometry; C39-N38-B23-C4 = 55.5°. The lowest excitation here refers to the red shifted emission spectrum. The higher energy transition should be disregarded.

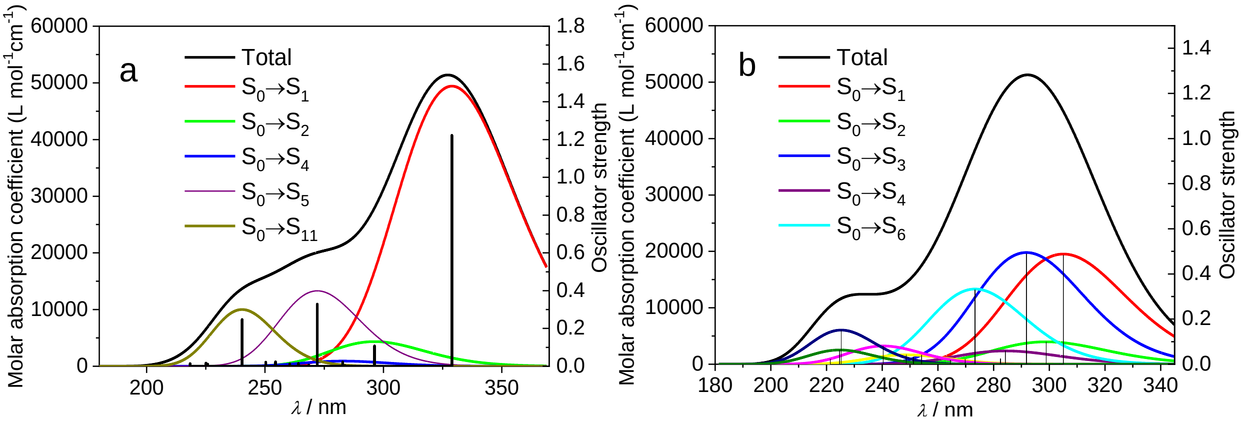

Supplementary Fig. 32. TD-DFT calculated UV-vis absorption spectra of **DA-*p*Benz** (a), and **DA-*m*Benz** (b) in the gas phase at the B3LYP/6-31G* level. Lines in different colors here represent different transition.

Supplementary Tab. 9. Lowest energy singlet electronic transitions of **DA-*p*Carb** (TD-DFT at B3LYP/6-31G*, gas phase).

State	E / eV	λ / nm	Oscillator strength	Transitions	Contribution
1	2.9138	425.51	0.1504	HOMO→LUMO	0.9941
2	3.5497	349.28	0.0201	HOMO-3→LUMO<br>HOMO-2→LUMO<br>HOMO-1→LUMO	0.0971<br>0.0804<br>0.7869
3	3.6789	337.01	0.0023	HOMO-2→LUMO<br>HOMO-1→LUMO	0.8503<br>0.1199
4	3.7330	332.13	0.1140	HOMO-4→LUMO<br>HOMO-3→LUMO<br>HOMO-2→LUMO<br>HOMO-1→LUMO	0.0814<br>0.7563<br>0.0553<br>0.0719
5	3.8082	325.57	0.0088	HOMO-3→LUMO<br>HOMO-4→LUMO	0.0990<br>0.8846

Supplementary Tab. 10. Lowest energy singlet electronic transitions of **DA-*p*Benz** (TD-DFT at B3LYP/6-31G*, gas phase).

State	E / eV	λ / nm	Oscillator Strength	Transitions	Contribution
1	2.8938	428.44	0.5829	HOMO→LUMO	0.9852
2	3.5972	344.67	0.0726	HOMO-1→LUMO	0.9759
3	3.6975	335.32	0.1341	HOMO-2→LUMO	0.9476
4	3.7691	328.95	0.0379	HOMO-3→LUMO	0.9686
5	3.8074	325.64	0.0130	HOMO→LUMO+2	0.9614

Supplementary Tab. 11. Lowest energy singlet electronic transitions of **DA-*m*Carb** (TD-DFT B3LYP/6-31G*, gas phase).

State	E / eV	λ / nm	Oscillator strength	Transitions	Contribution
1	2.8286	438.32	0.0280	HOMO→LUMO	0.9965
2	3.5933	345.04	0.0122	HOMO-3→LUMO<br>HOMO-2→LUMO<br>HOMO-1→LUMO	0.3623<br>0.2522<br>0.3634
3	3.6848	336.47	0.0097	HOMO-3→LUMO<br>HOMO-2→LUMO<br>HOMO-1→LUMO	-0.1628<br>-0.2085<br>0.6163
4	3.7073	334.43	0.1637	HOMO-3→LUMO<br>HOMO-2→LUMO	-0.4436<br>0.5074
5	3.8185	324.69	0.0066	HOMO-4→LUMO	0.9799

Supplementary Tab. 12. Lowest energy singlet electronic transitions of **DA-*m*Benz** (TD-DFT at B3LYP/6-31G*, gas phase).

State	*E* / eV	λ / nm	Oscillator strength	Transitions	Contribution
1	2.8538	434.45	0.0220	HOMO→LUMO	0.9923
2	3.6037	344.05	0.4810	HOMO-2→LUMO<br>HOMO-1→LUMO<br>HOMO→LUMO+1	0.0467<br>0.1743<br>0.7517
3	3.6192	342.58	0.3133	HOMO-2→LUMO<br>HOMO-1→LUMO<br>HOMO→LUMO+1	0.0267<br>0.7895<br>0.1412
4	3.7616	329.61	0.0183	HOMO-3→LUMO<br>HOMO-2→LUMO<br>HOMO→LUMO+1	0.0270<br>0.8907<br>0.0572
5	3.8029	326.03	0.0133	HOMO→LUMO+2	0.9649

2.3.4 Summary.

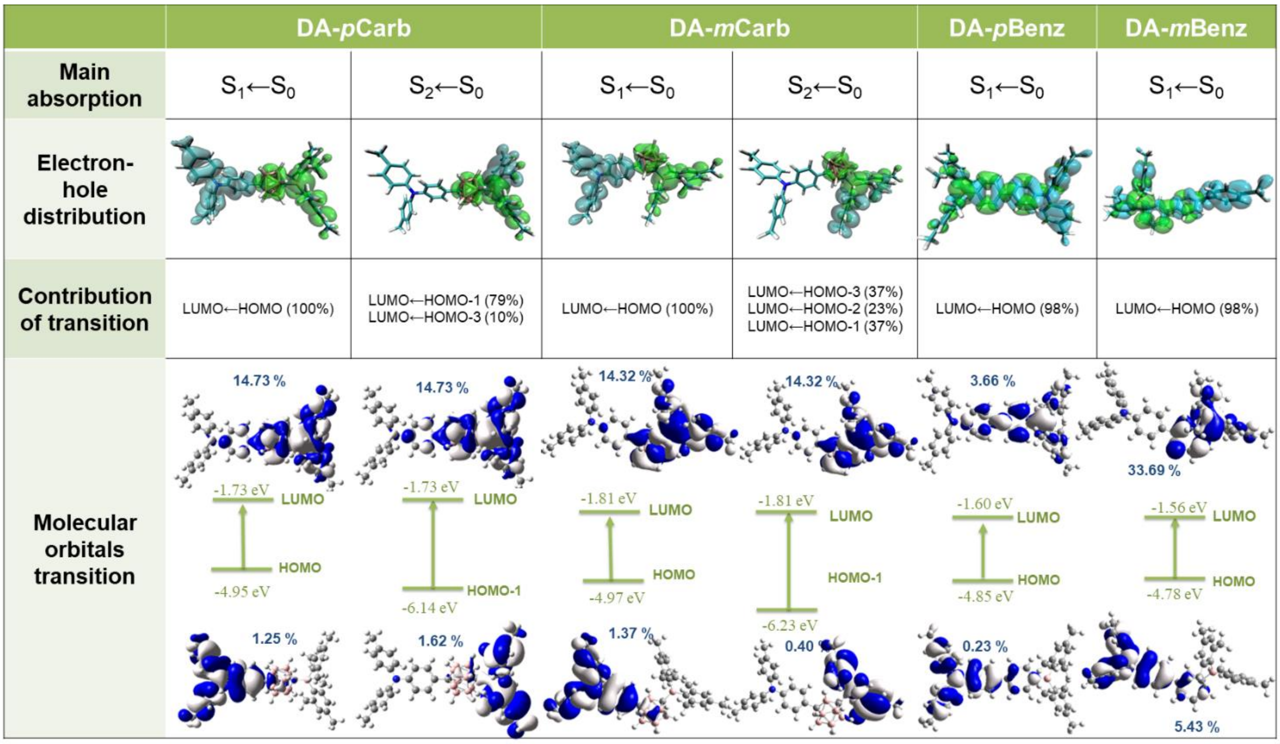

Supplementary Fig. 33. Summary of theoretical results. Main excitation, the distribution of hole (blue) and electron (green), the contribution of associated transitions and the distribution of HOMOs and LUMOs of **DA-*p*Carb**, **DA-*m*Carb**, the values are the "bridge" contributions to the corresponding molecular orbitals. Blue and white indicate the different signs of the wave function.

3. NMR Spectra.

^{11}B (proton-coupled) NMR spectrum of D-*p*Carb

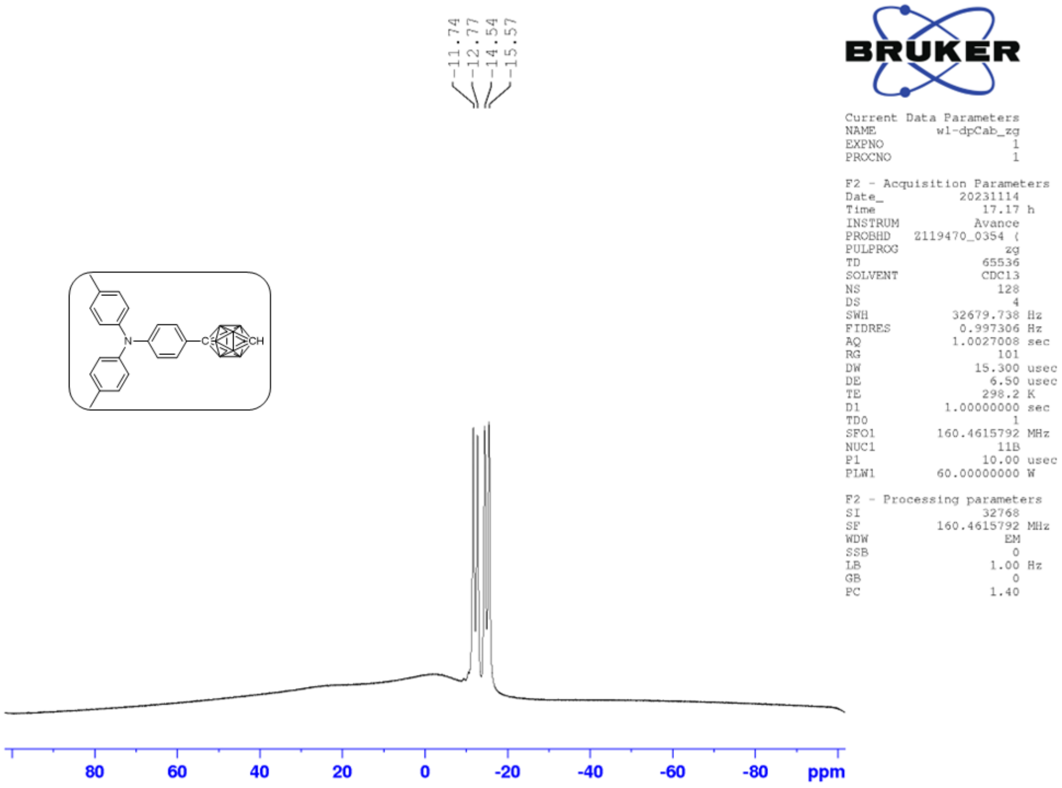

^{11}B{^{1}H} NMR spectrum of D-*p*Carb

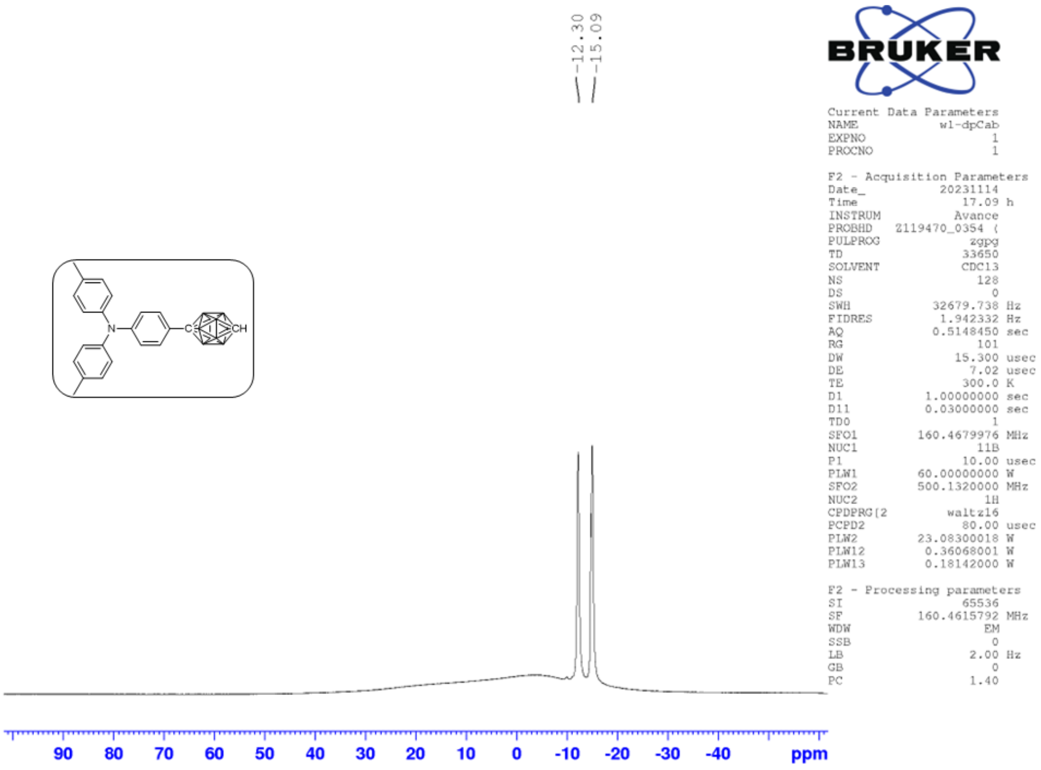

^{1}H NMR spectrum of D-*p*Carb

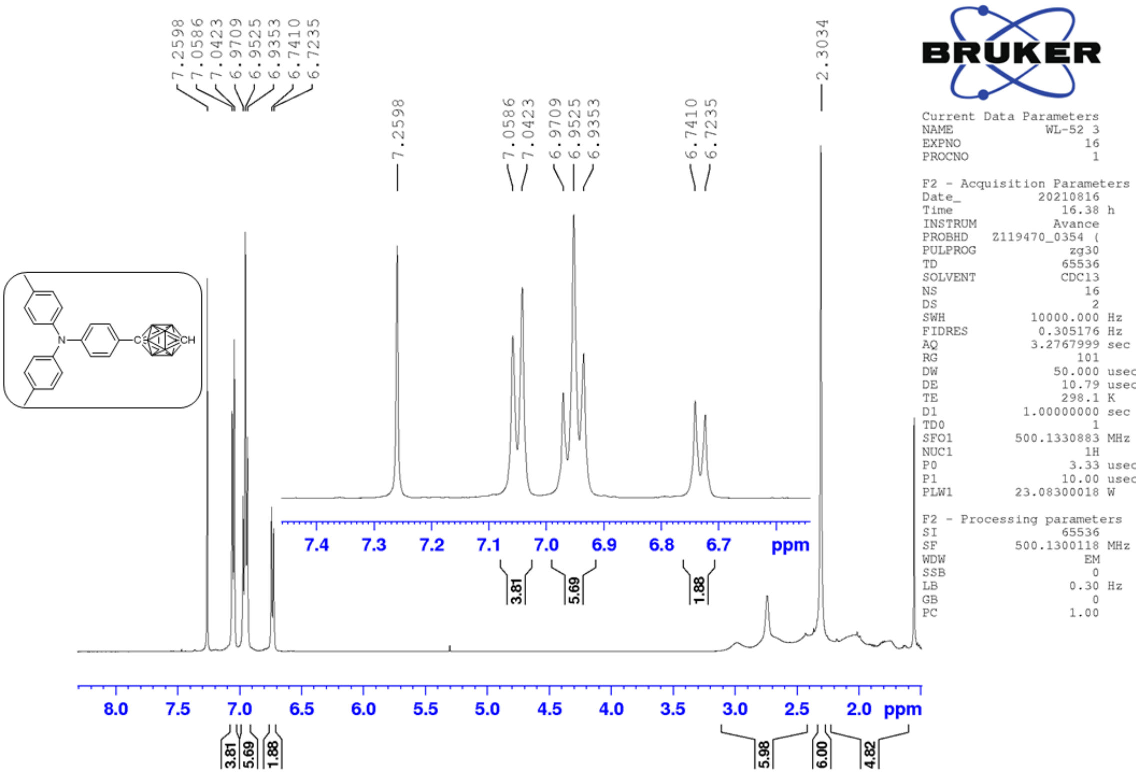

^{1}H{^{11}B} NMR spectrum of D-*p*Carb

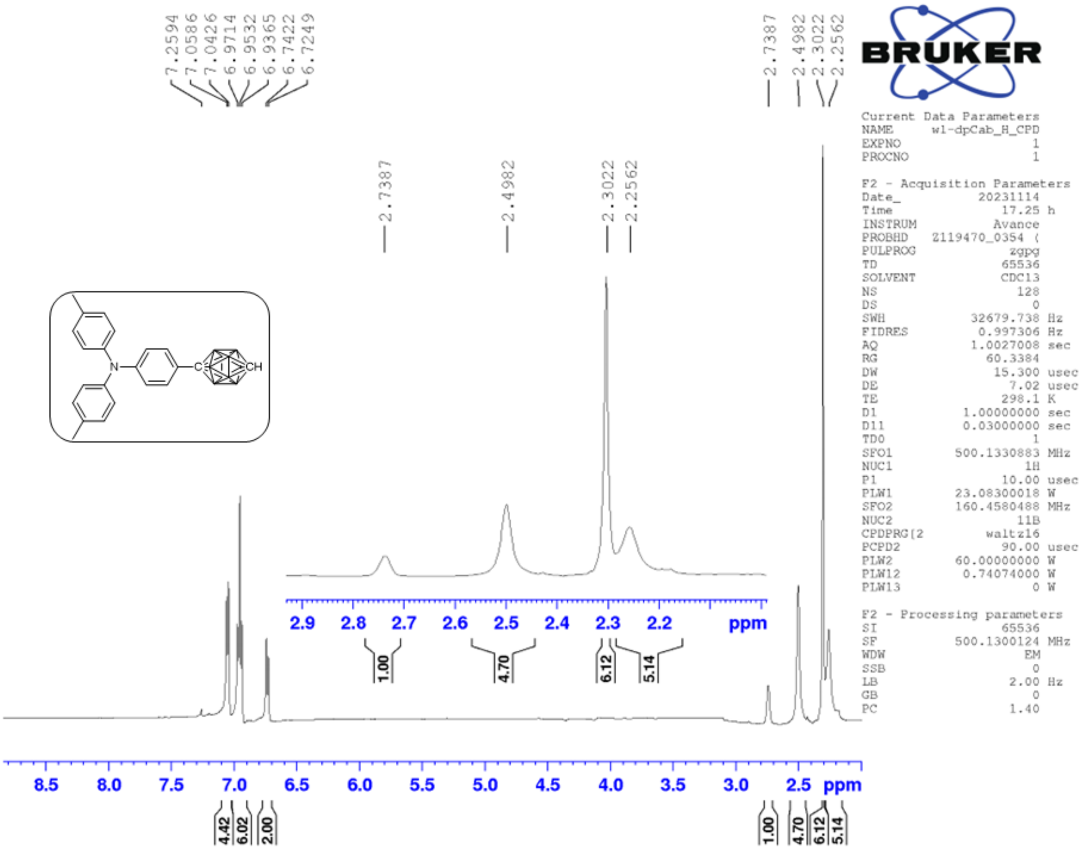

^{11}B (proton-coupled) NMR spectrum of DA-*p*Carb

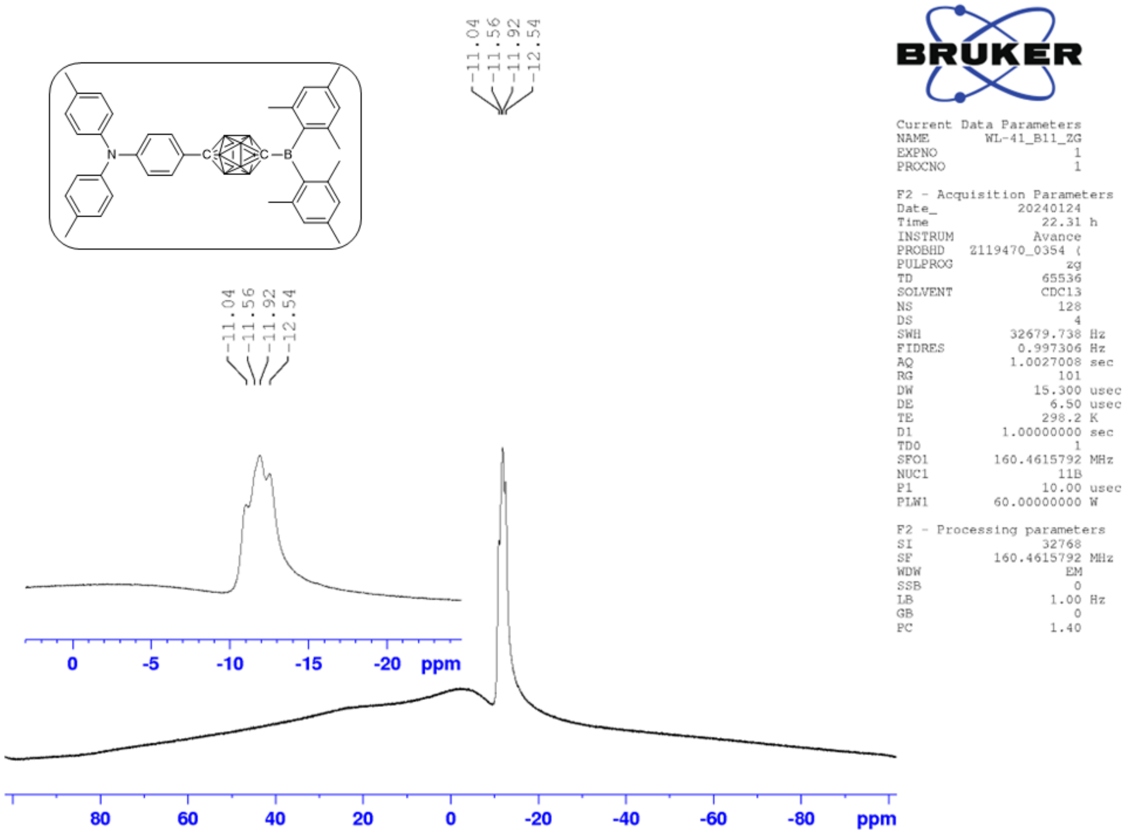

^{11}B{^{1}H} NMR spectrum of DA-*p*Carb

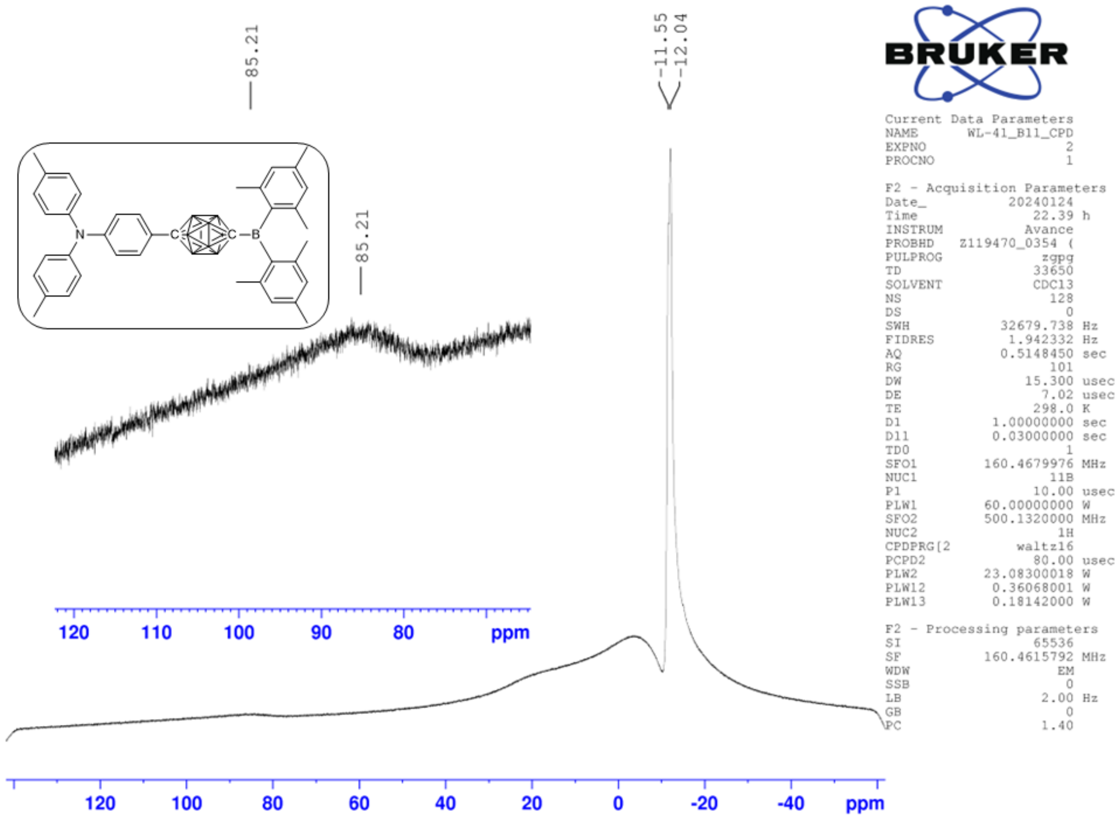

^{1}H NMR spectrum of DA-*p*Carb

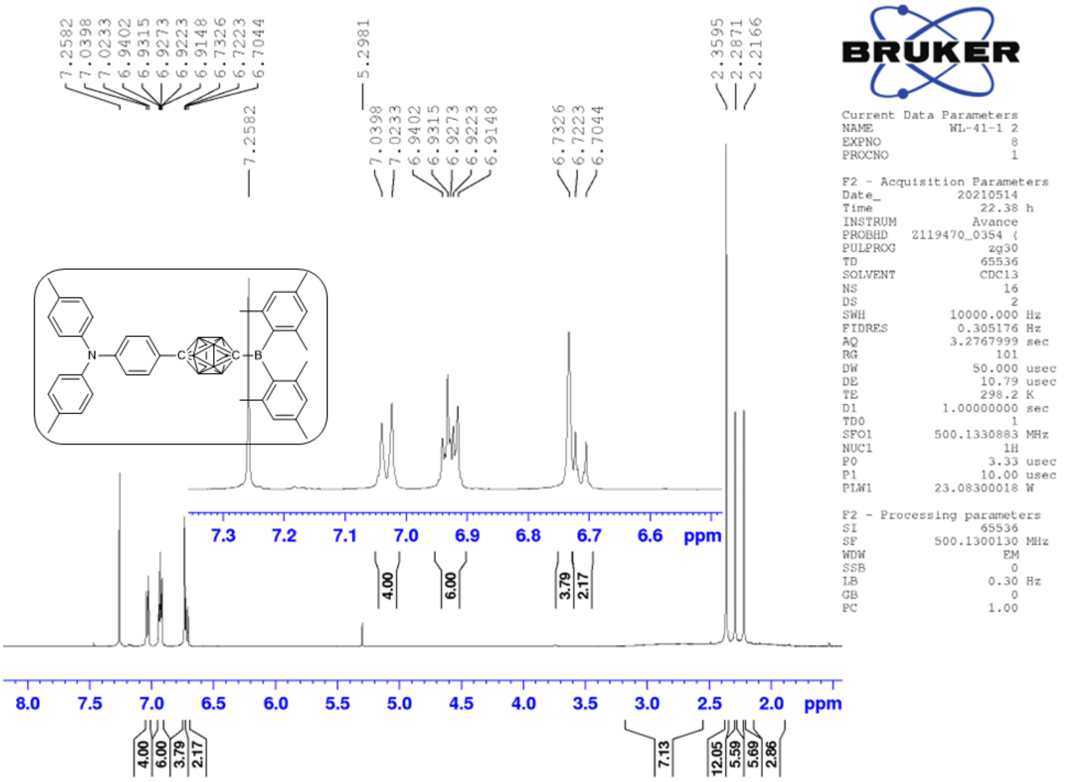

^{1}H{^{11}B} NMR spectrum of DA-*p*Carb

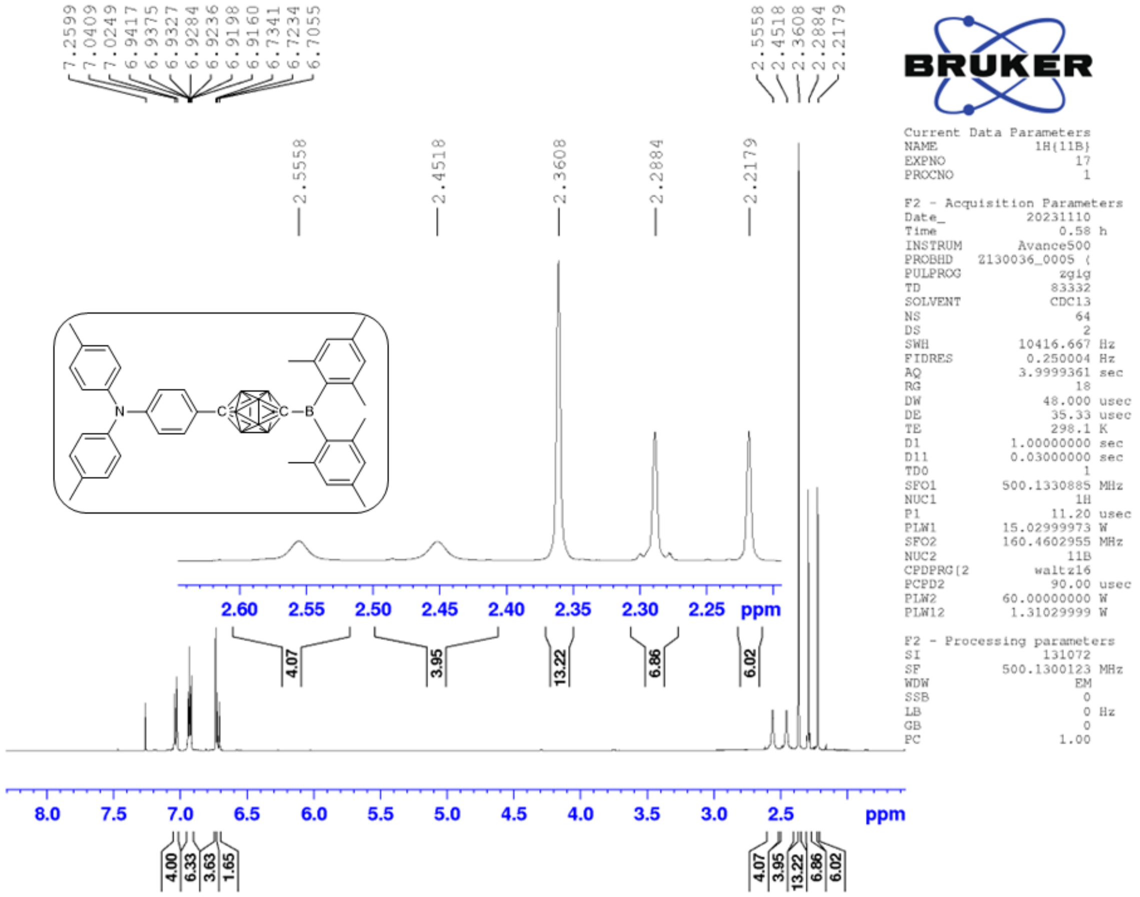

$^{13}C\{^{1}H\}$ NMR spectrum of DA-*p*Carb

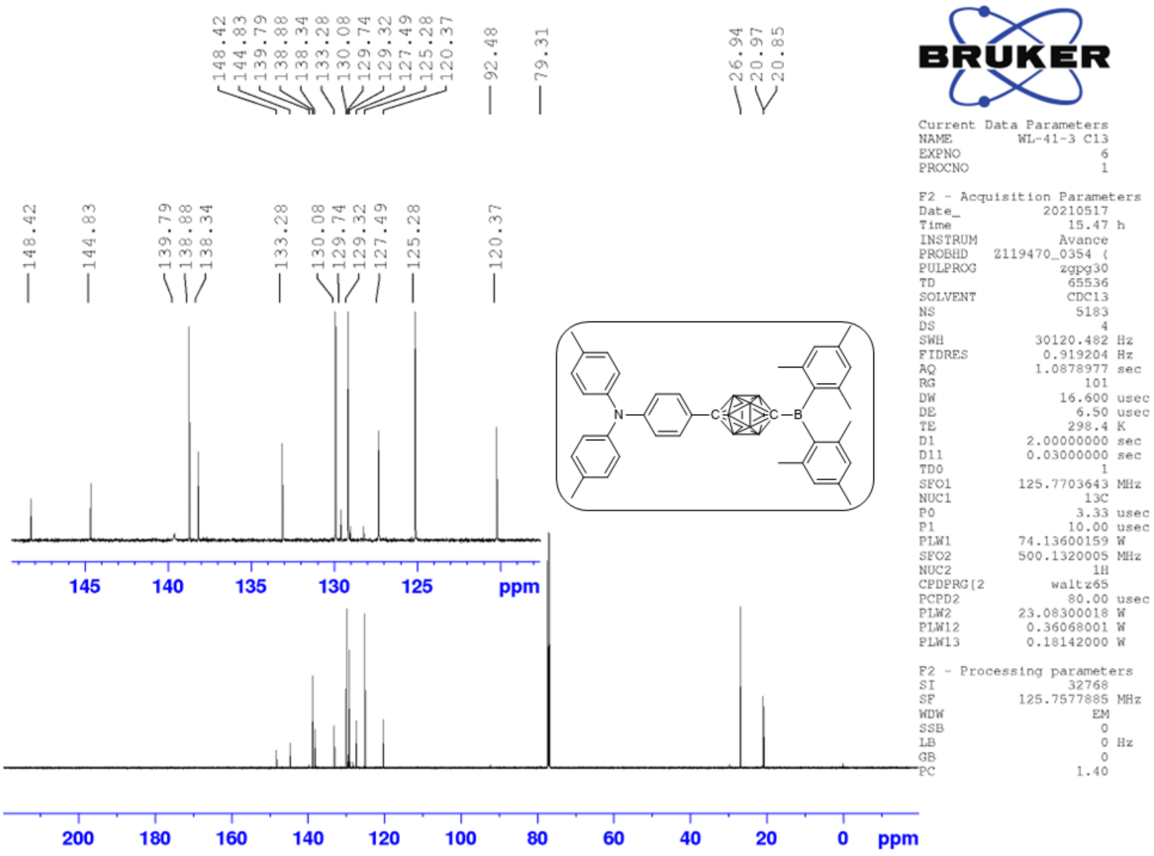

HRMS of DA-*p*Carb

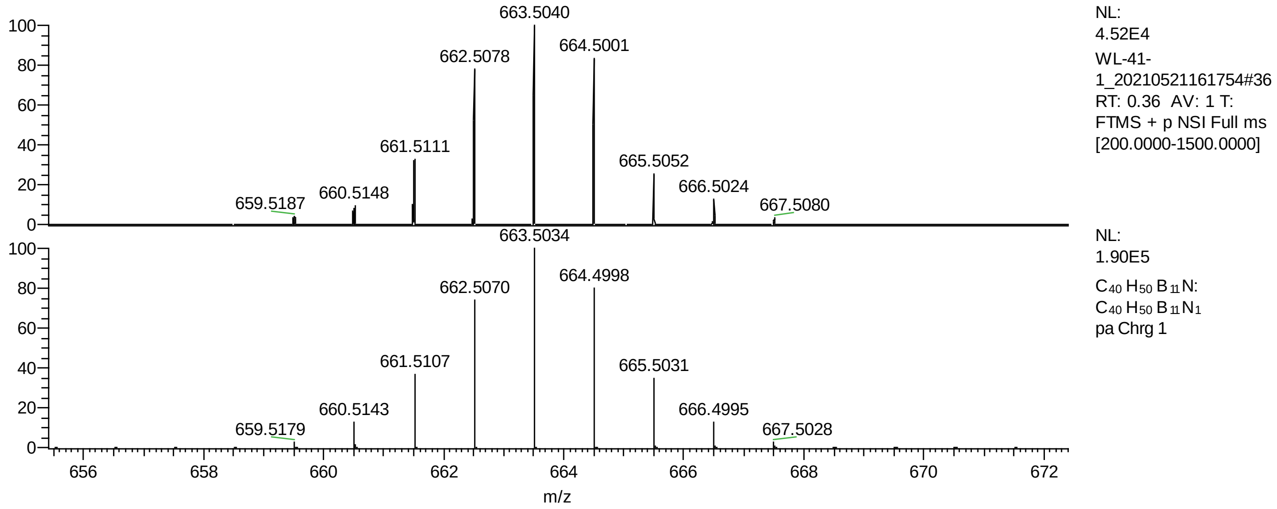

^{1}H NMR spectrum of DA-*p*Benz

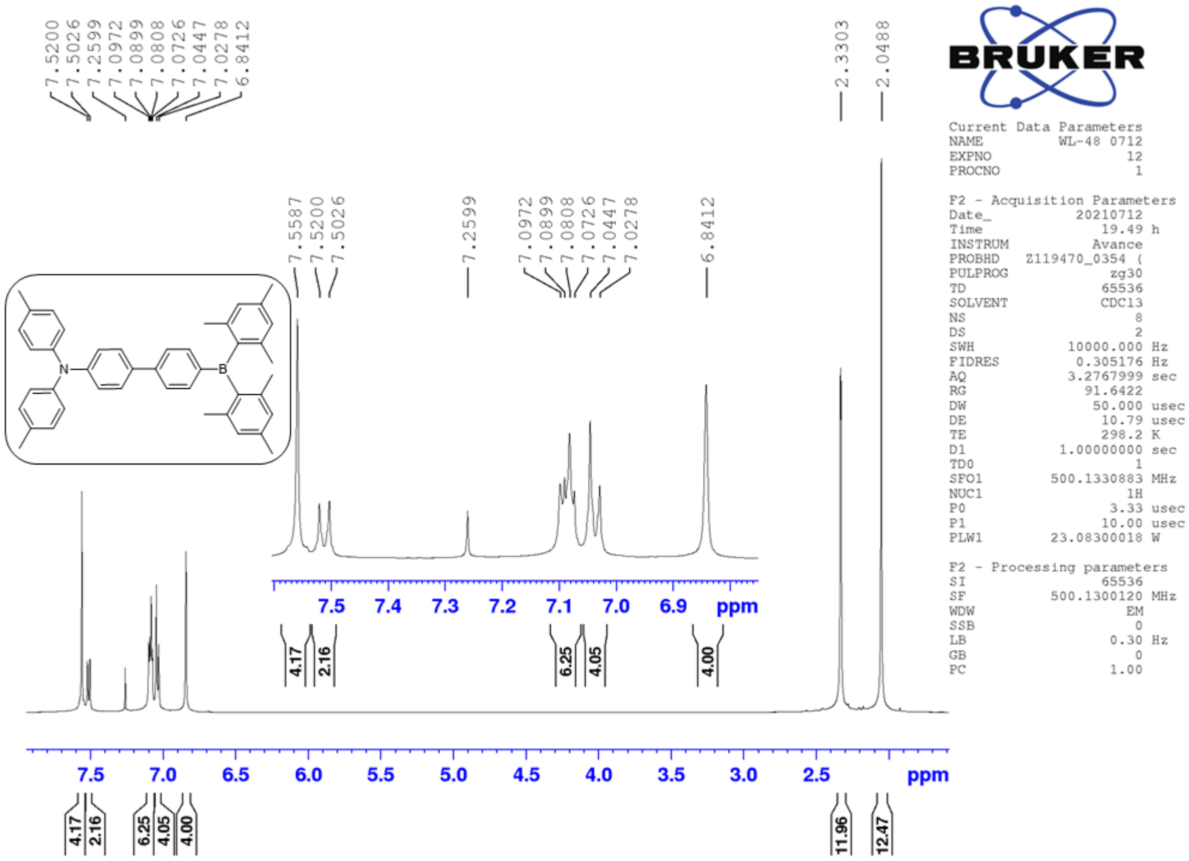

$^{13}C\{^1H\}$ NMR spectrum of DA-*p*Benz

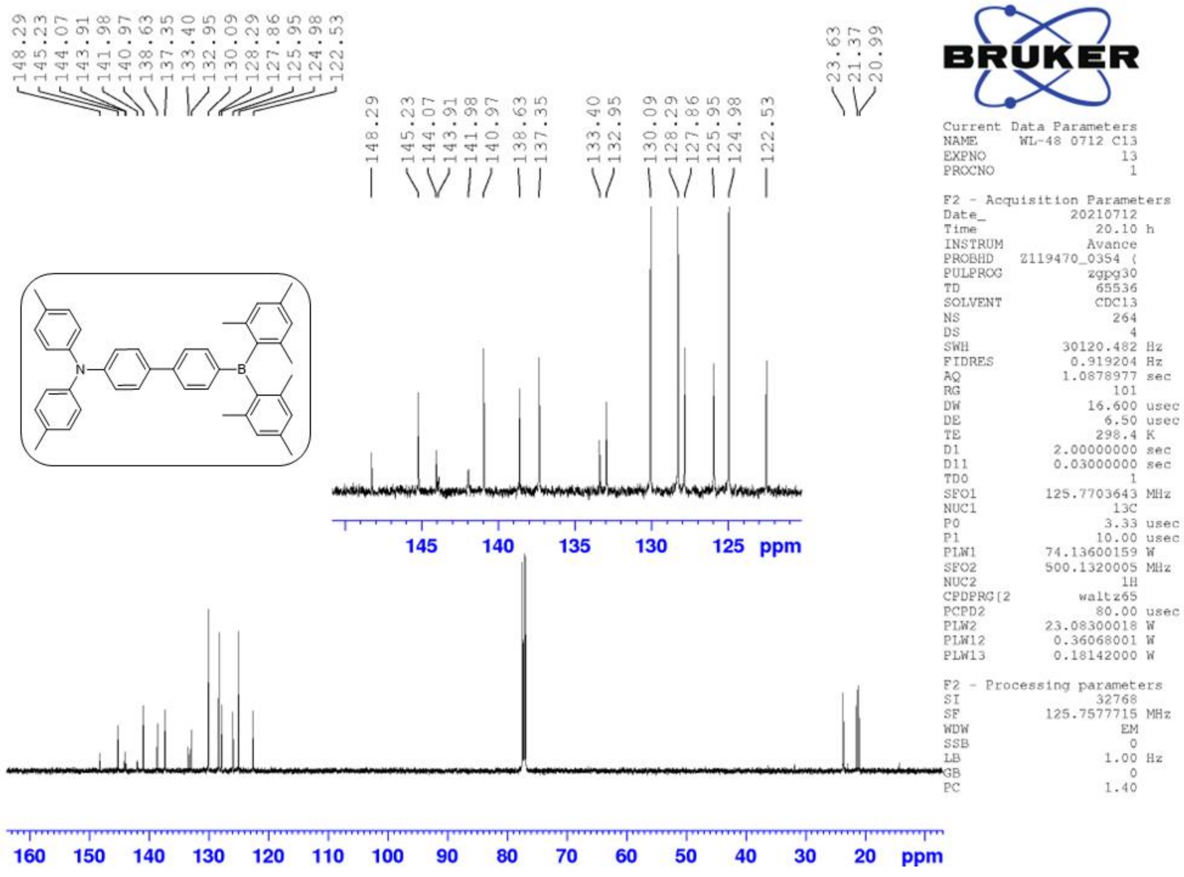

HRMS of DA-*p*Benz

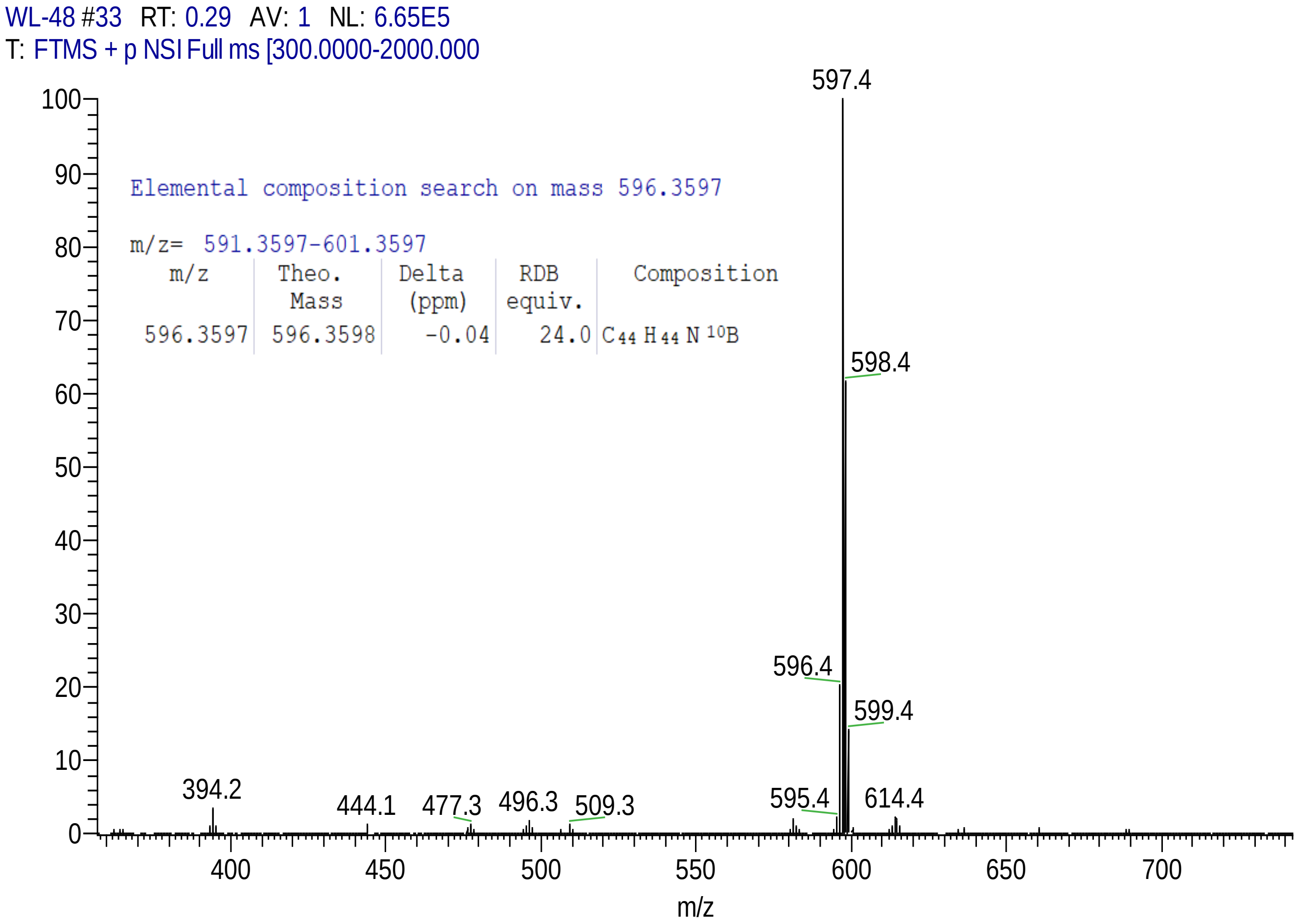

^{11}B (proton-coupled) NMR spectrum of D-*m*Carb

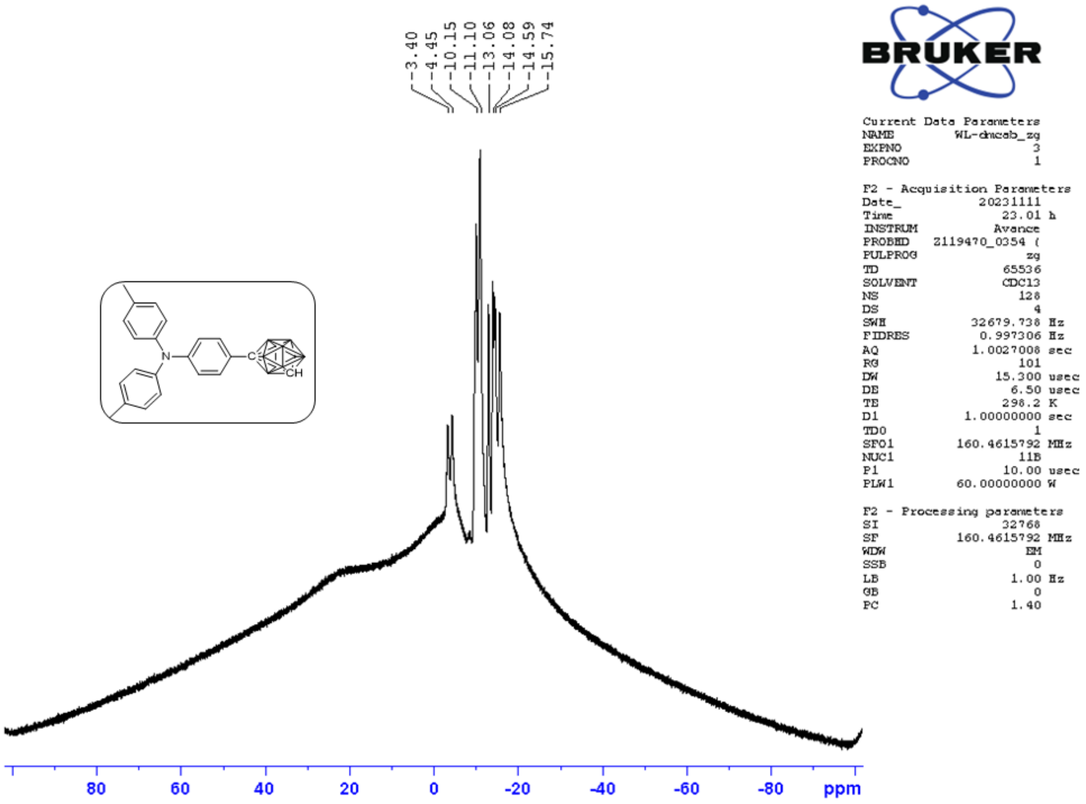

^{11}B{^{1}H} NMR spectrum of D-*m*Carb

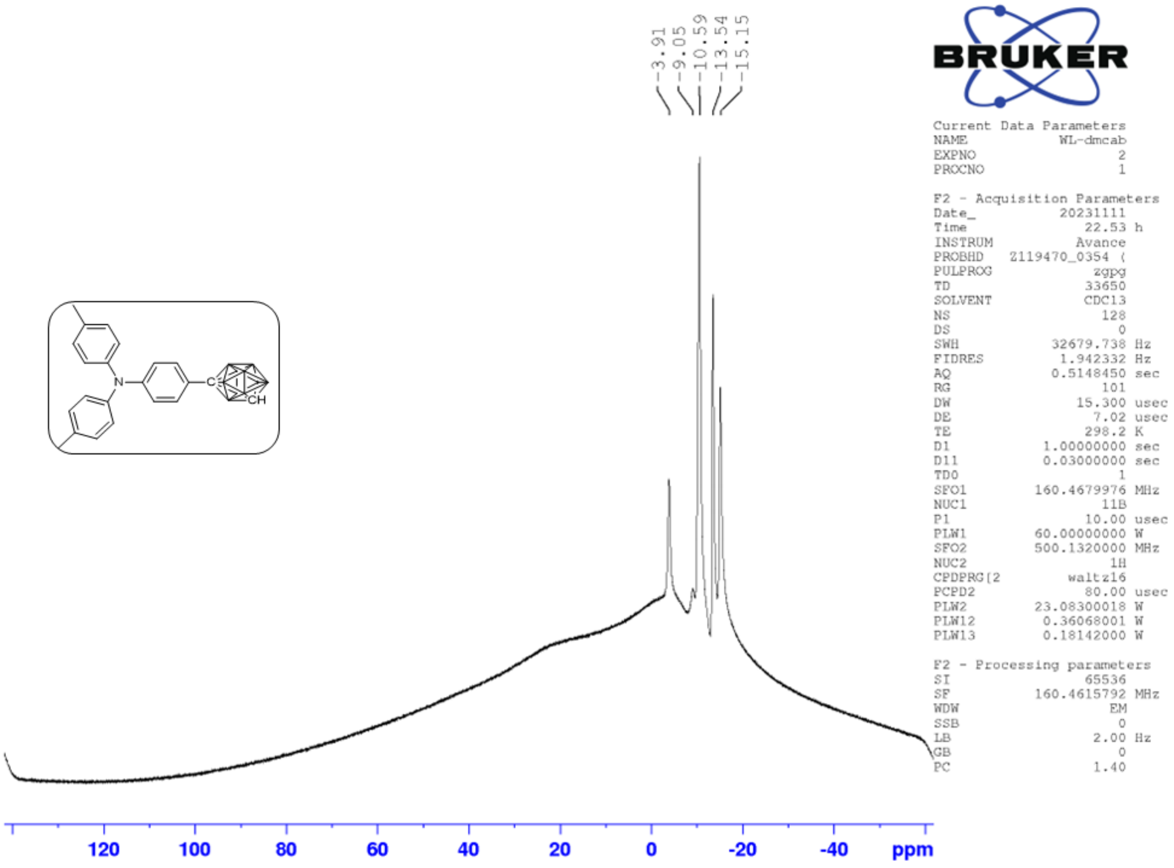

^{1}H NMR spectrum of D-*m*Carb

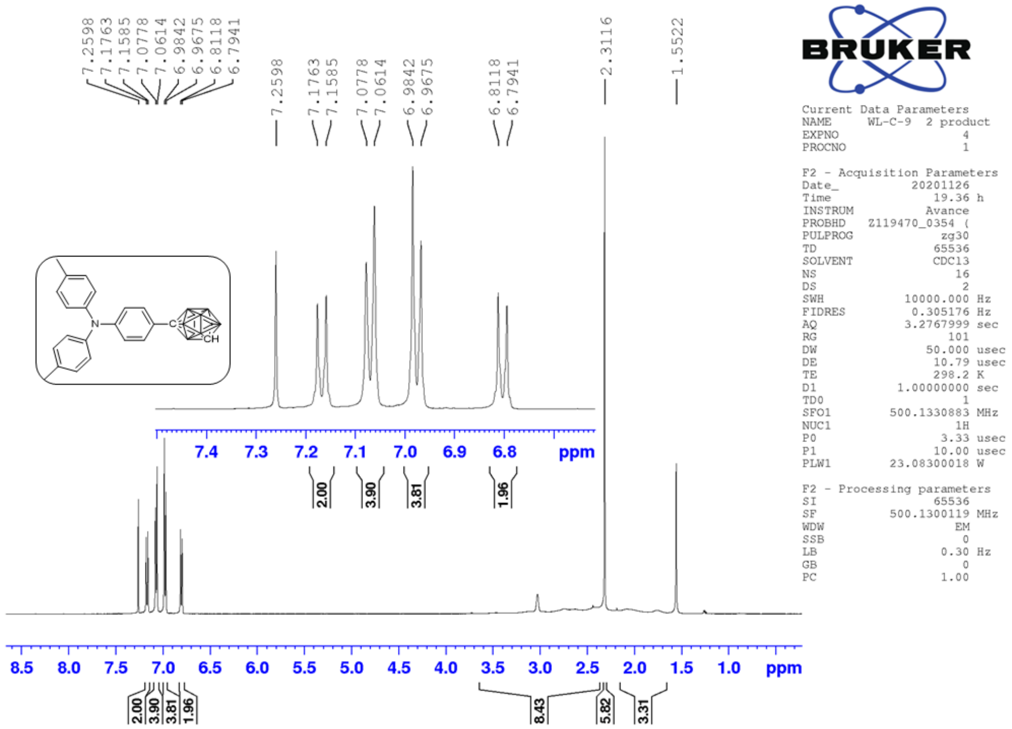

^{1}H {^{11}B} NMR spectrum of D-*m*Carb

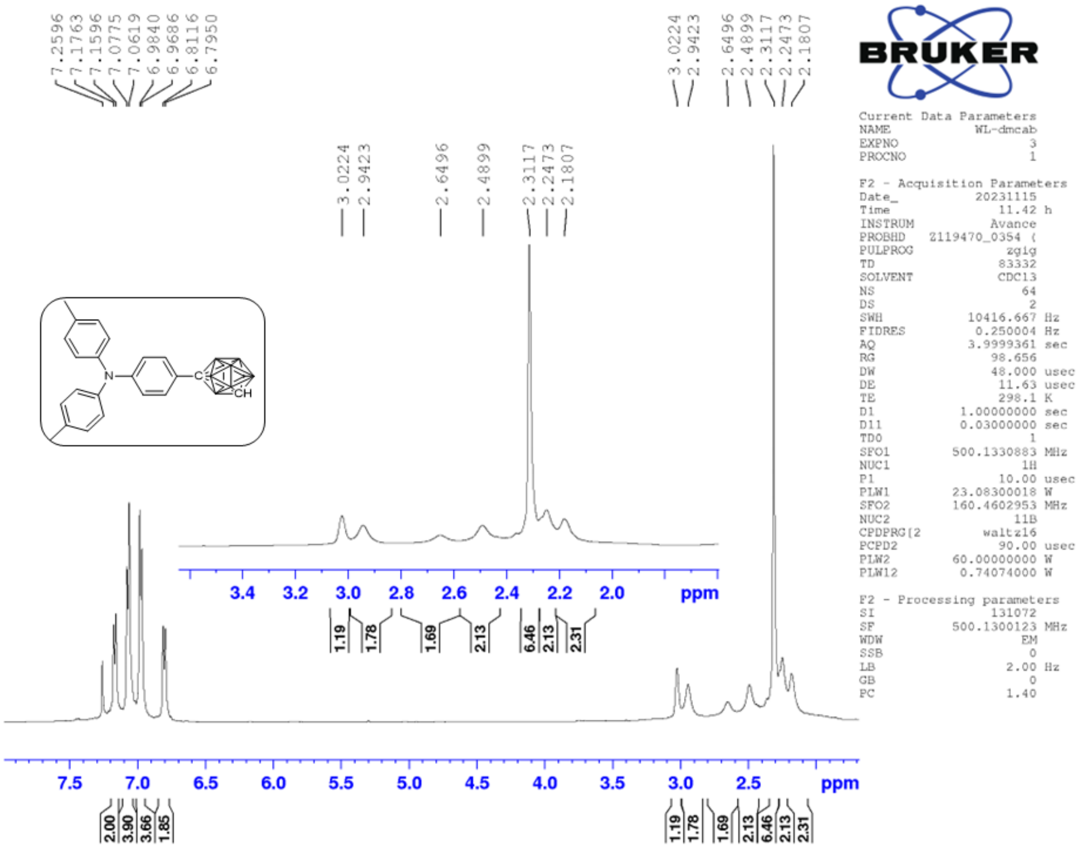

^{11}B (proton-coupled) NMR spectrum of DA-*m*Carb

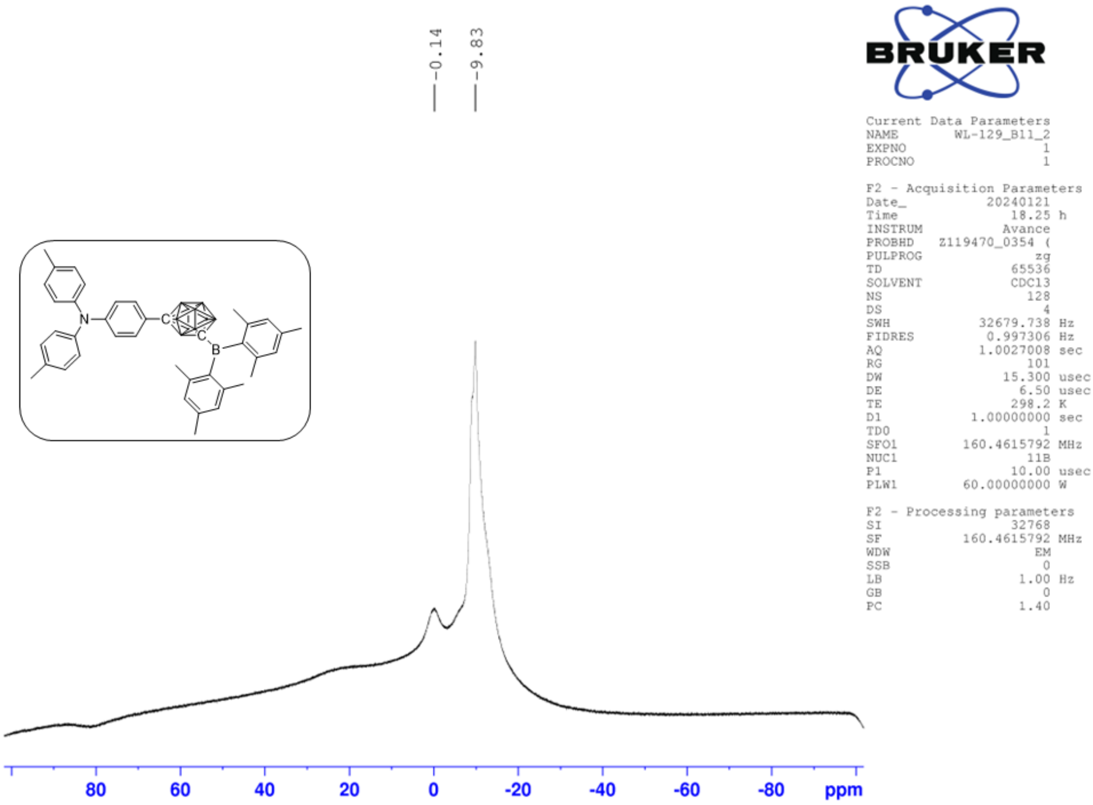

^{11}B{^{1}H} NMR spectrum of DA-*m*Carb

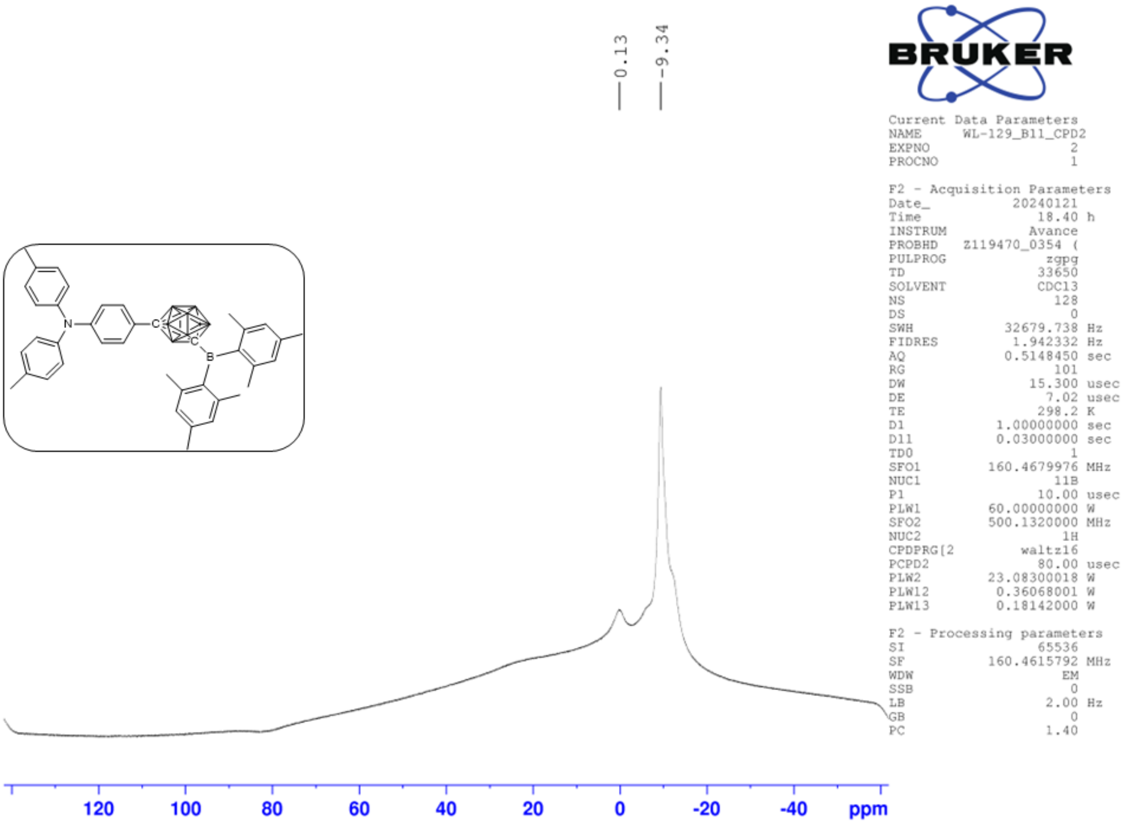

^{1}H NMR spectrum of DA-*m*Carb

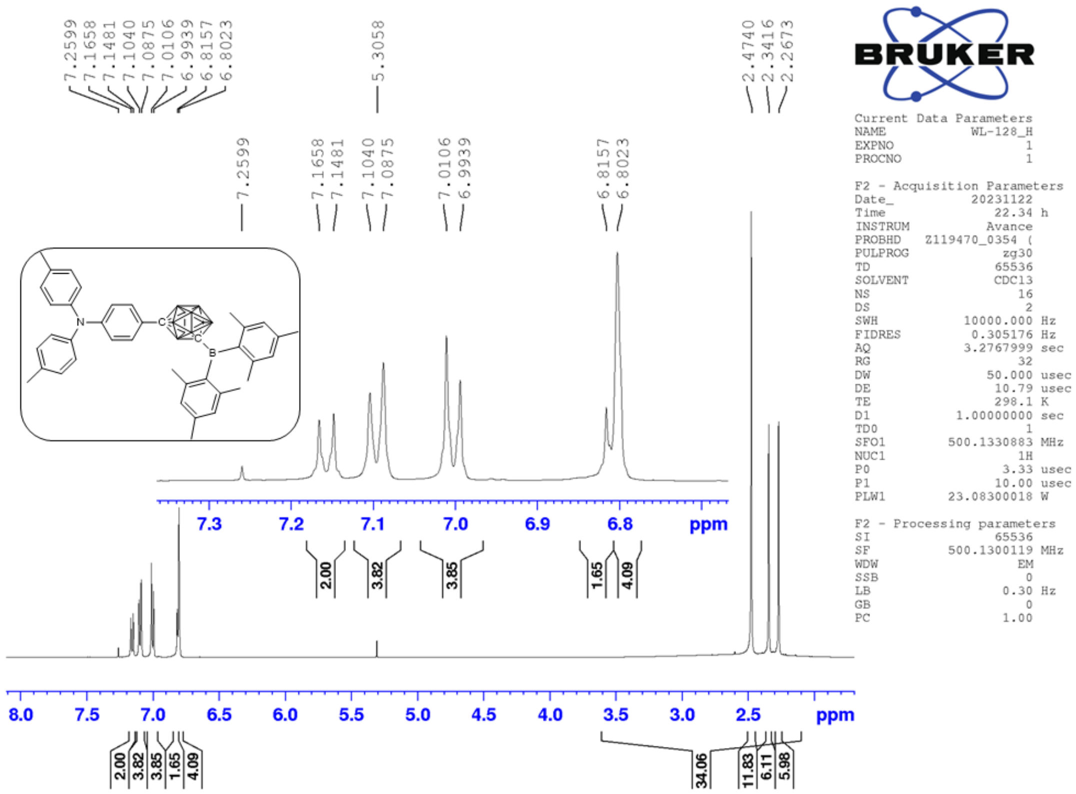

$^{1}H\{^{11}B\}$ NMR spectrum of DA-*m*Carb

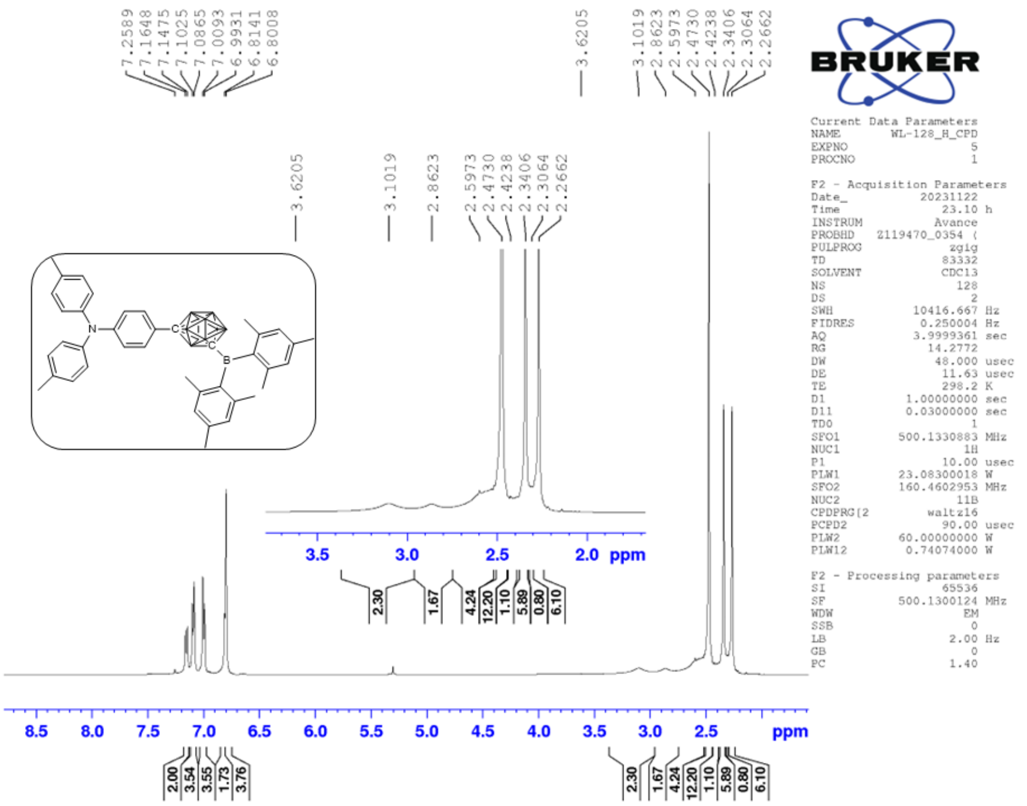

$^{13}C\{^{1}H\}$ NMR spectrum of DA-*m*Carb

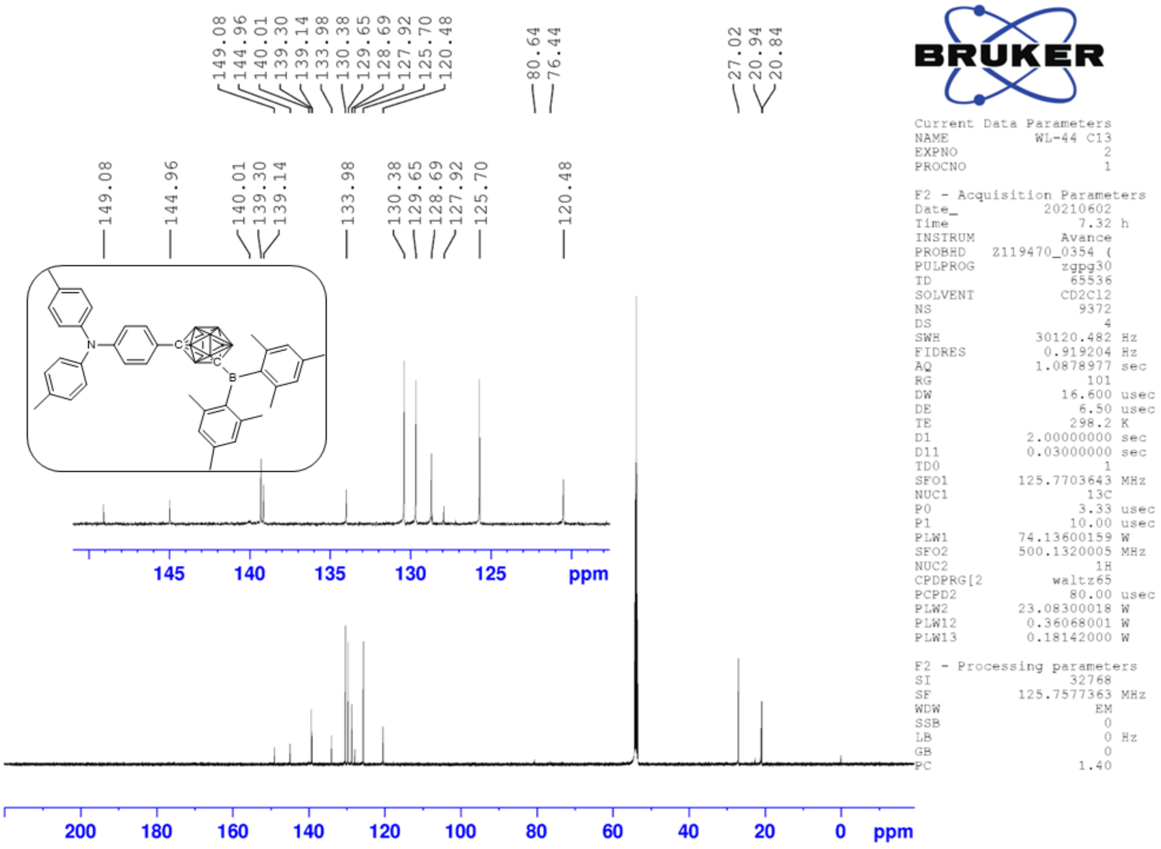

HRMS of DA-*m*Carb

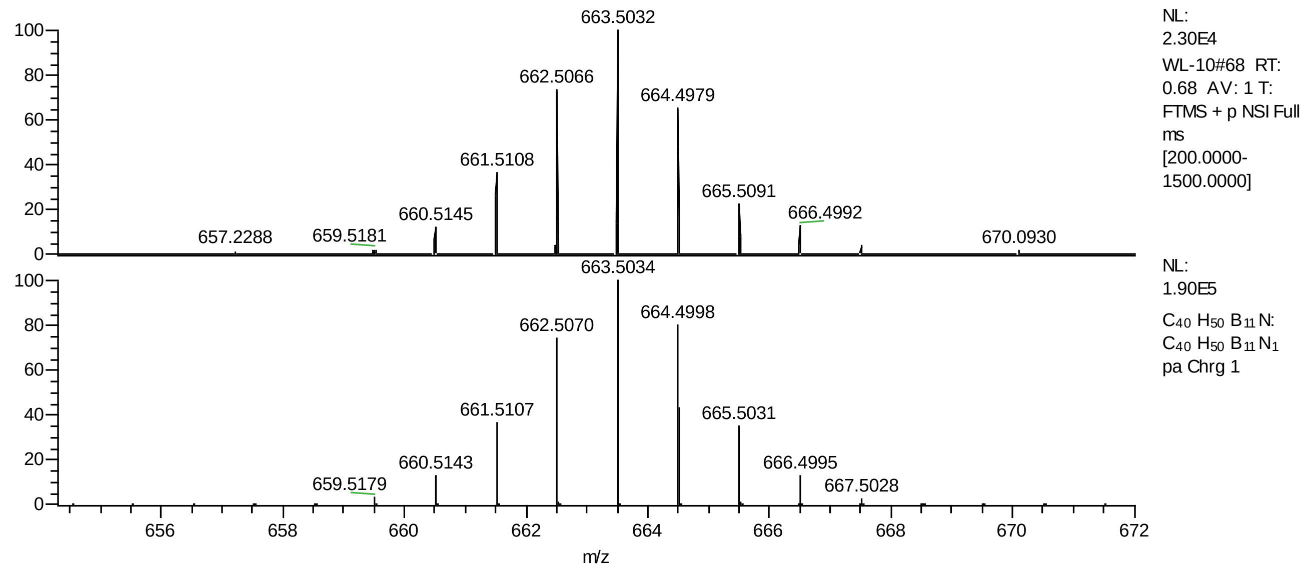

^{1}H NMR spectrum of 4

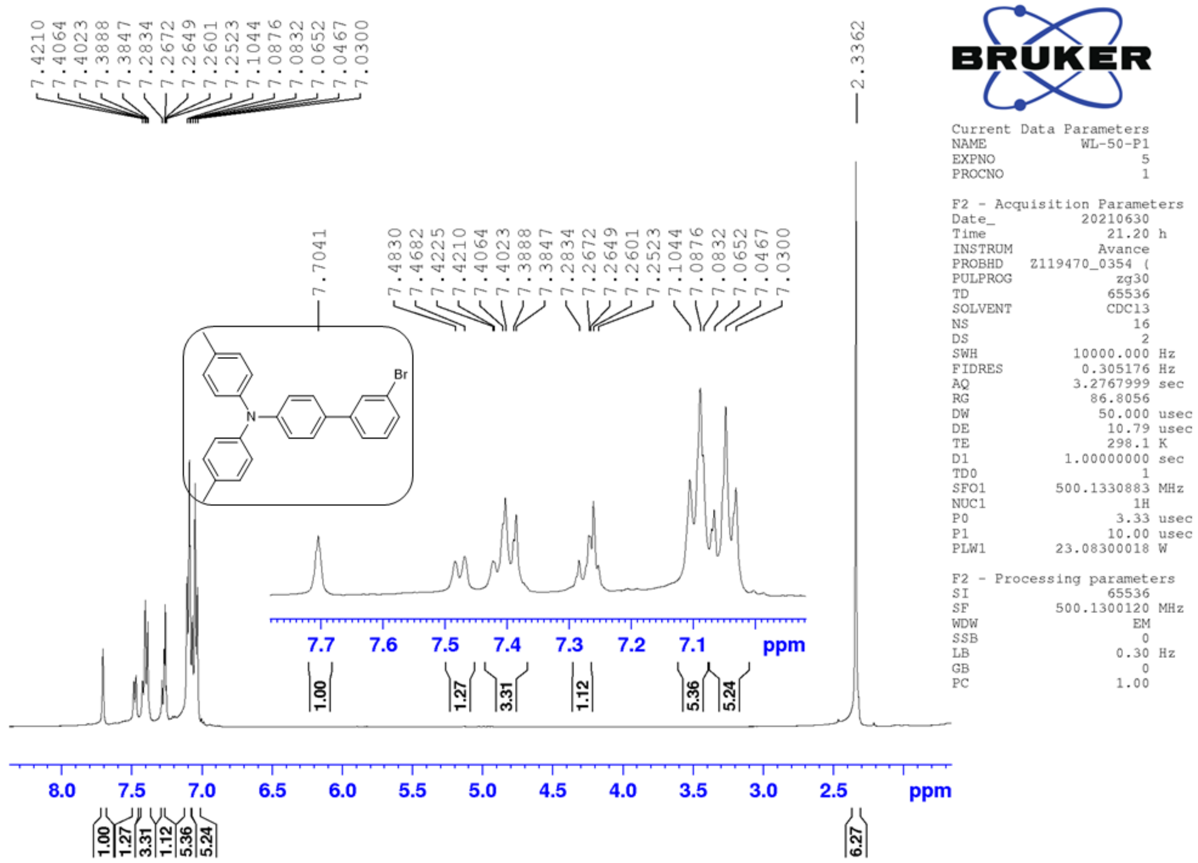

^{1}H NMR spectrum of DA-*m*Benz

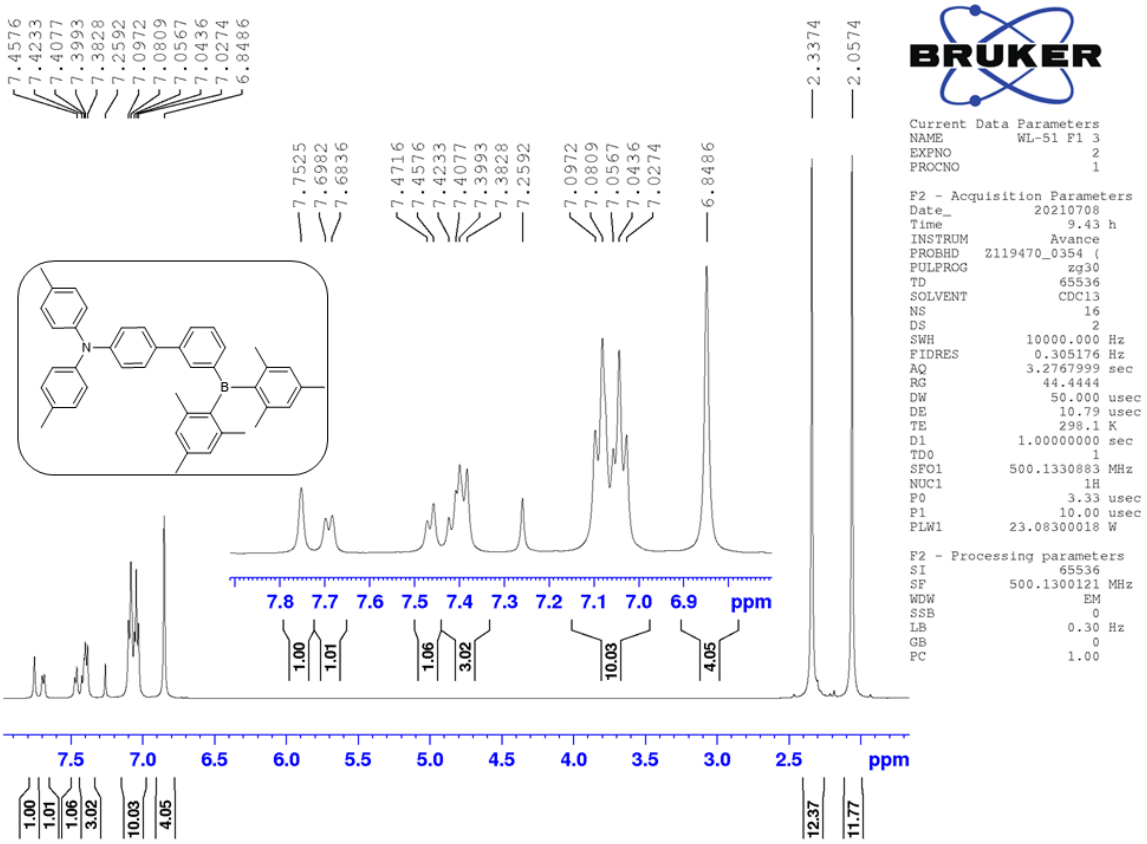

$^{13}C\{^1H\}$ NMR spectrum of DA-*m*Benz

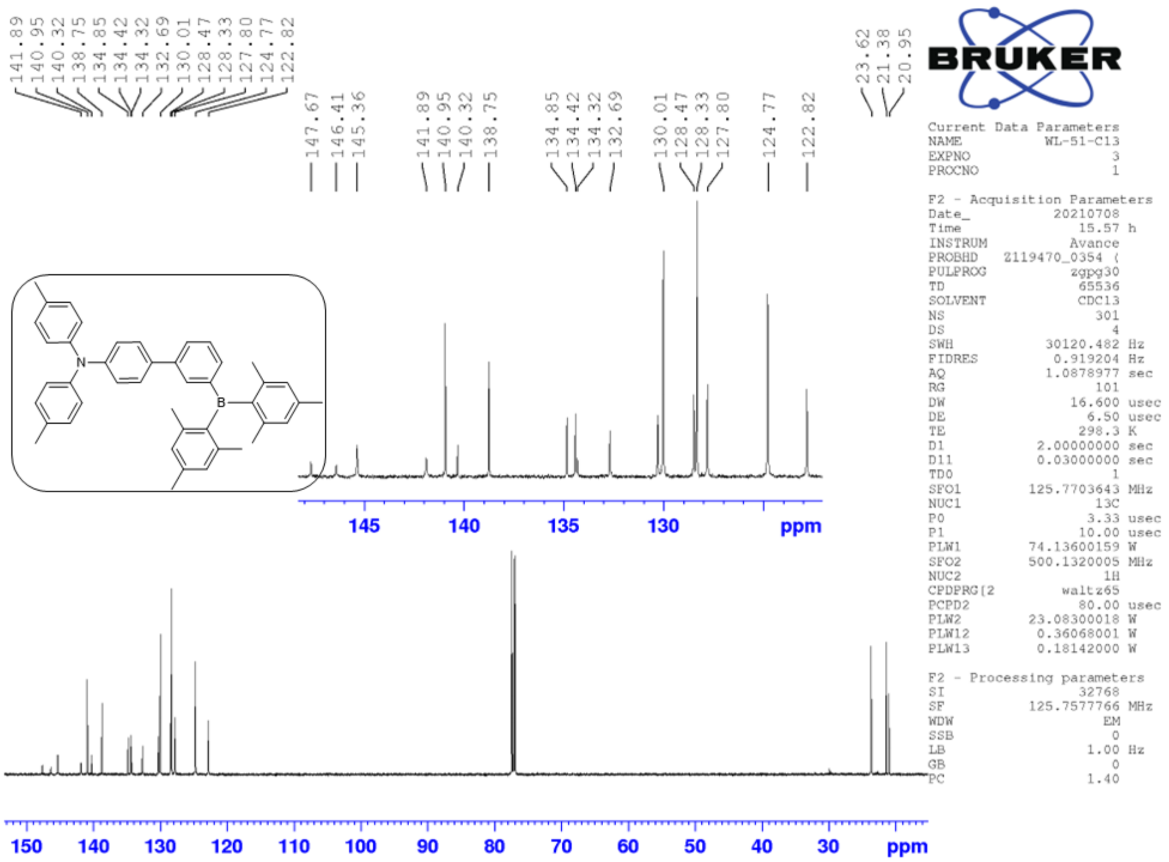

HRMS of DA-*m*Benz

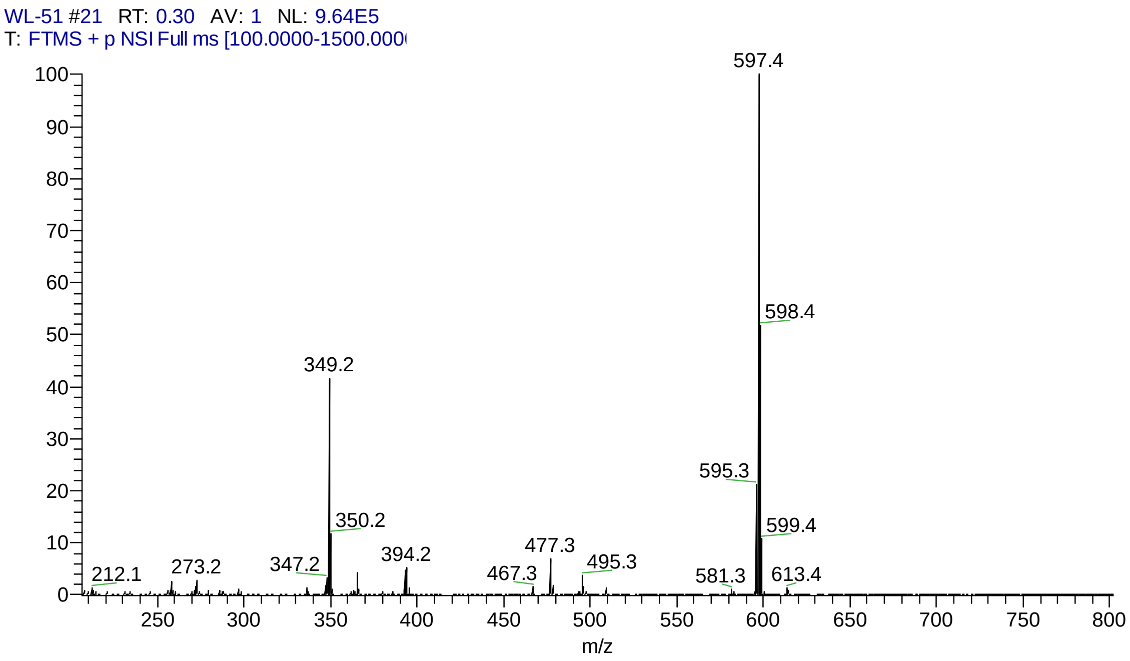

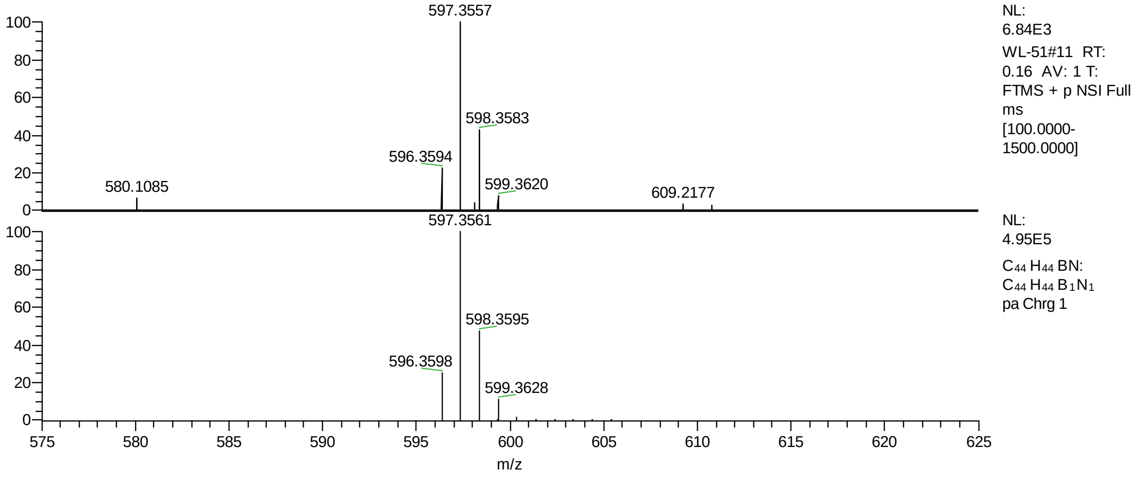

4. Reference

1. Pelter A, Smith K, Brown HC (eds). *Borane Reagents*. Academic Press: New York, 1988.
2. Kanazawa Y, Yokota T, Ogasa H, Watanabe H, Hanakawa T, Soga S*, et al.* Chemoselective amination of bromoiodobenzenes with diarylamines by palladium/Xantphos or ligand-free copper catalysts. *Tetrahedron* **71,** 1395-1402(2015).
3. Fan M, Jia L, Pang M, Yang X, Yang Y, Kamel Elyzayati S*, et al.* Injectable Adhesive Hydrogel as Photothermal-Derived Antigen Reservoir for Enhanced Anti-Tumor Immunity. *Adv. Funct. Mater.* **31,** 2010587(2021).
4. Auerhammer N, Schulz A, Schmiedel A, Holzapfel M, Hoche J, Röhr MIS*, et al.* Dynamic exciton localisation in a pyrene–BODIPY–pyrene dye conjugate. *Phys. Chem. Chem. Phys.* **21,** 9013-9025(2019).
5. Mieczkowski M, Steinmetzger C, Bessi I, Lenz A-K, Schmiedel A, Holzapfel M*, et al.* Large Stokes shift fluorescence activation in an RNA aptamer by intermolecular proton transfer to guanine. *Nat. Commun.* **12,** 3549(2021).
6. Frisch MJ, Trucks GW, Schlegel HB, Scuseria GE, Robb MA, Cheeseman JR*, et al.* Gaussian 16. *Revision A.03* ed. Gaussian, Inc.: Wallingford, CT; 2016.
7. Santoro F, Lami A, Improta R, Bloino J, Barone V. Effective method for the computation of optical spectra of large molecules at finite temperature including the Duschinsky and Herzberg-Teller effect: the Qx band of porphyrin as a case study. *J. Chem. Phys.* **128,** 224311(2008).
8. Lu T, Chen F. Multiwfn: a multifunctional wavefunction analyzer. *J. Comput. Chem.* **33,** 580-592(2012).
9. Lippert E. Habilitationsschrift Zur Erlangung Der Lehrberichtigung (Venia Legendi) Fur Das Fach Physikalische Chemie Ander Techischen-Hochschule-Stuttgart - Spektroskopische Bestimmung Des Dipolmomentes Aromatischer Verbindungen Im Ersten Angeregten Singulettzustand. *Z. Elektrochem.* **61,** 962-975(1957).
10. Stahl R, Lambert C, Kaiser C, Wortmann R, Jakober R. Electrochemistry and photophysics of donor-substituted triarylboranes: symmetry breaking in ground and excited state. *Chem. Eur. J.* **12,** 2358-2370(2006).

11. Marciniak H, Auerhammer N, Ricker S, Schmiedel A, Holzapfel M, Lambert C. Reduction of the Fluorescence Transition Dipole Moment by Excitation Localization in a Vibronically Coupled Squaraine Dimer. *J. Phys. Chem. C* **123,** 3426-3432(2019).
12. Gould IR, Young RH, Mueller LJ, Albrecht AC, Farid S. Electronic-Structures of Exciplexes and Excited Charge-Transfer Complexes. *J. Am. Chem. Soc.* **116,** 8188-8199(1994).
13. Ji L, Krummenacher I, Friedrich A, Lorbach A, Haehnel M, Edkins K*, et al.* Synthesis, Photophysical, and Electrochemical Properties of Pyrenes Substituted with Donors or Acceptors at the 4- or 4,9-Positions. *J. Org. Chem.* **83,** 3599-3606(2018).
14. Harder RA. The Photophysics and Electrochemistry of Carboranes. Ph.D. thesis, Durham University, Durham, U.K., 2012.
15. Fox MA, MacBride JAH, Peace RJ, Wade K. Transmission of electronic effects by icosahedral carboranes; skeletal carbon-13 chemical shifts and ultraviolet–visible spectra of substituted aryl-*p*-carboranes (1,12-dicarba-*closo*-dodecaboranes). *J. Chem. Soc., Dalton Trans.* 401-412(1998).
16. Kahlert J, Böhling L, Brockhinke A, Stammler H-G, Neumann B, Rendina LM*, et al.* Syntheses and reductions of C-dimesitylboryl-1,2-dicarba-*closo*-dodecaboranes. *Dalton Trans.* **44,** 9766-9781(2015).
17. Fox MA, MacBride JAH, Wade K. Fluoride-ion deboronation of *p*-fluorophenyl-*ortho*- and -*meta*-carboranes. NMR evidence for the new fluoroborate, $HOBHF_2^-$. *Polyhedron* **16,** 2499-2507(1997).
18. Fox MA, Wade K. Cage-fluorination during deboronation of meta-carboranes. *Polyhedron* **16,** 2517-2525(1997).
